# Cerebral organoids reveal early cortical maldevelopment in schizophrenia—computational anatomy and genomics, role of FGFR1

**DOI:** 10.1038/s41398-017-0054-x

**Published:** 2017-11-17

**Authors:** E. K. Stachowiak, C. A. Benson, S. T. Narla, A. Dimitri, L. E. Bayona Chuye, S. Dhiman, K. Harikrishnan, S. Elahi, D. Freedman, K. J. Brennand, P. Sarder, M. K. Stachowiak

**Affiliations:** 1 Department of Pathology and Anatomical Sciences, State University of New York at Buffalo, Buffalo, NY USA; 2 Department of Biology, State University of New York at Fredonia, Fredonia, NY USA; 3 Department of Biomedical Engineering, State University of New York at Buffalo, Buffalo, NY USA; 4 Icahn School of Medicine at Mount Sinai, Departments of Psychiatry and Neuroscience, New York, NY USA

## Abstract

Studies of induced pluripotent stem cells (iPSCs) from schizophrenia patients and control individuals revealed that the disorder is programmed at the preneuronal stage, involves a common dysregulated mRNA transcriptome, and identified Integrative Nuclear FGFR1 Signaling a common dysregulated mechanism. We used human embryonic stem cell (hESC) and iPSC-derived cerebral organoids from four controls and three schizophrenia patients to model the first trimester of in utero brain development. The schizophrenia organoids revealed an abnormal scattering of proliferating Ki67+ neural progenitor cells (NPCs) from the ventricular zone (VZ), throughout the intermediate (IZ) and cortical (CZ) zones. TBR1 pioneer neurons and reelin, which guides cortico-petal migration, were restricted from the schizophrenia cortex. The maturing neurons were abundantly developed in the subcortical regions, but were depleted from the schizophrenia cortex. The decreased intracortical connectivity was denoted by changes in the orientation and morphology of calretinin interneurons. In schizophrenia organoids, nuclear (n)FGFR1 was abundantly expressed by developing subcortical cells, but was depleted from the neuronal committed cells (NCCs) of the CZ. Transfection of dominant negative and constitutively active nFGFR1 caused widespread disruption of the neuro-ontogenic gene networks in hESC-derived NPCs and NCCs. The *fgfr1* gene was the most prominent FGFR gene expressed in NPCs and NCCs, and blocking with PD173074 reproduced both the loss of nFGFR1 and cortical neuronal maturation in hESC cerebral organoids. We report for the first time, progression of the cortical malformation in schizophrenia and link it to altered FGFR1 signaling. Targeting INFS may offer a preventive treatment of schizophrenia.

## Introduction

Although the primary onset of schizophrenia is during adolescence to young adulthood, an early developmental origin of schizophrenia has been implied by improperly clustered immature neurons in cortical layers II, III, and V^1^, changes in the hippocampal nonpyramidal CA2 neurons^2^, as well as hypoplastic midbrain dopamine neurons^3–6^. Such changes in brain structure most likely develop in utero during the late first and early second trimester^7–9^. Additionally, disorganization of the white matter tract has been observed in schizophrenia^10^, suggesting that the disease affects not only the development and function of neurons, but also their projections. The variability in brain malformations are thought to underlie the variety of clinical findings: positive symptoms (delusions and hallucinations), negative symptoms (affective flattening, amotivation, and anhedonia)^11,12^, and cognitive symptoms (disorganized speech and cognitive deficits) (DSM 4th edition). Abnormal development during the first trimester is also consistent with minor physical anomalies associated with schizophrenia^13^.

According to the neurodevelopmental hypothesis, both genetic and environmental factors broadly affect brain development and thus, contribute to the etiology of schizophrenia^7^. The ontogenic construction of the human cortex is thought to proceed through a series of irreversible cycles of neural stem and progenitor cell proliferation, differentiation to neuroblasts, migration to the brain surface where early neuronal-like cells are formed, and develop into mature neurons with intercellular connections and communicative pathways. Normal human brain development begins with proliferation of neurogenic neuroepithelial precursors, which act as neural stem cells and give rise to the primary progenitor cells, radial glia, that are capable of generating neurons, astrocytes, and oligodendrocytes^14^. In the developing vertebrate brain, the elongated bipolar radial glia are located at the apical surface of the ventricular zone (VZ) and span the width of the cortex. These cells generate translocating radial glia, intermediate progenitor cells, and neuroblasts that migrate through the intermediate zone (IZ) to the outer cortical zone (CZ), and populate cortical layers. Via this inside-out process, the early born neurons occupy inner layers while the late born neurons migrate out toward the edge and occupy the superficial cortical layers^15^. Disruption of these processes has been hypothesized to underlie the misconstruction of the cortex and subcortical circuits observed in autism^16^ and schizophrenia (review-^17^).

Coordinated transition of cells from one neurodevelopmental stage to another are needed to produce synchronized development of the cortical layers. The navigation through distinct developmental stages involves concerted regulation of thousands of genes and is likely overseen by some central guiding mechanism(s). Our recent studies have revealed one such pan-ontogenic mechanism, Integrative Nuclear FGFR1 Signaling (INFS) that controls general body and the brain development^18–20^. The *fgfr1* gene resides at the top of the gene hierarchy that governs gastrulation, as well as the subsequent development of the major body axes, nervous system, muscles, and bones, by affecting downstream genes that control the cell cycle, pluripotency, and differentiation, as well as microRNAs (miRNAs)^21–26^. Studies show that this regulation is executed by nuclear FGFR1 (nFGFR1), which integrates signals from diverse development-initiating factors, cooperates with a multitude of transcription factors (TFs), and targets thousands of genes encoding for mRNAs, as well as miRNAs in top ontogenic networks^19,20^. nFGFR1 binds to the promoters of genes that control the transition from proliferation to cell differentiation, as well as to the morphogens that delineate the body and CNS axes, and construct the nervous system^18,19,27–29^.

RNA-sequencing of differentiating neuronal progenitor cells (NPCs) developed from the induced pluripotent stem cells (iPSCs) of four different schizophrenia patients and four control subjects revealed a common dysregulation of 1384 mRNAs and 18 miRNA genes, deconstruction of coordinated mRNA, and miRNA–mRNA networks, and the formation of aberrant networks^29^. The downregulated genes reside within pathways that block neurogenesis, e.g. Notch1 and Rest, while the upregulated genes were found in pathways that initiate neural development, including master genes, such as Ascl1, Nur77, and RXR, or genes of the Wnt signaling pathway^29^. ChIPseq revealed that nFGFR1 targets more than 80% of the dysregulated mRNA genes and 31% of dysregulated miRNA genes^29^. These results indicated an early (preneuronal) genomic etiology of schizophrenia and designated INFS as a common dysregulated mechanism in the Cannon and Keller water-shed model of schizophrenia^29,30^. Analyses of the differentiated neurons of the same schizophrenia and control iPSC lines revealed impaired migration in 2D cultures^31^, polarity^32^, decreased neurite numbers^33^, reduced synaptic maturation^33–36^, and decreased activity in the schizophrenia neurons^35,36^. These changes forecasted a potential disruption of early brain development in schizophrenia. Therefore, in the present study we followed the neuro-ontogenic potency of these characterized iPSC lines, from the stem cell/progenitor stage through the formation of cortical layers using the 3D cerebral organoid model.

Pluripotent stem cells have the potential to differentiate into various cell lines^37^ and to self-organize and develop organ-like structures termed “organoids.” Recently, 3D cerebral organoids have been generated, which recapitulate key aspects of human brain development during the first and early second trimesters of embryogenesis^38^. The pluripotent hESCs or iPSCs aggregate to form embryoid bodies (EBs) capable of generating all three germ layers^39^. EBs placed in neuroectodermal media form the neuroectoderm germ layer, the foundation for the development of cerebral organoids. Neuroectoderm cultured in matrigel scaffolds develop a 3D cerebral organoid tissue with distinct germinal centers (rosettes) and cortical layers corresponding to the human brain structure at the end of the first trimester^38,40,41^. This new technology applied to iPSCs from microcephaly patients^41^ and patients with autism^16^ revealed disruptions in early brain development and shed light on underlying cellular mechanisms involved.

In the present investigation, the cerebral organoid technology applied to control and schizophrenia iPSCs^29,31,33^ provided for the first time, an insight into the early stages of the telencephalic development and the role of FGFR1 in schizophrenia.

These results showed an abnormal dispersion of the proliferating stem-like cells outside the VZ into the IZ and CZ, and a curtailed neuronal cortical development in the CZ of the schizophrenia cerebral organoids accompanied by turning off the expression of nFGFR1. The arrest of cortical development was reproduced in control cerebral organoids derived from hESCs, by blocking and depleting FGFR1 with its specific antagonist, PD173074. This study suggests that a reconstitution of nFGFR1 in developing cortical neurons can modify the developmental malformations.

## Materials and methods

### Pluripotent stem cells

In the human embryonic stem cell (hESC) studies, line H9 (WA09) (WiCell Research Institute, Inc.) and line HUES8 (originated from Harvard University, Boston, USA) were used. The iPSC lines used in the present study (Table 1) were derived from different control individuals and from individuals diagnosed with schizophrenia at different ages^33^. In earlier studies these schizophrenia lines displayed similar phenotypic changes in 2D cultures as described in the section “Introduction”^31,33,42^. Recent RNAseq investigation showed common dysregulation of a global transcriptome in neural progenitor cells (NPCs) differentiated from all schizophrenia lines and ChIPseq showed similar changes in nFGFR1 genomic interactions in two investigated schizophrenia iPSC lines^29^. In the present study, we found no discernable differences between the organoids generated from control female and control male iPSC lines and between schizophrenia female and control male iPSC lines. The differences between schizophrenia and control organoids were reproduced in all control and schizophrenia iPSC lines and were statistically evaluated.

**Table 1:** iPSC lines used to generate organoids^29,33^

Cell line ID	Gender	Description	Age of diagnosis
2038	Male	iPSC SCZ	6 years
2497	Male	iPSC SCZ	15 years
1835	Female	iPSC SCZ	27 years
BJ #1	Male	iPSC Control	
3440	Male	iPSC Control	
2937	Male	iPSC Control	
3651	Female	iPSC Control	

Plasmids and transfections are described in Supplemental Methods.

### Generation of cerebral organoids

Cerebral organoids were generated from hESCs (H9 and HUES8) and from human iPSCs using a modified protocol of Lancaster et al.^40^, described in the Supplementary Methods and in Supplementary Fig. 1. Eight-day hESC (H9) cerebral organoids were treated with the FGFR1 inhibitor, PD173074 (Abcam), by adding fresh media with 100 nM PD173074 or drug-free media every 2 days for 10 days. On day 18, the cerebral organoids were harvested, fixed, and processed for immunocytochemistry.

### Pulse BrdU labeling

At day 14, cerebral organoids (controls or PD173074 treated) were treated with 100 µM BrdU for 2 h (pulse phase), washed with DMEM-F12 three times and then incubated with media (control or PD173074) for an additional 4 days (chase phase). At day 18, organoids were harvested and double immunostained with rat anti-BrdU and mouse anti-Pan-Neu antibodies, as described in the next section.

### Organoid sectioning and immunocytochemical staining

For all experimental conditions (four controls and three schizophrenia iPSC lines, hESC H9 and HUES8) 4–5 organoids for each line were cryosectioned at 30 μm thickness and processed as described in the Supplementary Methods. The antibodies used are listed in Supplementary Table 1. The control and experimental organoid sections were immunostained at the same time using identical protocols^28,43,44^. Each stain was performed independently four times.

### Quantitative analyses of histological sections and images

Cell were counted using Visiopharm stereological software^45^. Fluorescence intensity measurements (FIM) were performed using Zen 2.0 Blue Imaging software in randomly selected Regions of Interest (ROIs) within organoid regions listed in the figure legends. All images acquired were in the linear range of the camera. Images of sections from control and schizophrenia organoids were acquired under identical illumination conditions and identical camera gain, offset, and exposure times. Background images (outside the tissue sections) were taken and subtracted from the images of stained sections. The total area of each ROI image was recorded, as well as the number of thresholded pixels and their integrated intensity. Each channel was converted into the eight-bit grayscale and the mean intensity was calculated. Statistical analysis was performed using *t*-test in MiniTab software and data is presented as mean ± Standard Error of the Mean (SEM). For detailed protocols see Supplementary Methods.

Computational analyses were run on the combined sets of images and the initial results were presented to corresponding authors without identifying the groups (schizophrenia and control). The codes used in computational image analyses are provided on the following GitHub repositories:

Ki67 Analysis: https://github.com/TheJaeger/CellStats-Ki16

Calretinin Analysis: https://github.com/TheJaeger/CellStats-Calretinin

The code version used belongs to the master branch, any additional changes made reflect improvements to code readability. The code can be accessed by downloading or cloning the repository and can be run by opening *main.m* in MATLAB. Additonal instructions on running the codes are provided in *README.md* at the root of each repository.

All image files used in computational analyses can be found in the link below. Each corresponding set of images (Ki67 and calretinin) are grouped into control and schizophrenia sets.

Images: http://www.acsu.buffalo.edu/~mks4/public_sharing/schizophrenia2/

### RNAseq analysis

The analysis was performed using homogenous cultures of NPCs differentiated from hESC H9, as described in ref. 29. To induce neuronal committed cells (NCCs), NPCs were treated with 20 ng ml^−1^ BDNF (Peprotech), 20 ng ml^−1^ GDNF (Peprotech), 1 mM dibutyryl-cyclic AMP (Sigma), and 200 nM ascorbic acid (Sigma)^29^ for two days. Plasmids and transfections of NPCs are described in Supplemental Methods. NPCs were transfected with control DNA or FGFR1(SP-/NLS)(TK-) and 24 h later were stimulated for 48 h with neuronal differentiation media induced by cAMP/BDNF/GDNF. Experiments were performed using three biological replicates of culture and transfection conditions. The RNAseq protocol was described previously^19,29^ and is summarized in the Supplementary Methods. The results of RNAseq have been deposited on NCBI GEO with accession code: GSE103307.

### Statistical analysis

Statistical analysis was performed using two-sided *t*-test (comparison of two groups), by one or two-way ANOVA (comparison of four or more data groups) or by two-sample Kolmogorov–Smirnov test. *P* < 0.05 was considered significant.

The RNAseq data were statistically evaluated using Tuxedo pipeline as previously described^29^. The details of the sample sizes are listed in figure legends and fulfill the requirement of statistical significance.

## Results

### Cerebral organoids recapitulate cellular processes of cortical development

**Fig. 1:**
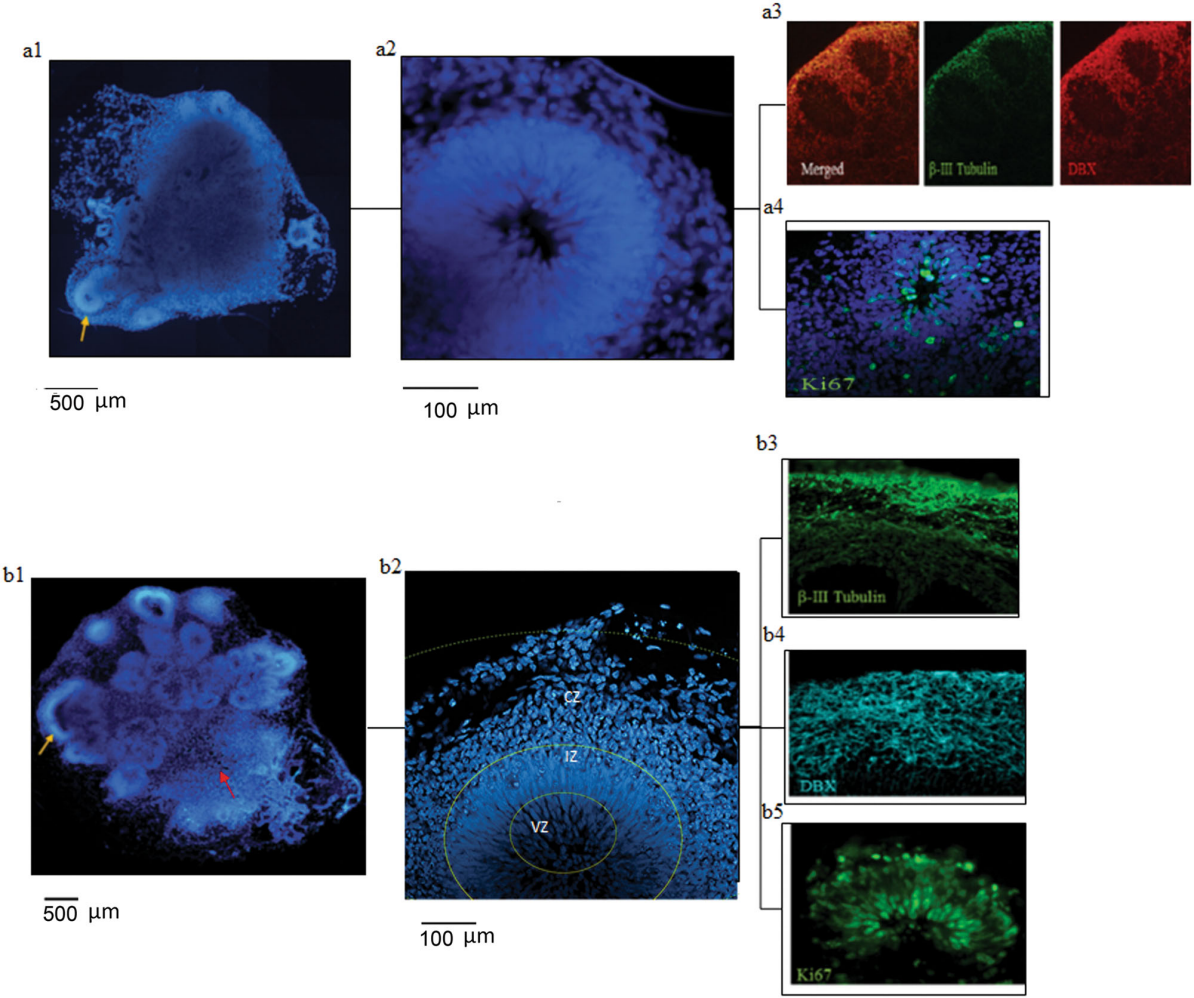
Representative hESC (H9) cerebral organoids at (a) 8 and (b) 18 days of development (a1, b1) tile scanning of DAPI; yellow arrows point to cortical rosettes, enlarged in a2 and b2; at day 18, zones are outlined—ventricular zone (VZ), intermediate zone (IZ), and cortical zone (CZ). Immunostaining: (A4, B5) Ki67+ proliferating cells; (a3, b3, b4), doublecortin (DCX+) neuroblasts, βIII-tubulin+ immature neurons

Cerebral organoids from hESCs were developed using a combination of two protocols (Supplementary Fig. 1a, Supplementary Methods). At day 8 the organoids formed stable cortical rosettes (Fig. 1, a1, a2) with proliferative Ki67 positive cells in the inner layer and a mixture of doublecortin positive (DCX+) neuroblasts and immature βIII-tubulin positive neurons in the outer layers (Fig. 1, a3, a4).

During the subsequent 10 days, the organoids increased in size and the number of developed rosettes increased two to three fold (Fig. 1,b1), as compared to 8-day old organoids (Fig. 1, a1). The 18-day organoids formed polarized structures with a distinguishable border that separated a forebrain-like region containing multiple rosettes from a hindbrain-like structure, which typically lacked rosettes (Fig. 1, b1), as reported previously^40^. In all of the 18-day organoids examined, the cortical rosettes developed three major zones distinguished by DAPI staining (Fig. 1, b2). The VZ contained a large ventricle-like lumen surrounded by compact layers of vertically aligned elongated cells. The area outside the VZ, the IZ, contained uniform, predominantly round cells. The outermost CZ contained horizontally aligned cortical layers (Fig. 1, b2).

Structures of the female hESC H9 line-derived organoids, internal rosettes, and the cellular organization (Fig. 1), were reproduced using the male hESC HUES8 line (Supplementary Fig. 1b).

We investigated the neurodevelopmental stages of cells in each zone by immunostaining for proliferating NPCs with the nuclear Ki67 antigen^46^, doublecortin (DCX) expressed by neuroblasts, and βIII-tubulin expressed by young, immature neurons (NCCs)^45^. These stainings revealed cellular organization consistent with the inside-out pattern of human neocortex development^15^ (Fig. 1, b3, b4 and b5). Proliferative Ki67+ cells (Fig. 1, b5, Supplementary Fig. 1b3) and GFAP-expressing radial glia (Supplementary Fig. 1b2) were mostly present in the VZ, similar to the developing brain in the ventricular and sub-VZs, where generation of new cells by the brain stem and progenitor cells takes place. Few Ki67+ cells were found in the IZ and proliferating cells were not detected in the CZ (Fig. 1, b5, Supplementary Fig. 1b3). The doublecortin+ neuroblasts were present in the IZ and the CZ, and the βIII-tubulin+ NCCs were found predominantly in the CZ.

### Organoids derived from schizophrenia iPSCs—dispersion of proliferating cells in the cortex and blockade of cortical neuronal development

**Fig. 2:**
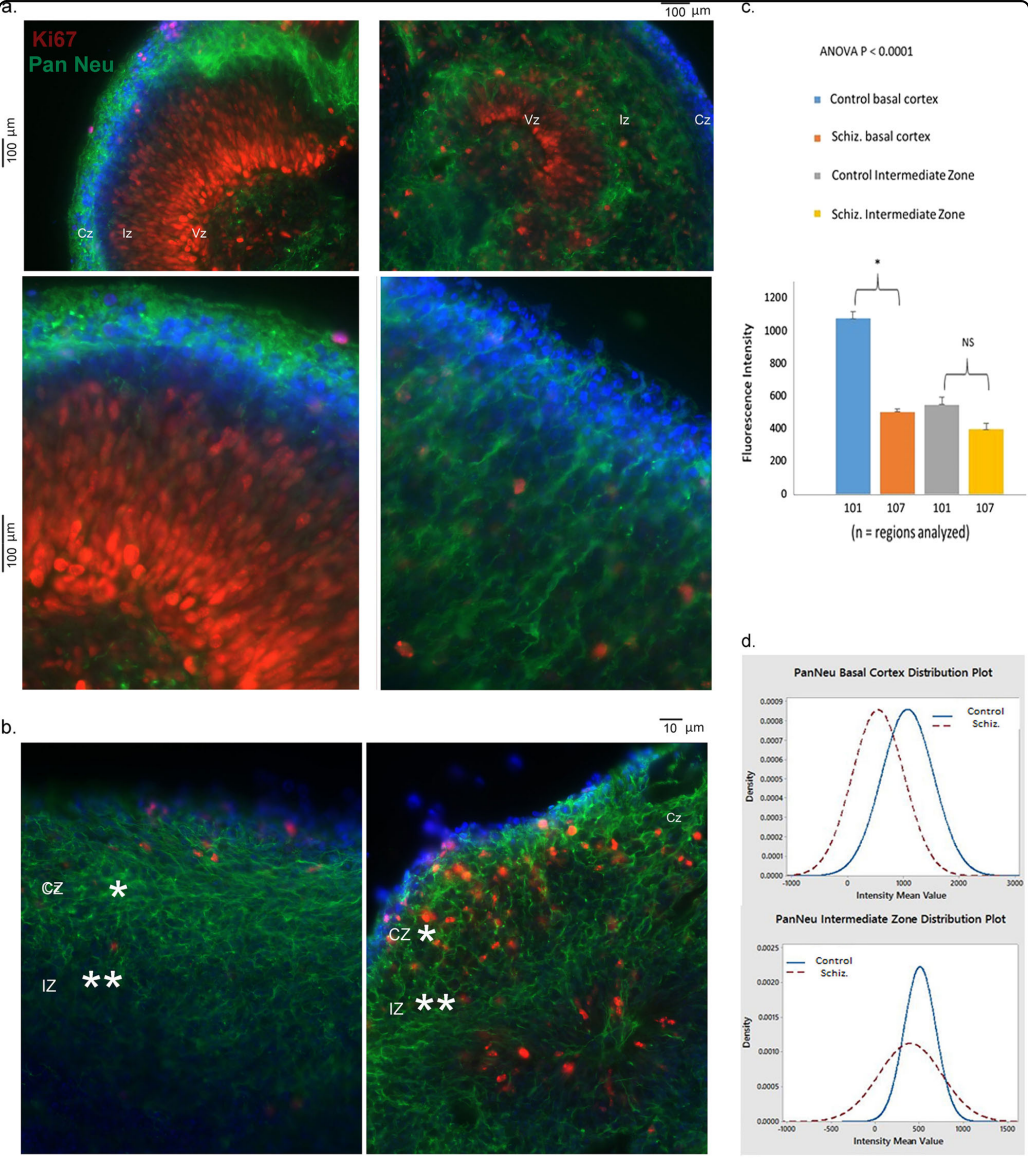
Disorganized migration of proliferating cells and depletion of cortical neurons in schizophrenia iPSC cerebral organoids Organoids were coimmunostained for Ki67 (red) and Pan-Neu (green). Nuclei were stained with DAPI (blue). **a** 2-week organoids—images show representative sections of organoids, control (iPSC line BJ1) and schizophrenia (iPSC line 1835). In schizophrenia organoids, note the dispersion of proliferating (Ki67+) cells outside the VZ into IZ and CZ, fewer mature Pan-Neu+ neurons in CZ, and the appearance of Pan Neu+ neurons in the IZ. **b** 5-week iPSC organoids: control (line 2937) and schizophrenia (line 2038)—representative images of control and schizophrenia organoids. In schizophrenia organoids, note dispersion of Ki67+ cells into CZ, reduced density of Pan Neu+ neurites in basal CZ and the presence of Pan Neu+ cells with neurites in the IZ. 3D rotational confocal images of control (line 3651) and schizophrenia (line 1835) organoids are shown in Video 1a and b. Pan-Neu immunofluorescence intensity was measured in multiple randomly selected ROI (1 × 10^2^ μm^2^ in basal cortex (*) and in IZ (**)). **c** 5-week organoids—Pan-Neu immunofluorescence intensity was measured in several ROIs (# shown on *y*-axis) of multiple organoids from three control and three schizophrenia patients. Note, significantly reduced Pan-Neu fluorescence intensity in basal cortex of the schizophrenia organoids and the lack of significant changes in the IZ. **d** Distribution of Pan-Neu intensity numbers in analyzed ROIs. Note the significant separation of the basal cortex plots in control and schizophrenia organoids and the lack of separation of the IZ plots

After establishing the protocol for the generation of hESC cerebral organoids, we applied this procedure to human iPSCs. To analyze whether early brain development may be altered in schizophrenia, we used iPSC lines reprogrammed from three schizophrenia and four control individuals, in which common dysregulated transcriptomes have been recently identified^29^. Our qualitative observations were verified by computational and statistical analyses of three schizophrenia and four control patients and are described below. In general, the iPSC cerebral organoids followed the developmental pattern and displayed cortical rosettes that were similar to the hESC organoids. At 5 weeks, the rosettes of both control and schizophrenia iPSC organoids had only narrow residual lumens (Supplementary Fig. 2). No gross size differences were observed between control and schizophrenia iPSC organoids. However, a detailed cellular analysis revealed marked differences between control and schizophrenia organoids (Fig. 2). The control iPSC organoids, similar to hESC organoids, contained 2–3 layers of proliferating Ki67+ NPCs in the VZ, few Ki67+ cells in the IZ, and none or only single proliferating cells in the CZ. These layers were already observed at week 2 (Fig. 2a) and were further developed at week 5 (Fig. 2b). In contrast, in the 2-week schizophrenia organoids, only one layer of Ki67+ cells was typically present and there were no distinct palisades of proliferating cells surrounding the VZ lumens observed. Instead, the Ki67+ cells were strikingly dispersed in the IZ, as well as in the CZ (Fig. 2a).

Staining with a monoclonal Pan-Neu antibody, which reacts with key somatic, nuclear, dendritic, and axonal proteins of the pan-neuronal architecture, revealed differentiated Pan-Neu+ neurons concentrated in the CZ of the control iPSC organoids, forming a distinct cortical layer at 2 weeks (Fig. 2a) and more pronounced at 5 weeks (Fig. 2b). These mature neurons formed a dense network of long processes parallel, as well as perpendicular to the cortical surface. At 2 weeks, the schizophrenia iPSC organoids had noticeably fewer Pan-Neu neurons and Pan-Neu positive dendrites in the CZ (Fig. 2a, Supplementary Fig. 2b). Instead, the schizophrenia organoids displayed differentiated Pan-Neu+ neurons deep within the IZ and VZ regions. These mature subcortical neurons were found already at 2 weeks in the schizophrenia organoids, at the time when no such neurons were observed in the control organoids (Fig. 2a, Supplementary Fig. 2b). At 5 weeks, the basal cortical Pan-Neu positive neurons in schizophrenia organoids displayed dense short processes, different from the network of the long processes formed in the cortex of the control organoids (Fig. 2b). Overall, density of the Pan-Neu fibers in schizophrenia appeared reduced. This decrease was verified by measuring the Pan-Neu fluorescence intensity in the basal CZ, as compared to the IZ. In three patients (compared to three control individuals), we found a significant reduction in Pan-Neu fluorescence in the basal CZ and no significant change in the IZ (Fig. 2c, d).

**Fig. 3:**
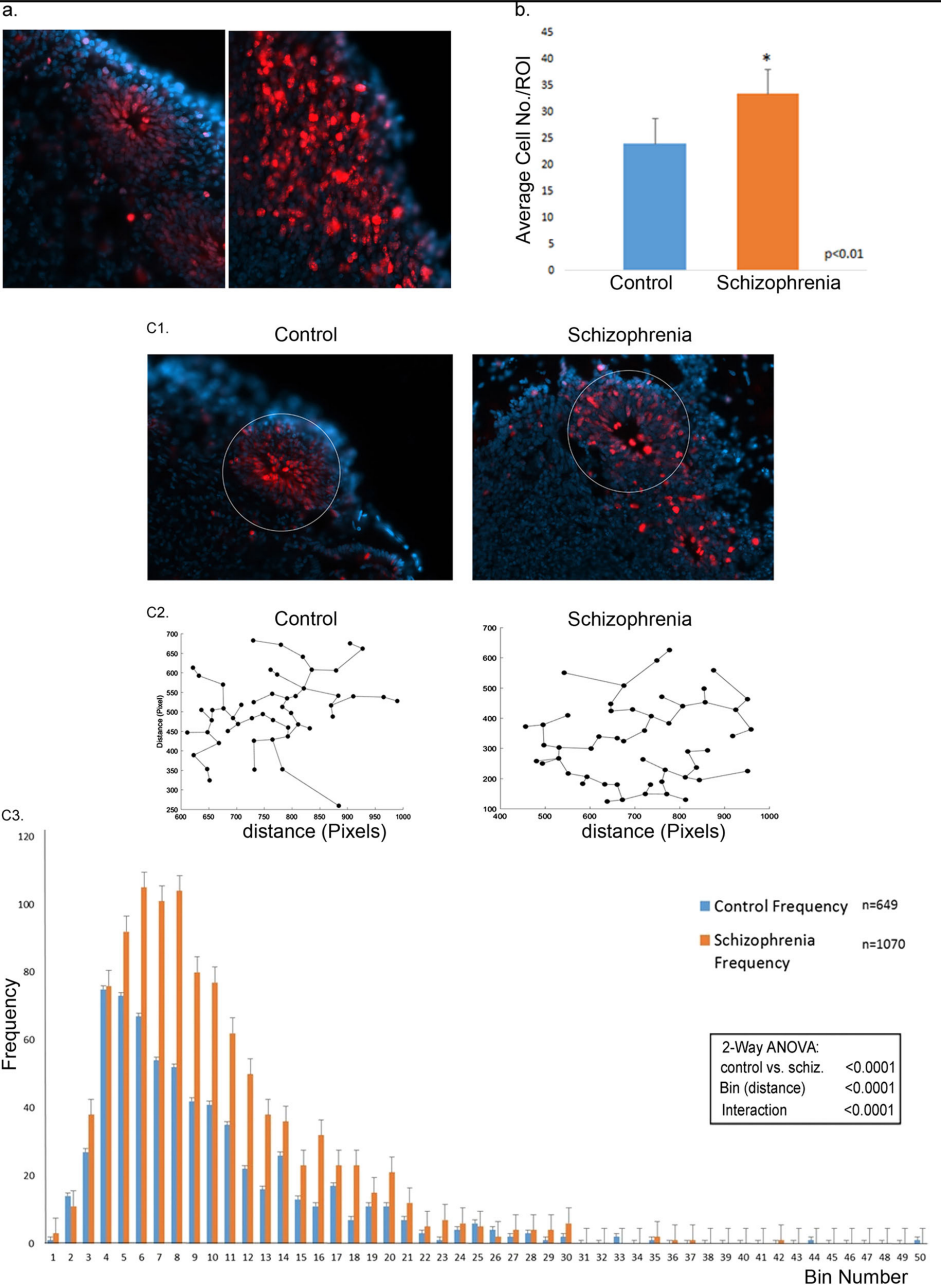
Quantification of disorganized migration of proliferating NPCs in schizophrenia compared to control cerebral organoids **a** Exemplary images showing Ki67+ (red) proliferating NPCs in the center of the rosette of a control organoid (line 2937) and their dispersion in a schizophrenia organoid (line 2038) (nuclei were stained with DAPI). **b** Increased density of proliferating cells in schizophrenia organoids. ROIs were outlined on organoid images from three control and three schizophrenia patients, as shown in (c1). Bar graph shows significantly higher average numbers of the KI67+ proliferating cells in schizophrenia ROIs than in control ROIs (17 control and 20 schizophrenia ROIs quantified). **c** Global Minimum Spanning Tree (MST) analysis of Ki67+ NPC dispersion within ROIs (c1—examples) was carried out using 17 control and 20 schizophrenia ROIs from three control cases and three schizophrenia patients (total of 649 and 1070 cells analyzed, respectively). The shortest connecting edges between cells were identified in pixels (c2) using MST calculating program and were grouped into bins (c3). Bin 1 contains edges of 0–5 pixels, bin 2 of 5–10 pixels, etc. Frequency indicates average numbers of cells per bin in all ROIs measured. Schizophrenia organoids displayed a shift towards longer MST distances. Two-Way ANOVA showed a significant interaction between organoid phenotype (control vs. disease) and the MST distances

The impaired development of the schizophrenia cortical neurons was accompanied by a visible dispersion of the proliferating NPCs (Fig. 2a, b). To quantitatively asses these changes, we analyzed images of the organoids form three controls and three patients (Fig. 3). Computer-based cell identification and automated counting showed a significant increase in the density of Ki67+ cells/area of the schizophrenia ROIs (Fig. 3b). For each ROI (example in Fig. 3, c1), we generated Minimum Spanning Tree graphs (Fig. 3, c2) (Supplementary Methods). This analysis revealed significant increases in the distances between cells, i.e., NPC dispersion, in the schizophrenia organoids. Two-Way ANOVA demonstrated a highly significant relation between disease and cell migration (Fig. 3, c3).

To quantitatively assess changes involving the Ki67+ NPCs, we analyzed images of the organoids of three controls and three patients. Computer-based cell identification and counting showed a significant increase in the density of Ki67+ cells/area in the analyzed ROIs (Fig. 2b). For each ROI, Minimum Spanning Tree graphs were generated (Fig. 2, c1, c2), which showed significant increases in the distances between cells, i.e., increased NPC dispersion, in the schizophrenia organoids (Fig. 2, c3). Two-Way ANOVA demonstrated a highly significant relation between disease and cell migration.

Together, our observations showed proliferation and migration of the schizophrenia NPCs, premature development of neurons in the subcortical region, and impaired neuronal development in the forming cortex of the schizophrenia organoids.

### Changes in NPCs in schizophrenia iPSC organoids

**Fig. 4:**
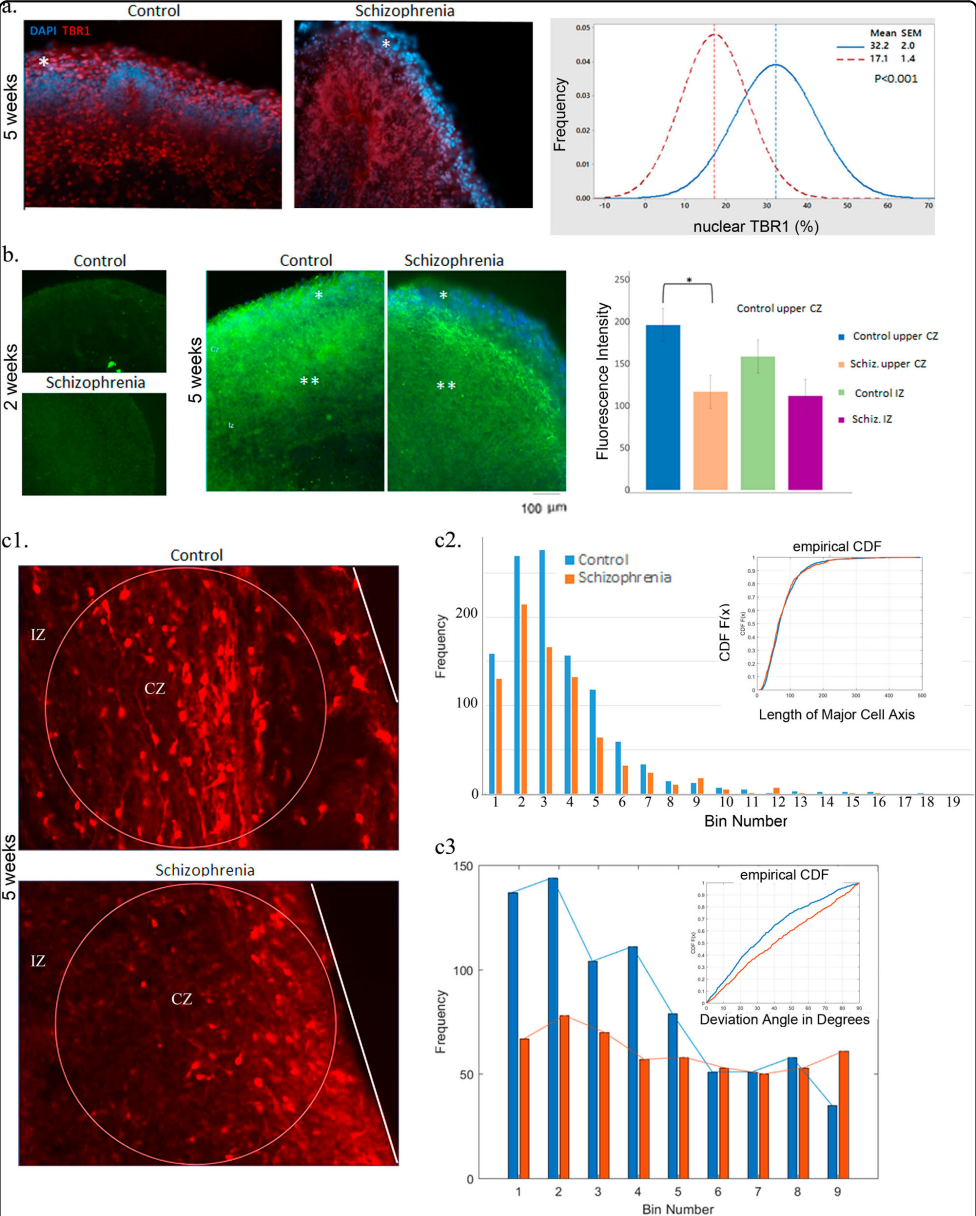
**(a)** Decreased nuclear TBR1 (red) expression in the upper cortical zone of 5-week schizophrenia organoids Nuclei were stained with DAPI. Images show representative sections of control (iPSC line BJ1) and schizophrenia (iPSC line 2038) organoids. Total number of DAPI-stained nuclei and the number of nuclei expressing TBR1 were counted in multiple randomly selected ROI (5 × 10^3^ μm^2^, ∼50 cells/ ROI) within the upper cortical layers (*6 cells deep) of three control individuals and three patients. Percent of (TBR1 + DAPI)/DAPI-stained nuclei was determined for each ROI. Graph shows distribution of the % of TBR1 expressing cells in the individual ROIs (26 control and 33 schizophrenia ROIs). The difference between control and schizophrenia mean values was significant (*t-*test). Individual value plots are shown in Supplementary Fig. 5b. **b** Decreased reelin expression in schizophrenia organoid cortex. Images show control (BJ1) and schizophrenia (1835) organoids. Note the lack of reelin staining in 2-week organoids. In 5-week organoids, reelin immunofluorescence intensity was determined in randomly selected ROIs (3 × 10^3^ μm^2^) in the upper CZ (*) and in the IZ (**) regions of three control individuals and three patients using Zen 2.0 Blue Imaging software (22 control and 17 schizophrenia upper CZ ROIs and the same number of IZ ROIs). ANOVA of four groups followed by Tukey posthoc test showed a significant decrease in the reelin expression in the schizophrenia upper CZ and a lack of significant differences between control and schizophrenia in the IZ. Individual value plots are shown in Supplementary Fig. 5. **c** Morphology and orientation of cortical calretinin interneurons. c1—images of control and schizophrenia organoids. A total of 770 control and 547 schizophrenia calretinin interneurons were measured in 20 and 16 ROIs, respectively, in the organoids from three control and three schizophrenia patients. The average cell density (*d* = number of cells/ROI) was not significantly different between control and schizophrenia (Supplementary Fig. 3a, b). c2—graph shows cell distribution (cumulative frequency) relative to their total length, including the cell body and neurites. An average cell body had a length of ∼50 pixels, 18 μm. A two-sample Kolmogorov–Smirnov test of cumulative density function (CDF shown in the inset) of control and schizophrenia groups found no significant difference between the length of control and schizophrenia interneurons. c3—angles between the long axis of each cell and the cortical surface organoids were computed as described in the Supplementary Methods. Graph shows distribution of cells (cumulative frequency) in bins corresponding to the deviation angles from the cortical surface. A two-sample Kolmogorov–Smirnov CDF test (CDFs shown in the inset) of control and schizophrenia groups yielded a highly significant difference (*p*-value of <13.9 × 10^−7^) between the orientation of control and schizophrenia interneurons, relative to the cortical surface

The antibody against autism-linked transcription factor T-Box Brain 1 (TBR1), identifies developing neuroblasts of the subplate (SP) and cortical plate (CP), which provide the first pioneer neurons of the developing cerebral cortex^47^. TBR1 is necessary for neuronal differentiation of NPCs and is a potential master regulator in autism spectrum disorders^48^. At 5 weeks of control iPSC organoid development, cells expressing nuclear TBR1 were distributed throughout the entire CZ and IZ (Fig. 4, a1). In contrast, in schizophrenia organoids, TBR1+ cells were absent from the upper cortical region, while cells expressing high levels of TBR1 were found concentrated predominantly in the cortical plate and IZ. The loss of TBR1 expression from the upper cortical layers was documented in all the three schizophrenia organoids, as compared to three controls. Quantitative stereological counting showed 32.2 + 2.0% of DAPI-stained nuclei were positive for TBR1 in organoids from control subjects. This number was significantly reduced in the schizophrenia organoids to 17.1 + 1.4 (*p* < 0.001) (Fig. 4, a2, Supplementary Fig. 5c). Thus, impaired development of the cortical neurons is associated with an overexpression of TBR1 in the cortical plate and the absence of the superficial pioneer TBR1+ neuroblasts.

We next analyzed expression of reelin, a secreted glycosylated protein deposited by the neurons of the marginal zone and other neurons in the developing cortex. Extracellular reelin supplies essential migratory cues to the waves of the developing cortico-petal neuroblasts and immature neurons and for the formation of the cortical layers^49^. In 2-week control organoids, reelin was not expressed, but at 5 weeks, reelin expression was detected in cells dispersed within the CZ, including its marginal layer, as well as in the IZ (Fig. 4b). Quantitative analysis of reelin immunofluorescence intensity in organoids from three patients and three controls showed a consistent depletion (−40%, *p* < 0.001) of reelin in the schizophrenia cortex (Fig. 4b, Supplementary Fig. 5b). A small reelin decrease in the IZ was observed in some organoids, but did not attain statistical significance (Fig. 4b, Supplementary Fig. 5b).

### Loss of directionality of calretinin interneurons in schizophrenia organoids

Calretinin is a cytoplasmic protein abundantly expressed by cortical and retinal interneurons^50^. At 5 weeks, the calretinin interneurons were abundant in the CZ (Fig. 4c), and less abundant in the IZ in control organoids (Fig. 4e). The density of the calretinin neurons in schizophrenia organoids appeared somewhat reduced, but this change was not significant (Supplementary Fig. 3a and b). Also, the length of calretinin neurons, including processes, was not significantly different between the control and schizophrenia organoids (Fig. 3, c1, c2). Calretinin interneurons are known to form horizontal connections between cortical neurons and cortical fields^51^. Consistent with this function, calretinin+ processes ran predominantly parallel or near parallel to the cortical surface of the control organoids. In contrast, in schizophrenia organoids, this preferential horizontal directionality was lost. Thus, the formation of connections between cortical fields by the calretinin interneurons was diminished in the developing schizophrenia cortex.

### Expression of FGFR1 protein is reduced in the cortex of schizophrenia organoids

**Fig. 5:**
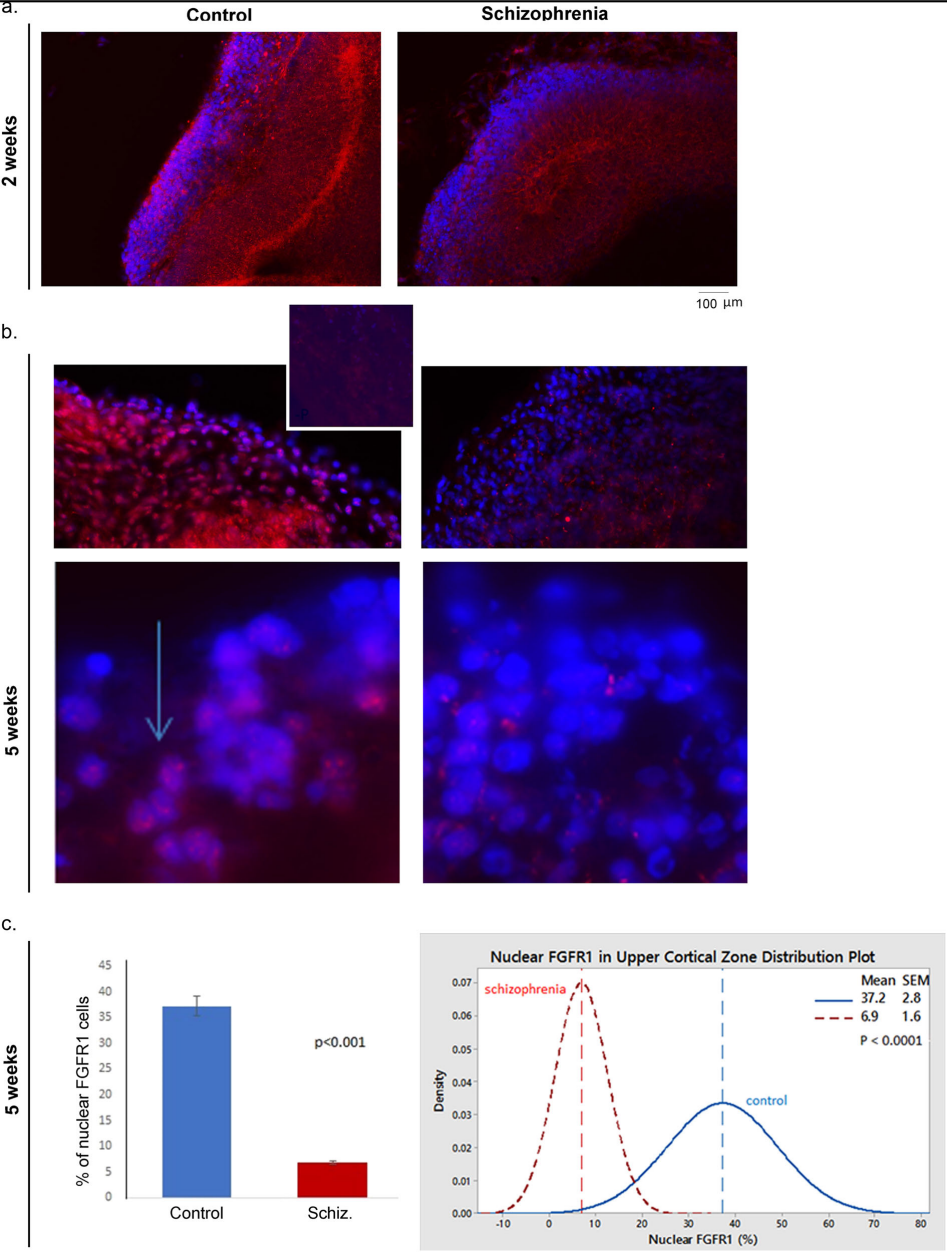
High expression of nuclear (n)FGFR1 in subcortical cells and the loss of nFGFR1 in cortical cells of schizophrenia organoids. **a** 2-week organoids: control (iPSC line BJ1) and schizophrenia (iPSC line 1835). Schizophrenia organoids have high FGFR1 expressing cells in VZ and dispersed in IZ. Few nFGFR1+ cells are present in CZ of the schizophrenia organoids. Images of whole sections are shown in Supplementary Fig. 4, a4 and a5. **b** 5-week organoids—control (BJ1) organoids express nFGFR1 in CZ and IZ (inset shows negative control—omitted primary FGFR1 antibody), and schizophrenia (1835) organoids show depletion of FGFR1 immunostaining in CZ. Arrow points to nuclei with FGFR1 speckles. 3D rotational confocal images of control (line 3651) and schizophrenia (line 1835) organoids are shown in Video 2a and b. **c** Quantification of the % of DAPI-stained nuclei that were immunopositive for nFGFR1 in multiple randomly selected ROI (3 × 10^3^ μm^2^, ∼40 cells/ ROI) in the upper CZ. The % of nFGFR1+ DAPI-stained nuclei was determined for multiple ROIs from the three control individuals and three patients. The difference between control and schizophrenia mean values was significant (*t*-test). Plots show distribution of the % of nFGFR1 positive nuclei in individual control (18 ROIs) and schizophrenia (12 ROIs). The individual value plots are shown in Supplementary Fig. 5d

Given the role of cytoplasmic FGFR1 in cell proliferation and nFGFR1 in neuronal differentiation and development, we analyzed the protein expression of FGFR1 in 2 and in 5-week-old iPSC organoids (Fig. 5). At 2 weeks, strong FGFR1 expression and high density of FGFR1+ cells, were detected in the VZ of controls, as well as schizophrenia iPSC organoids (Fig. 5a, Supplementary Fig. 4, a4 and a5). Both control and schizophrenia organoids displayed a less dense population of FGFR1 expressing cells in the IZ. The CZ cells of control organoids expressed FGFR1 (Fig. 5a), with many cells predominantly expressing FGFR1 associated with prominent nuclear speckles (Supplementary Fig. 4, a2). These speckles were previously shown to represent sites of RNA co-transcriptional processing and nFGFR1 interaction with the common transcriptional co-activator, CREB-binding protein (CBP)^43^.

In schizophrenia organoids, FGFR1 expression in the CZ was greatly reduced, as compared to control iPSC organoids at 2 weeks (Fig. 5a; Supplementary Fig. 4, a4 and a5), as well as at 5 weeks (Fig. 5b, Supplementary Fig. 4b and c). Few or no FGFR1-expressing cells were detected in the cortex of the schizophrenia organoids in investigated iPSC lines. In three patients and three controls, using multiple sections and ROIs, we counted the percent of DAPI-stained nuclei that were positive for nFGFR1. The number of nFGFR1-expressing cells was reduced from 37% in control organoids to 6.9% in the schizophrenia organoids (*p* < 0.0001) (Fig. 5c; Supplementary Fig. 5d).

Supplementary Videos 2a and b further illustrate the expression of FGFR1 in subcortical cells and the loss of FGFR1 in cortical cells of schizophrenia organoids.

Thus, the maldevelopment of the cortex in schizophrenia iPSC organoids was accompanied by a depletion of FGFR1 in the cortical cells.

### Loss of nFGFR1 signaling affects developmental genome programing

Specifically blocking nFGFR1 signaling with a nuclear dominant negative FGFR1(SP-/NLS)(TK-) in hESCs or in human or mouse NPCs blocked their neuronal differentiation and development. On the other hand, transfection of a nuclear constitutively active FGFR1(SP-/NLS) was sufficient to induce neuron development, sometimes producing unusually large neurons^28,52^. Together these loss and gain of function experiments demonstrated the importance of nFGFR1 homeostasis in neuronal development. Similar observations, albeit limited by the efficiency of in vivo transfection, were made upon nFGFR1 and nuclear FGF2 transfections of mouse brain stem cells^53,54^.

We next inquired whether downregulation of nFGFR1 in differentiating cortical neurons (NCCs) and its robust expression in subcortical NPCs (Fig. 5, Supplementary Fig. 4; overexpression in schizophrenia iPSC-derived NPCs was shown in ref. 55) could affect neuro-ontogenic gene activities. Toward this goal, we analyzed the transcriptomes of homogenous 2D cultures of NPCs derived from H9 hESCs and of their NCC progeny. NCCs were induced by treatment (48 h) of NPCs with cAMP, BDNF, and GDNF^29^ (see Supplementary Materials and Methods). Loss of nFGFR1 function was instated by transfection of the dominant negative nFGFR1(SP-/NLS)(TK-). Twenty-four hours after transfection, cells were maintained in NPC medium or treated with the differentiating factors, AMP/BDNF/GDNF, for an additional 48 h. To model excessive nFGFR1 signaling, NPC cultures were transfected with constitutively active nFGFR1(SP-/NLS) and treated with AMP/BDNF/GDNF. Parallel controls were transfected with a β-galactosidase-expressing plasmid.

Biological triplicates of each condition were used to isolate RNA and were analyzed independently by RNA-seq using an Illumina HiSeq2500 instrument. The raw data were analyzed by the Tuxedo pipeline and aligned to the UCSC genome hg38build. The expression levels of over 24,331 genes were assessed and considered significant (16,137 genes) when expressed in all samples (Supplementary Table S2). The remaining 8194 genes were not detected in all samples and were eliminated from further analysis due to their near-threshold expression. A total of 4704 genes showed expression levels that differed significantly (FC ≥ ± 1.5 and *q-*value > 0.05) between nondifferentiated NPCs and their NCC progeny induced by differentiating factors (cAMP/BDNF/GDNF) (Supplementary Table S3). Three hundred and thirty-two genes were differentially expressed between NPCs and NPCs+ FGFR1(SP-/NLS(TK-) (Supplementary Table S4). Expression of 861 genes differed significantly in NCCs pre-transfected with FGFR1(SP-/NLS)(TK-), as compared to β-galactosidase (Supplementary Table S5). In NCCs, 440 genes were affected by transfection of active nFGFR1(SP-/NLS).

Gene ontology (GO) analysis revealed many developmental categories overrepresented by the genes that were differentially expressed in nondifferentiated NPCs and NCCs (Table 2). The overrepresented categories included development of the nervous system, of the brain and its parts, cell pluripotency, proliferation, neuronal differentiation, axonal guidance and growth, synapse formation, glial development, and neuronal apoptosis. The same categories, with the exception of glial development, were overrepresented by genes affected by FGFR1(SP-/NLS)(TK-) in NPCs and/or NCCs. Thus, nFGFR1 appears to specifically control neuronal NPC development. Overexpression of active nFGFR1(SP-/NLS), along with the cAMP/BDNF/GDNF stimulation, affected genes of brain development, cell proliferation, neurogenesis, neuronal differentiation, and apoptosis. Thus, both insufficient and excessive nFGFR1 signaling disrupt neuro-ontogenic gene programs.

**Table 2:** Gene ontology analysis shows genes regulated between NPCs vs. NCCs, and genes affected by dominant negative nuclear FGFR1(SP-/NLS)TK-) or constitutively active nuclear FGFR1(SP-/NLS) (RNAseq)

NPC vs. NCC	NPC vs. NPC+ FGFR1(SP-/NLS)(TK-)	NCC+ vs. NCC+ FGFR1(SP-/NLS)(TK-)	NCC vs. NCC+ FGFR1(SP-/NLS)
Neural development			
Synapse development, assembly		Yes	
Synaptic plasticity			
NS/CNS development	Yes	Yes	
Brain parts development	Yes	Yes	Yes
Forebrain development			Yes
Cell proliferation, mitotic cycle	Yes	Yes	Yes
Cell migration	Yes	Yes	
Neurogenesis	Yes	Yes	Yes
Cell adhesion		Yes	
Extracellular matrix, proteases	Yes	Yes	
Cell, neuronal differentiation	Yes	Yes	Yes
Axon development	Yes		
Axonal guidance		Yes	
Oligodendrocyte development			
Glial development			
Neurotransmission			
Neuronal projection	Yes		
Learning and Memory			
		Response to retinoic acid	
		2nd messenger signaling	
	Regulation of signal transduction	Regulation of signal transduction	
		Cell–cell signaling	
Apoptosis, neuron apoptosis	Yes	Yes	Yes
Development general			
Organ and developmental morphogenesis	Yes		Yes
Angiogenesis	Yes		
Cardiovascular development	Yes		
Multicellular organism development	Yes	Yes	Yes
Cellular response to TGFβ			
Limb, organ morphogenesis	Yes	Yes	
Eye development	Yes	Yes	
		Developmental induction	
Gene expression			
	Transcription RNA PolII		Transcription RNA PolII

Categories affected only in FGFR1(SP-/NLS)(TK-) or FGFR1(SP-/NLS) transfected cells are listed by names

The ontological gene categories affected by cAMP/BDNF/GDNF, FGFR1(SP-/NLS)(TK-) and/or FGFR1(SP-/NLS) included also general multicellular organism development, morphogenesis, organ, cardiovascular, and limb development, cell–cell signaling, retinoic acid, and 2nd messenger signal transduction (Table 2). These findings match the widespread gene targeting by nFGFR1^19,29^ and its proposed pan-ontogenic function^20^. Also, consistent with the established transcriptional functions of nFGFR1^18^, genes which were affected by FGFR1(SP-NLS)(TK-) and FGFR1(SP-/NLS) overrepresented the functional category of RNA PolII-mediated transcription (Table 2).

The ingenuity pathway analysis (IPA) (a proprietary curation of pathways by Qiagen that estimates gene overrepresentation within specific pathways), revealed FGFR1(SP-/NLS)(TK-) dysregulation of many genes in NPCs and/or NCCs involved in pathways controlling neuronal and brain development, which were regulated during the cAMP/BDNF/GDNF-induced NCC differentiation (Table 3). These pathways included Wnt/β catenin signaling, axonal growth and guidance, ephrin, G-protein signaling, cAMP signaling, PKA signaling, CREB signaling, ERB tyrosine kinase signaling, growth hormone receptor signaling, interleukin signaling, prolactin, etc. (Table 3). In addition, inhibition with FGFR1(SP-/NLS)(TK-) revealed nFGFR1 regulation of additional developmental pathways, which were not affected by cAMP/BDNF/GDNF: Notch, Wnt/Ca++, neurotrophins/TRK, ERK/MAPK, reelin, ephrin, glucocorticoid receptors, GDNF and PDGF, as well as cell cycle and p53 controlling pathways. Overexpression of constitutively active nFGFR1(SP-/NLS) in NCCs affected genes in Notch, Ca++, GH, EGFR, CXCR4, pluripotency, and glucocorticoid signaling, neurotrophin/Trk receptors, NGF, Erk/MAPK signaling, ephrin signaling, FGF signaling, p53, and RAR signaling. Blocking nFGFR1 signaling with FGFR1(SP-/NLS)(TK-) and/or activation with FGFR1(SP-/NLS) affected genes in pathways involved in neuronal functions, such as GABA receptor signaling, calcium, melatonin, LTP, and LTD (Table 3). Thus, loss of nFGFR1 and excessive nFGFR1 signaling lead directly to dysregulation of major developmental pathways.

**Table 3:** Ingenuity pathway analysis (IPA)—selected IPA identified pathways that were regulated during cAMP/BDNF/GDNF-induced neuronal programming (NPC vs. NCC) and genes affected by dominant negative nuclear FGFR1(SP-/NLS)TK-) or constitutively active nuclear FGFR1(SP-/NLS) (RNAseq)

NPC vs. NCC	NPC vs. NPC+ FGFR1(SP-/NLS) (TK-)	NCC vs. NCC+ FGFR1(SP-/NLS) (TK-)	NCC vs. NCC+ FGFR1(SP-/NLS)
Wnt/β-catenin	Yes	Yes	
cAMP signaling	Yes		
Axonal guidance signaling		Yes	
G-protein signaling	Yes	Yes	
PKA signaling	Yes	Yes	
Neuropathic pain signaling		Yes	Yes
Corticotropin releasing hormone signaling		Yes	
Prolactin signaling	Yes	Yes	Yes
Glioma signaling		Yes	
GABA receptors			Yes
Nos in neurons	Yes		
VEGF		Yes	
Synaptic LT Depression		Yes	
Synaptic LT Potentiation		Yes	Yes
P2Y purinergic signaling	Yes	Yes	Yes
aldosterone	Yes	Yes	
Calcium signaling	Yes		Yes
HIF1A	Yes	Yes	
Growth Hormone signaling		Yes	Yes
IL-8		IL-8	
Clathrin endocytosis	Yes	Yes	Yes
Catecholamine biosynthesis			
ERB	Yes	Yes	EGFR
Melatonin signaling	Yes		
CXCR4			Yes
GAP junction signaling	Yes	Yes	
Ephrin receptor signaling		Yes	
ESC pluripotency	Yes	Yes	Yes
Enos	Yes	Yes	
TR/RXR		Yes	
IL8		Yes	
citruline		Yes	
Macrophages, fibroblasts inflamation		Yes	Yes
Renin/Angiotensin	Yes	Yes	
Huntington Disease signaling	Yes	Yes	
endothelin		Endothelin	
CREB	CREB	Yes	
Catecholamine biosynthesis			
	Notch		Notch
	Wnt/Ca		
	Agrin		
	Neurotrophin/TRK	Yes	
	Telomerase		
	Cyclin cell cycle		
	ERK/MAPkinase	Yes	
	AMPK signaling	Yes	
	Glucocorticoid	Yes	Yes
	P53	Yes	
	FGF signaling	Yes	Yes
	GDNF	Yes	
		EGF signaling	Yes
	Actin cytoskeleton	Yes	
	Jak1,3		
	IGF		
		Glioma invasiveness	Yes
			IL-6,17a
		HMGB	Yes
		LPS-MAPK	Yes
		PDGF	Yes
		RAR signaling	RAR activation
	DNA repair		
	Acute myeloid leukemia signaling		Yes
			CCK/Gastrin
		EMT transition	
		NF-kB	
		nanog	
		II-2,4,3,8,9,15,12,	
		Wnt/GSK-3Beta	
		NGF	
		Reelin in neurons	
		Melanocyte development	
		Myc-apoptosis	
		STAT3	
		PKC	
	Epithelial adherence junction		

Categories affected only in FGFR1(SP-/NLS)(TK-) or FGFR1(SP-/NLS) transfected cells are listed by names

Graphs of selected nFGFR1-dependent pathways and their affected genes are shown in Supplementary Fig. 6.

### Deconstruction of mRNA networks by loss of nFGFR1 signaling

Genome function is organized into highly coordinated and dynamically changing networks of genes. To detect such networks among the FGFR1-controlled genes, we performed a pairwise correlation^29^ of all differentially regulated genes. Among 4704 genes that were regulated during NPC differentiation to NCCs, we detected groups of genes that displayed high-positive correlation (+0.9 to +1.0; changing activities in the same direction) and genes which showed high-negative correlation (−0.9 to −1.0; changing activities in opposite direction) (Supplementary Fig. 7a). In each category, the 200 most connected genes from the differentiated NCCs were analyzed using circular network graphs. A common feature of both positive and negative networks was that their top 200 coordinate genes were highly interconnected in NCCs (Supplementary Fig. 7b, c). These networks formed during NCC differentiation as they showed little or no connectivity in NPCs.

**Fig. 6:**
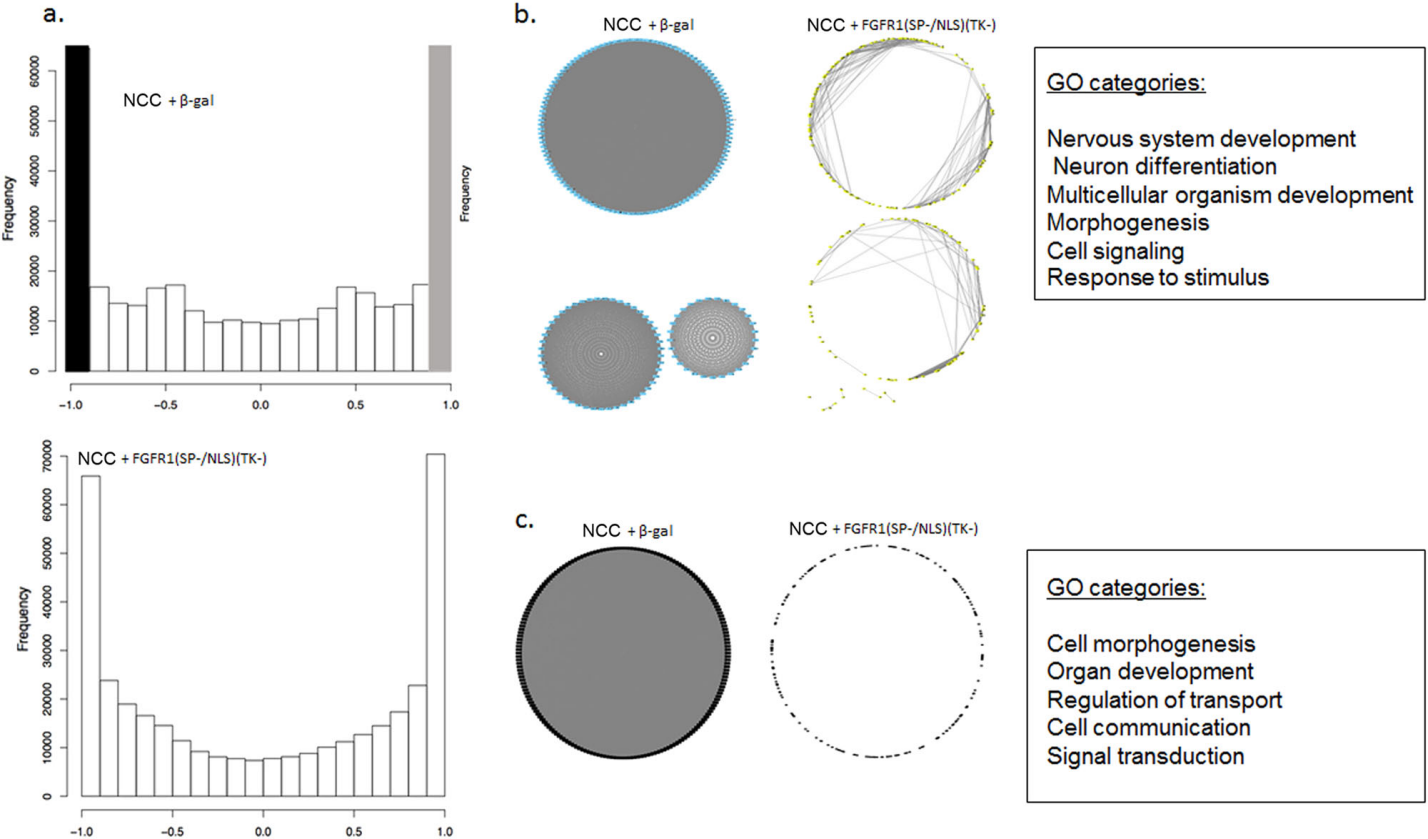
**(a)** Histogram of pairwise mRNA correlations Correlation was performed using three controls and three patients and triplicate cell samples. NPCs were transfected with control DNA or FGFR1(SP-/NLS)(TK-) and 24 h later were stimulated for 48 h with neuronal differentiation inducing media with cAMP/BDNF/GDNF (NCCs). Genes (861), which were affected by dominant negative nuclear FGFR1(SP-/NLS)(TK-) were analyzed. Genes that showed the highest positive (+0.9 to +1.0) correlations (changing in the same direction) are represented by the gray bar. Genes that showed the highest negative (−0.9 to −1.0) correlations (changing in the opposite directions) are shown as a black bar. **b**, **c** Among the FGFR1(SP-/NLS)(TK-) regulated genes, top 200 of the positively correlated genes (**b**) and top 200, which were negatively correlated genes (**c**) were selected for the circular network analysis. Gray lines link pairs of genes whose correlation is greater than 0.9. In the control β-galactosidase set, three separate networks were formed. In the FGFR1(SP-/NLS)(TK-) transfected cells, two weakly correlated networks and few individual correlated genes are observed. GO categories overrepresented by 200 top connected genes are listed

**Fig. 7:**
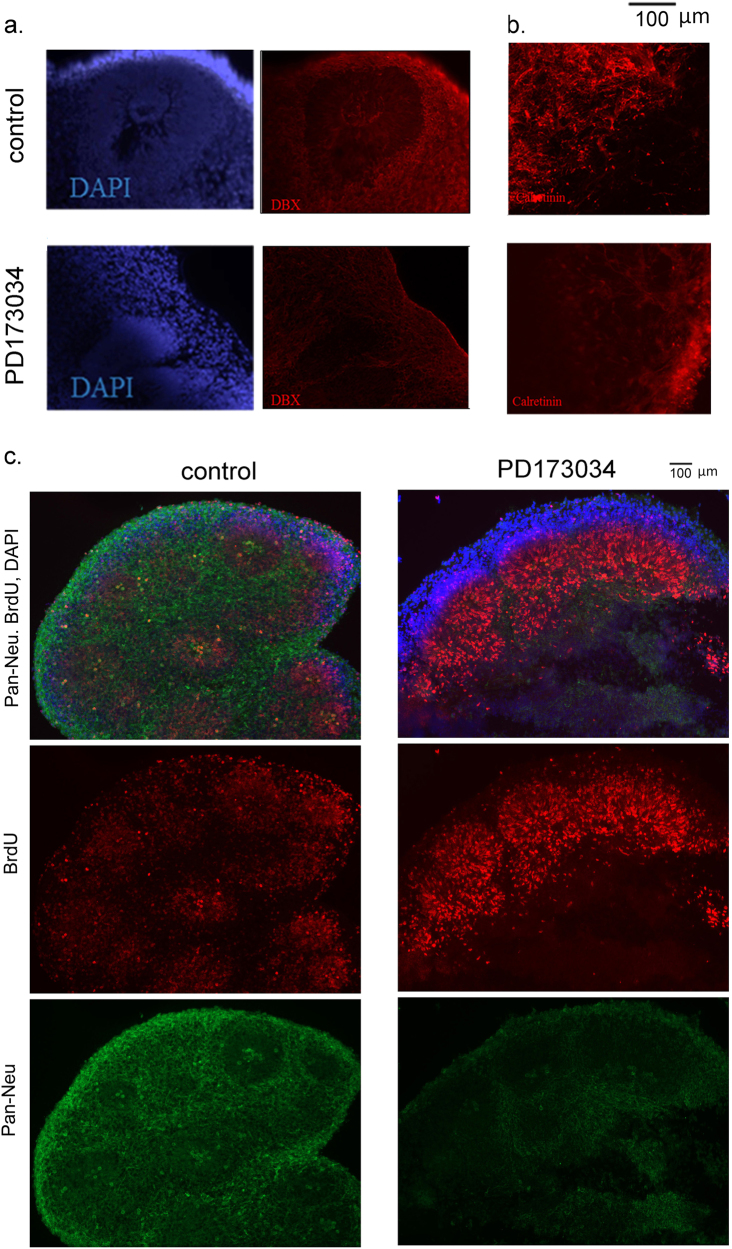
Treatment with PD173074 (days 8 and 18) affects cortical development in hESC H9 organoids **a** Double staining for DAPI and doublecortin (DBX, neuroblasts). **b** PD173074 reduces expression of calretinin in hESC organoids. **c** BrdU pulse-chase experiment. c—control hESC organoids, d—PD173074 treated (days 8 and 18) organoids. PD173074 inhibits cortical migration and neuronal differentiation of newborn cells in hESC organoids. Sections were coimmunostained for BrdU (red), Pan-Neu (green), and DAPI (blue). Merged and individual stains are shown. Inhibition of FGFR1 with PD173074 inhibits migration and formation of new BrdU+ cortical neurons in the CZ

We then analyzed network formation by 861 NCC genes, which were affected by FGFR1(SP-/NLS)(TK-) (Fig. 6). Significantly, the networks formed by the top 200 positively and 200 negatively correlated genes in control β-galactosidase transfected NCCs were deconstructed in the NCCs transfected with FGFR1(SP-/NLS)(TK-) (Fig. 7). Genes forming these nFGFR1-dependent positive and negative correlative networks represented several of the neuro-ontogenic categories (Fig. 6).

### FGFR inhibitor, PD173074, models loss of nFGFR1 and cortical maldevelopment in hESC cerebral organoids

Given the broad neuro-ontogenic functions of nFGFR1 in NPCs and NCCs, we next asked if the loss of nFGFR1 function could arrest the ongoing cortical development in control hESC organoids, similar to as observed in cortical cells of the schizophrenia organoids. We used PD173074, which specifically inhibits FGFRs (R1 > R2 > R3 > R4)^56^. Since *fgfr1* was the highest expressed *fgfr* gene in NPCs and NCCs (Supplementary Table 2; 1.0_FGFR1_ > 0.16_FGFR2_ > 0.26_FGFR3_ > 0.008_FGFR4_), the effects of PD173074 would likely reflect the FGFR1 blockade. After the initial 8 days of cortical development, structurally stable hESC cerebral organoids were incubated with 100 nM PD173074 or in drug-free medium for an additional 10 days, during the ongoing CZ expansion.

In general, many changes induced by PD173074 treated hESC H9 organoids were similar as in the schizophrenia iPSC organoids. Expression of the cortical TBR1 marker of early pioneer neuroblasts, was abolished by PD173074 (Supplementary Fig. 8a, b). Consistent with these findings, the highly abundant doublecortin+ neuroblasts (Fig. 7a) and the calretinin+ interneurons (Fig. 7b) in the IZ and CZ of day 18 control organoids were markedly depleted by the PD173074 treatment.

To determine if FGFR1 inhibition affected the ongoing cortical neuronogenesis, we pulse labeled dividing cells with BrdU in 14-day organoids (6th day of PD173074 treatment). After an additional 4 days, BrdU-labeled control or PD173074-treated organoids were sectioned and co-immunostained with rat anti-BrdU and with mouse anti-Pan-Neu. In control organoids, the BrdU-labeled cells were found in the VZ, as well as migrated into the IZ and CZ (Fig. 7c). Counting cells in multiple rosettes showed BrdU+ cells abundantly present in all zones of the control organoids in the following order: VZ > IZ > CZ. In contrast, in PD173074-treated organoids, BrdU+ cells were most abundant in the IZ with only few cells present in the cortex (IZ > VZ > CZ) (Fig. 7c and Table 4).

**Table 4:** Effect of PD173074 on distribution of BrdU+ cells and double labeled BrdU+ plus Pan-Neu+ cells in rosettes and in the cortical zone

	Control	PD173074
Rosette ROIs	BrdU	BrdU
	47.7 ± 3.4 (77%)	136.2 ± 22.7 (98%)
		*P* = 0.006
	BrdU + Pan-Neu	BrdU + Pan-Neu
	18.3 ± 1.7 (74%)	9.2 ± 2.6 (93%)
		*P * = 0.008
Cortex ROIs	BrdU	BrdU
	14.2 ± 2.1 (23%)	2.5 ± 0.9 (2%)
		*P* = 0.0007
	BrdU + Pan-Neu	BrdU + Pan-Neu
	6.5 ± 1.2 (26%)	0.7 ± 0.3 (7%)
		*P* = 0.002

Cells were counted in identical seven ROIs in sections of control and PD173074-treated organoids. Numbers represent mean + SEM; % of cells in rosettes and cortical ROI are shown. Pairwise comparisons were done using *t*-test with unequal variances

In control organoids, BrdU and Pan-Neu colocalized predominantly in the same cells of the marginal CZ (Fig. 7c, Supplementary Fig. 9a), indicating that the dividing VZ cells had migrated out to cortical layers and differentiated into neurons. Treatment with PD173074 markedly altered this process (Fig. 7c, Supplementary Fig. 9b). The BrdU+ cells remained largely arrested in the IZ. Relatively few BrdU+ cells that were found in the CZ differentiated to Pan-Neu positive neurons (Table 4). Thus, blocking FGFR1-inhibited cortical migration of the newborn cells and formation of mature neurons.

**Fig. 8:**
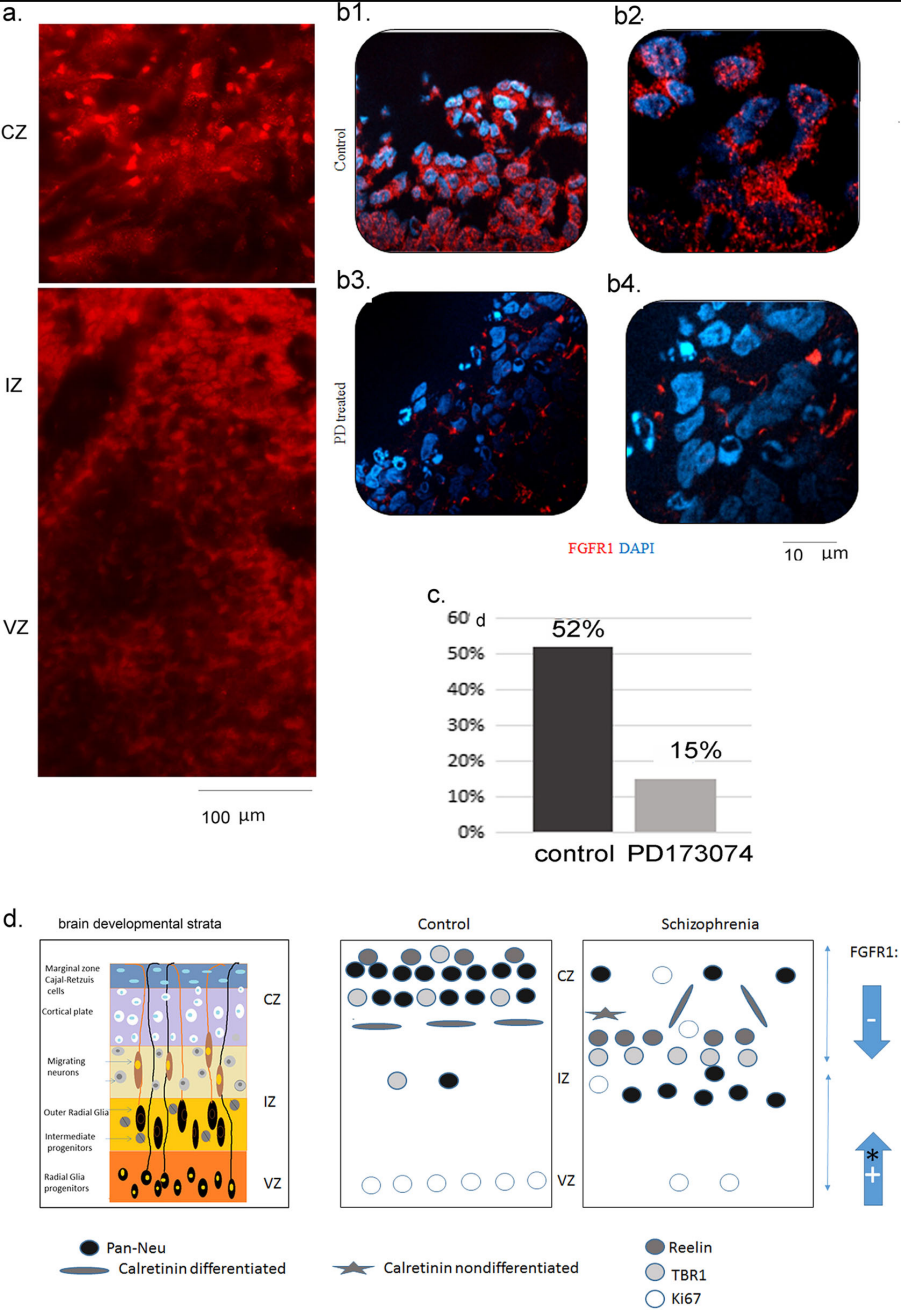
**a** Distribution of FGFR1 in zones of hESC H9 organoids (fluorescent microscope images). **b** Reduction of nFGFR1 in CZ after PD173074 treatment (days 8–18)—confocal analysis of FGFR1 and DAPI co-staining; (b1) control and (b2) PD173074-treated cerebral organoids. (b3) control and (b4) PD173074, areas zoomed 63×. **c** Percentage of nFGFR1 expressing cells in CZ was reduced by PD173074—nFGFR1+ nuclei were counted in sets of 100 DAPI-stained nuclei. **d** Left—reference stratification of developing telencephalon and zones of cerebral organoids—ventricular (VZ), intermediate (IZ), cortical (CZ), marginal (MZ); middle and right—summary of results—in schizophrenia organoids, we found the following changes: (i) increased proliferation of Ki67 NPCs and migration outside the VZ into the IZ and CZ, (ii) diminished deposition of reelin in the developing cortex (known to guide cortico-petal migration), (iii) reduced cortical accumulation of pioneer TBR1 neurons and reduced formation of cortical neurons, (iv) stunted cortical neuronal development accompanied by a robust formation of the subcortical neurons, and (v) fewer calretinin interneurons forming horizontal processes (known to connect cortical columns). The premature development of NPCs into subcortical neurons may reflect excessive nFGFR1 (+)* signaling in differentiating schizophrenia NPCs (as found in earlier genomic studies^55^). On the other hand, stunted cortical development likely reflects the loss of cortical nFGFR1 signaling. Modeling this loss in hESC organoids, by blocking FGFR1 signaling and depleting nFGFR1 with PD173074, replicates the impaired cortical development observed in schizophrenia iPSC organoids. The loss of cortical nnFGFR1 may underlie the stunted cortical development in schizophrenia

PD173074 was shown previously to reduce nuclear nFGFR1 accumulation observed in pancreatic tumors^57^. Hence, we examined the PD173074 effect on FGFR1 subcellular distribution in the CZ of hESC H9 cerebral organoids. In several independent experiments, the expression of FGFR1 in control hESC H9 organoids showed similar patterns (Fig. 8a) as in the control iPSC organoids (Fig. 5a). FGFR1 was highly expressed in the VZ cells (Fig. 8a). The FGFR1 staining was less intense in the IZ and stronger again in the CZ. In many CZ cells, FGFR1 localized within the cell nuclei (Fig. 8a) and in prominent nuclear speckles (Fig. 8, b1). To quantify the changes in FGFR1 subcellular distribution, we imaged DAPI and FGFR1 co-stained sections using confocal microscopy (Fig. 8b). In the CZ of control organoids, 52% of cells had nFGFR1 colocalized with DAPI. In PD173074-treated organoids there was over a three-fold reduction in the number of cells with nFGFR1 in the CZ (Fig. 8c), likely reflecting loss of the positive *fgfr1* gene activation by its protein^19,57^.

## Discussion

In this study, we have expanded cerebral organoids as an experimental platform to analyze the early human cortical development in healthy and disease states and to investigate disease mechanisms. Using the protocol of Lancaster et al.,^40^ in which the first step, an efficient generation of EBs, was modified as in Brennand et al.,^33^ we successfully generated cerebral organoids from hESCs (H9 and HUES8 lines) and from seven human iPSC lines. We observed time-dependent generation and development of organoids, recapitulating the inside-out pattern of human cortical development. The VZ in the center of the rosettes contained proliferating GFAP+ radial glia, the IZ contained migrating doublecortin+ neuroblasts and immature βIII-tubulin+ neurons, and the CZ incorporated pioneer neurons (TBR1+), and built cortical-like layers of the βIII-tubulin+ and Pan-Neu+ neurons, and calretinin+ interneurons. We employed quantitative cell counting, image intensity measurements, and computational tools to quantify and analyze normal and pathological tissue samples.

An important observation made repeatedly during this study was that the cellular structures and zones of the organoid rosettes and their development were consistently reproducible among hESC H9, HUES8, and the four control iPSC line organoids, but were noticeably disturbed in all schizophrenia iPSC organoids and after pharmacological inhibition of FGFR1. In the organoids developed from either hESCs or control iPSCs, the proliferating cells were restricted to 2–3 layers surrounding the lumen of the VZ rosettes. Relatively few cells that migrated to the IZ remained in a proliferative state, but essentially, no such cells were found in the CZ. One of the most striking features of the schizophrenia iPSC organoids was the disruption of the developmental strata. Specifically, we observed significantly increased proliferation and movement of Ki67+ NPCs into the IZ, and development of atypically placed deep subcortical neurons. These changes were accompanied by significant loss of the TBR1 pioneer neurons from the top cortical layers, while they remained abundant deep in the IZ. Together, these findings illuminated an abnormal subcortical neuronogenesis in schizophrenia tissue and were consistent with the increased, premature neuronal generation, predicted from the transcriptome studies of the NPCs derived from schizophrenia iPSCs^29^.

The expanded subcortical neurogenesis was accompanied by opposite changes in the CZ, where fewer mature neurons developed in schizophrenia organoids. This was shown by a significant reduction in cortical Pan-Neu intensity and was corroborated by visibly diminished Pan-Neu fibers in the schizophrenia organoids. The specific depletion of the neurons in the cortex, but not in the IZ, may reflect the loss of cortical signals that guide the cortico-petal migration, i.e. reduced cortical deposition of reelin and pioneer TBR1 neurons. The altered intracortical connectivity in schizophrenia organoids was further denoted by the changes in the orientation of the calretinin+ interneurons. Unlike the horizontal organization of calretinin+ neurites in the control cortex, the schizophrenia interneurons displayed a random directionality of neurites, indicating perturbed intercortical connectivity. The relationship between glutamatergic pyramidal neurons and GABAergic interneurons (of which calretinin-positive neurons are a subtype of GABAergic interneurons), has been suggested in relation to the development of schizophrenia in humans^58^. So far, attempts to count calretinin neurons in the schizophrenia brain showed lack of significant numerical changes^59^. Our study suggests that changes in interneuron directionality may disrupt cortical connections and communication in schizophrenia.

In summary (Fig. 8d), the impaired development of cortical neurons, interneurons, and cortical connections in schizophrenia may be instigated by reduced cortico-petal migration of the neuroblasts and immature neurons. In addition, the disruption of cortical neuronal programing in schizophrenia organoids coincided with the presence of the Ki67+ proliferating cells in the CZ, suggesting that these cells have failed to exit the cell cycle and differentiate.

### Role of FGFR1

The impaired cortical development of schizophrenia organoids correlated with a striking loss of nFGFR1 from the CZ cells observed already at 2 weeks and was verified by a statistically significant loss in 5-week organoids of all investigated patients. The loss of nFGFR1 was limited to cortical cells, as FGFR1 remained highly expressed in the VZ and IZ of schizophrenia organoids throughout the 5 weeks of development. Given the well-established role of nFGFR1 in cell differentiation and neuronal development^18,20^ (-reviews), the loss of nFGFR1 from the nuclei of differentiating cortical neuronal cells in schizophrenia organoids likely underlies the impaired neuronal differentiation and cortical maldevelopment observed in schizophrenia. Furthermore, blocking of nFGFR1 in NPCs and NCCs impacted a number of the key ontogenic and neuro-ontogenic gene categories involved in neuronal development, forebrain development, cell adhesion and migration, axon formation, and guidance and in the established neuro-ontogenic pathways. Our genomic analyses also revealed that the loss of nFGFR1 function in NPCs affected several of the reelin signaling genes (Supplementary Fig. 6c), which together with the loss of reelin, denotes the malfunctioning reelin mechanism in the schizophrenia cortex.

The importance of these genomic findings transcends the current investigation centered on schizophrenia. Demonstration that the developmental gene programs known to be targeted directly by nFGFR1^19,29^ are indeed regulated by the nuclear receptor, and further substantiates the panontogenic-INFS theory^20^.

In addition to investigating the loss of nFGFR1 function, our experiments showed that overactive nFGFR1 also affects the neuro-ontogenic programs. Excessive nFGFR1 gene targeting was shown in schizophrenia NPCs during their differentiation^29^. Thus, an over-activation by nFGFR1 of neurodevelopment gene programs in subcortical NPCs could lead to their premature migration and differentiation observed in the subcortical regions of the schizophrenia organoids.

Within the list of the nFGFR1-regulated genes (Supplementary Table 7), ∼75% were previously shown to bind to nFGFR1 in control and/or schizophrenia iPSC-derived differentiating NCCs^29^. Thus, the effects of transfected dominant negative and constitutively active nuclear forms of FGFR1 predominantly reflect direct gene control by the nuclear receptor.

As nFGFR1 has been designated as a central neuro-ontogenic factor^19,20^, we propose that the depletion of cortical nFGFR1 in schizophrenia organoids leads to dysregulation of the key neuro-ontogenic programs and, consequently, arrests cortical neuronal development. Indeed, the inhibition of FGFR1 signaling (the predominant FGFR expressed) with PD173074, depleted nFGFR1 in the CZ of control hESC organoids, and inhibited cortical development, similar to as observed in the schizophrenia organoids. The BrdU pulse-chase experiment showed that blocking FGFR1 reduced cortical migration of new born cells and their differentiation to neurons. Furthermore, PD173074 exposes the role of FGFR1 in the formation of doublecortin expressing neuroblasts and TBR1 expressing neuroblasts/pioneer neurons. It also demonstrated the importance of FGFR1 signaling in the development of calretinin interneurons.

The early cortical malformations revealed by this organoid study could underlie the disruption of cortical layers, cell paucity, hypoplasia, abnormal connections, and white matter tracks observed in adult schizophrenia brains by pathological examination and/or in vivo imaging^17,60^.

Why do schizophrenia symptoms appear in adolescence or young adulthood if they are a consequence of early prenatal brain maldevelopment? In schizophrenia patients, the malformations are often found in the prefrontal cortex^60,61^, in which myelination and maturation occur at the time when the first severe symptoms emerge. As the prefrontal cortex becomes increasingly incorporated into the human brain circuitry, its developmental malformations may reveal itself in a complex clinical syndrome. Perhaps, the most severe forms of childhood schizophrenia, with abundant hallucinations and motor dysfunctions^62^, arise also in the cortical regions, which mature earlier and are home to the sensory and motor functions^63^. These and other compelling questions await further investigation.

In the present study, we observed similar anatomical changes in the organoids of all three patients that have distinct genetic backgrounds and DNA sequences, which could be linked to schizophrenia. However, the iPSC-derived NPCs all showed a common disruption of 1376 genes in the developmental transcriptome^29^. Thus, our studies reinforce the watershed model of schizophrenia, whereby different mutations lead to the common dysregulation of the neuro-ontogenic genome and to the common cortical maldevelopment. Our findings designate INFS as a common important mechanism in the disease. According to the model shown in Fig. 8d, nFGFR1 hyperactivity leads initially to the excessive migration of proliferating NPCs and then their premature differentiation within subcortical regions. Later, during corticogenesis, turning off nFGFR1 in the schizophrenia cortical cells disrupts their further development. Future investigation, including restoration of INFS in the developing schizophrenia cortex, will test this hypothesis and potentially be used in preventive therapies.

## Electronic supplementary material

Supplementary Materials Methods Computation Figures

Supplementary Tables 2-7

Video 1a

Video 1b

Video 2a

Video 2b
