# Supplementary material for: Cerebral organoids reveal early cortical maldevelopment in schizophrenia—computational anatomy and genomics, role of FGFR1
Source: Transl Psychiatry. 2017 Nov 17;7:6. doi: 10.1038/s41398-017-0054-x (PMC5802550; doi:10.1038/s41398-017-0054-x)
Supplement: Supplementary file 1 — Supplementary Materials Methods Computation Figures [file 41398_2017_54_MOESM1_ESM.pdf]

# Supplementary Materials

**Cerebral organoids reveal early cortical maldevelopment in schizophrenia – computational anatomy and genomics, role of FGFR1.**

E.K. Stachowiak, C.A. Benson, S.T. Narla, A. Dimitri, L. Bayona Chuye, S. Dhiman, K. Harikrishnan, S. Elahi, D. Freedman, K.J. Brennand , P. Sarder, M.K. Stachowiak

# Contents


1.0 Supplementary Methods: ..... 3

1.1 Cell cultures ..... 3

1.2 Cerebral Organoids ..... 3

1.3 Immunocytochemical Staining ..... 4

1.4 Microscopy ..... 4

1.5 Cultures and transfections of hESC H9 derived neural progenitor cells (NPC) and neuronal committed cells (NCCs). ..... 4

1.6 Plasmids expressing FGFR1 constructs ..... 4

1.7 RNAseq and data analysis ..... 5

1.8 Quantitative and computational analyses of histological images ..... 5

2.0 Computational Methodology FOR image analysis. ..... 6

2.1 Cell Segmentation (Marker-Controlled Watershed) ..... 6

2.1.1 Catchment Basin Identification ..... 7

2.1.2 Watershed Segmentation ..... 8

2.1.3 Cell Segmentation Overview for Ki67 and Calretinin Images ..... 9

2.2 Euclidean Minimum Spanning Tree (Ki67) ..... 14

2.2.1 Delaunay Triangulation ..... 14

2.2.2 Prim's Algorithm for EMST ..... 15

2.3 Deviation Angle and Major Axis Length (Calretinin) ..... 16

2.4 Data Matrices ..... 18

3.0 Supplementary Figures: ..... 21

4.0 Supplementary Videos Legend ..... 37

5.0 Supplementary Tables: ..... 38

# 1.0 SUPPLEMENTARY METHODS:

## 1.1 Cell cultures

hESC line, H9 (female karyotype) from WiCell Research Institute (Madison, WI), and HUES8 iCas9N (male karyotype; gift from Dr. Danwei Huangfu, Memorial Sloan Kettering Cancer Center) were used under the approval of the Committee for Stem Cell Research Oversight at SUNY Buffalo. hESCs were differentiated into NPCs utilizing the same protocol as for iPSCs. In brief, hESCs were transferred to a non-adherent plate (Corning). Embryoid bodies (EBs) were grown in suspension overnight in N2/B27 medium (DMEM/F12 plus GlutaMAX (ThermoFisher Scientific), 1X N2 (ThermoFisher Scientific), 1X B27 without Vitamin A (ThermoFisher Scientific), and 1X pencillin/streptomycin). For 6 days, the EBs were fed with N2/B27 medium supplemented with dual SMAD inhibitors, 0.1 μM LDN193189 (Stemgent) and 10 μM SB431542 (Tocris). EBs were plated in N2/B27 medium supplemented with dual SMAD inhibitors and 1 μg $ml^{-1}$ laminin (Sigma Aldrich) onto polyornithine (PORN)/laminin-coated plates. Visible rosettes formed within one week and were detached using Neural Rosette Selection reagent (Stem Cell Technologies). Rosettes were cultured in NPC medium (DMEM/F12 plus GlutaMAX, 1X N2 Supplement-B (Stem Cell Technologies), 1X NeuroCult SM1 without Vitamin A (B27) (Stem Cell Technologies), 1 μg $ml^{-1}$ laminin, 20 ng $ml^{-1}$ FGF2 (ThermoFisher Scientific), and 1X penicillin/streptomycin). NPCs were then transferred from polyornithine/laminin-coated plates onto matrigel-coated plates and were maintained in NPC media.

## 1.2 Cerebral Organoids

H9 hESCs - cells were maintained on matrigel (Corning) according to WiCell's feeder-free protocol utilizing mTeSR medium and matrigel coated plates. hESCs were incubated with collagenase (1mg/ ml in DMEM-F12) for 1-2 hours. Lifted colonies were washed with DMEM-F12 and resuspended in N2/B27 media and transferred to 6-well low attachment plates (Corning) The Embryoid Bodies (EBs) formed after 24 hours and were fed every day with N2/B27 media supplemented with dual SMAD inhibitors. After 4-5 days, the EBs were transferred one by one to a 24-well low attachment plate using a cut-pipette tip and incubated with neural induction medium. The neuroepithelial tissues were fed every other day for 4 days. Afterward, the neuroepithelial tissues were transferred to matrigel droplets on dimpled parafilm. The droplets were solidified at 37°C and the parafilm was removed subsequently. Neuroepithelia were grown stationary for 4 days in a differentiation media without vitamin A. Day 1 cerebral organoids were transferred to an orbital shaker and fed every 3-4 days with differentiation media with vitamin A. Organoids were harvested and processed as described for iPSCs.

iPSCs and HUES8 – Cells were maintained on gelatin coated plates with MEFs in hES medium (DMEM-F12 with GlutaMAX, 20% Knockout Serum Replacement (KOSR; ThermoFisher Scientific), 1X MEM-NEAA (ThermoFisher Scientific), 2-mercaptoethanol, 20 ng $ml^{-1}$ FGF2 (iPSCs) or 10 ng $ml^{-1}$ FGF2 (HUES8), and 1X penicillin/streptomycin)[1]. The generation of cerebral organoids using iPSCs is described in Supplementary Figure 1.

## 1.3 Immunocytochemical Staining

Organoid cryosections were rehydrated and permeabilized with 0.5% Triton-X-100 (15 minutes) and blocked with normal goat serum (NGS in 1X PBS, 1 hour at room temperature). Afterwards, sections were incubated overnight with primary antibody diluted in Antibody Diluent (IHC WORLD). The secondary antibody was applied for 1-2 hours at room temperature. For double immunostaining the sections were washed in 1X PBS and incubated with the second primary antibody overnight followed by the second secondary. The antibodies used are listed in the Supplementary Table 1. The control and experimental organoid sections were immunostained at the same time using identical protocols. Each staining was performed in triplicate. At least three organoids were examined for each experimental condition. The stainings produced similar results and representative images are shown.

The specificity of immunostaining was verified with control reactions, in which the primary antibody was omitted or replaced with preimmune sera producing no fluorescence signal, as described previously [2, 3]. The nuclear presence of FGFR1 was demonstrated in several laboratories in non-transformed cells [4, 5], cancer cell lines [2, 6-10], stem cells, and in the rat and mouse brain [11, 12 8, 13, 14] using an array of antibodies that target different FGFR1 epitopes. Furthermore, transfected FGFR1-EGFP in live cells was detected using native fluorescence and FGFR1-Flag using αFlag (REF). In the present study, as previously described, nuclear and cytoplasmic FGFR1 were labeled using Abcam ab10646 and Abcam FGFR1 Mab[15-17].

## 1.4 Microscopy

Organoid sections were analyzed using fluorescent Nikon Diaphot microscope or by confocal microscopy. Confocal sections were acquired with Biorad MRC 1024 confocal microscope (BioRad Laboratories, Hercules, CA, USA)[17]. The possibility of bleed-through in double fluorescent cells was excluded by acquiring images in sequential mode. In addition (Supplementary videos 1 and 2) Images were acquired on the Andor Dragonfly spinning disk confocal system mounted on a Leica DMI8 using a 40x 1.4NA Plan Apo objective lens with a Zyla 4.2 sCMOS camera. Excitation wavelengths are 405 and 561 and emission filters are 450/50 and 600/50. 3D volumes were acquired at an interval of 0.21 microns. Deconvolution was performed in Andor Fusion using a robust iterative Richardson-Lucy algorithm.

## 1.5 Cultures and transfections of hESC H9 derived neural progenitor cells (NPC) and neuronal committed cells (NCCs).

Human ESC line H9 was differentiated into NPCs as described previously[1, 18]. To initiate neuronal development, NPCs were cultured in neuronal differentiating media for 2 days (DMEM/F12, 1× N2, 1× B27-RA, 20 ng $ml^{-1}$ BDNF (Peprotech), 20 ng $ml^{-1}$ GDNF (Peprotech), 1 mM dibutyryl-cyclic AMP (Sigma), and 200 nM ascorbic acid (Sigma)[18]. After two days of treatment, the neuronal comitted cells (NCCs) were harvested for RNAseq.

## 1.6 Plasmids expressing FGFR1 constructs

FGFR1 (SP-/NLS)-signal peptide replaced with the nuclear localization signal (NLS) from the SV40 large T antigen, and FGFR1 (SP-/NLS)(TK-) were described in [7, 19]. All transfections were carried out using Fugene 6, per manufacturer's instructions. hESC-derived NPCs were transfected using 6 µg of either control β-gal, FGFR1(SP-/NLS) or FGFR1(SP-/NLS)(TK-) per well in a 6-

well plate. 24h after transfection, cells were maintained in NPC medium for an additional 48h (non-differentiated NPCs) or treated with BDNF/GDNF/dBcAMP to induce NCCs. Three wells were combined into a single sample and cells were harvested for RNAseq. Three samples were analyzed for each condition.

## 1.7 RNAseq and data analysis

RNAseq was performed and data analyzed as previously described [1, 17, 20]. RNA was extracted using Trizol or the Qiagen RNA extraction kit, according to manufacturer's instructions. RNA was prepared using the Tru-Seq RNA kit and purified library cDNA was captured on an Illumina flowcell for cluster generation and sequenced on an Illumina Hi-seq 2500, following the manufacturer's protocols. For independent mRNA assays, cDNA synthesis was carried out using 1 µg of RNA and the iScript cDNA Synthesis Kit (Bio-Rad; Hercules, CA). RNA-seq results were analyzed using the tuxedo pipeline and statistics [21]. Raw FASTQ reads were mapped to the Homo sapiens genome build hg19 (UCSC) using tophat 2. These mapped reads were assembled into transcripts and gene expression levels using Cufflinks version 2.0.2. Transcript assemblies were merged together using Cuffmerge and Cuffdiff was used to determine significant differences in gene expression (FPKM). Heatmaps of differentially expressed genes were generated using R. Data pertaining to RNAseq can be accessed on GEO with accession code: GSE103307.

## 1.8 Quantitative and computational analyses of histological images

Concentration of the extracellular protein, reelin, was evaluated by measuring fluorescence intensity in sections stained with Reelin Ab and Alexa Fluor® 488. High densities of the Pan-Neu stained neurons and fibers in schizophrenia and control iPSC organoids precluded their direct counting. Therefore, we compared their densities indirectly by measuring fluorescence intensity in sections stained with Pan-Neu Ab plus Alexa Fluor® 488. Fluorescent Intensity Measurements (FIM) were performed using Zen 2.0 Blue Imaging software in randomly selected ROIs within organoid regions listed in the Figure Legends. The analyses were performed in multiple sections of organoids developed from three control and three schizophrenia patients.

Counting of the BrdU labeled nuclei, FGFR1 or TBR1 expressing nuclei and DAPI positive cells was performed using Visiopharm Stereological software as described previously [17].

## 2.0 COMPUTATIONAL METHODOLOGY FOR IMAGE ANALYSIS.

Images, essentially being matrices, can be manipulated mathematically to morphologically alter shapes or extract features vital to any study. MATLAB, a computing environment suited to matrix manipulation was used to extract and quantify all data used in this study. In particular, the Image Processing Toolbox was used to extract the desired features.

The images used in this study contained red-stained cells, which were easily isolated by extracting the red channel. Every image was demarcated by a region of interest (ROI) in white that encapsulated the cells involved in feature extraction. Morphological operations were performed on the red channel within the ROI to condition the image for cell segmentation. Following cell segmentation and assigning an identification to each cell, various features such as Euclidean distances and orientation were extracted for statistical analysis.

### 2.1 Cell Segmentation (Marker-Controlled Watershed)

Prior to any feature extraction in biomedical images portraying cells, cellular segmentation needs to be achieved to construct symbolic representation of the cells in the image. There are various methods to achieve this, some as simple as thresholding an image such that all pixels above a certain intensity value are equated to one, and all pixels below equated to zero. However, this method poses two problems. The primary problem occurs in overlapping cells, or cells that are closely joined together, where thresholding may bundle multiple cells as a singular object, as shown in fig. i. Approximately 10 cells are bounded within the green circle in fig i.a, which translated into a single object following thresholding in fig. i.b. The secondary problem occurs when background noise makes its way through thresholding, seen by tiny specks in fig. i.b. The specks can be incorrectly labelled as actual cells, which is highly undesirable to maintain accuracy and precision in segmentation.

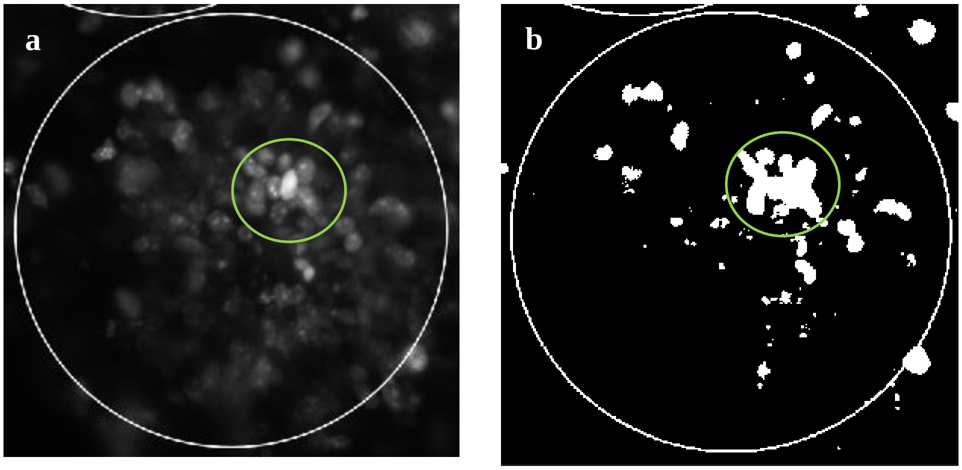


**Figure I.** A region of interest where **a** (left) shows the red channel and **b** (right) shows the thresholded imaged. The green marker shows cell bundles incorrectly segmented to a single object.

To overcome incorrect segmentation, marker-controlled watershed segmentation was used. Watershed segmentation consists of two components, a watershed and a catchment basin. In

simple terms, a watershed is a region that divides areas drained by different river systems and a catchment basin is the area draining into a river[22] . In terms of image segmentation, the catchment basin encompasses a set of pixels with minimum regional intensity such that a drop of water will flow down the relief to converge with the minimum intensity pixels[23] . The watershed will then be the line separating adjacent catchment basins[23] .

For further information on watershed segmentation and the mathematics governing said segmentation, we refer our readers to [23].

#### 2.1.1 Catchment Basin Identification

By default, running watershed segmentation in MATLAB with default parameters will result in good segmentation, but the results can be greatly improved by marking regions of local minima. This was performed using the *imextendedmax* function. This generates the extended-maxima transform for an image, which is the regional maxima of the H-maxima transformation.

The H-maxima transformation works by suppressing all maxima with intensity level lower or equal to a threshold value H[24] . This process smoothens out regions where drastic contrast changes occur, in a way such that regional maxima in an image will have the same value.

The extended-maxima transformation then determines the regional maxima of the H-maxima transformation[24] . This implies that connected pixels with highest values following smoothening by H-maxima transformation are extracted and displayed. There is a significant distinction between image maxima and extended-maxima because image maxima will only return pixels with the highest intensities in the image, whereas extended-maxima will return “regions” with highest intensities.

Fig. ii demonstrates the application of extended-maxima transformation onto the same ROI shown in fig. i, with the green circle representing the same bundle of cells and regions of maxima.

The points shown in fig. ii are the regional maxima, which are the opposite of catchment basins. The complement of this image results in the opposite, or regions of minima which form catchment basins.

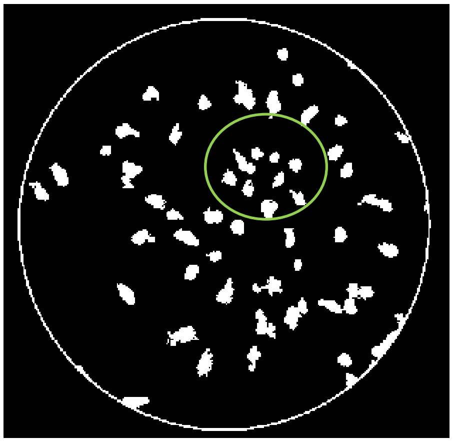

**Figure II:** Extended-maxima transformation showing regions of maximal intensity. The green circle shows the regions of maxima found in the bundle of cells.

The red channel is reconstructed by imposing the catchment basins and morphologically processed red channel on top on the original red channel to form image shown in fig. iii. This image is well-suited to perform watershed segmentation, where the catchment basins (black regions within cells) represent the cellular minima and the cellular region surrounding them represent the relief. It can be observed that each cell within the bundle has a catchment basin, with a relief surrounding them. A water droplet dropped into the relief can end up into any of these catchment basin, depending of which part of the relief the droplet drops onto. The presence of an obvious watershed leads to successful segmentation of cells touching or overlapping each other, provided that they are marked with a catchment basin.

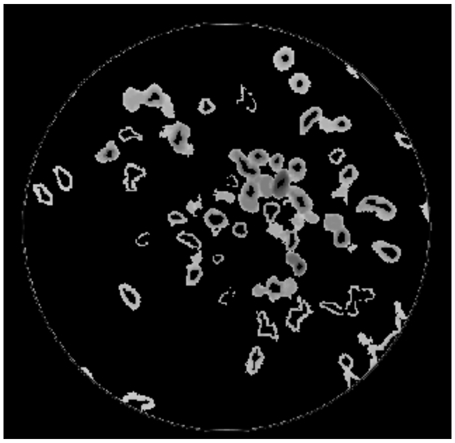

**Figure III:** Catchment basins imposed on top of the red channel. Black regions within cells show the catchment basins

### 2.1.2 Watershed Segmentation

The processed image in fig. iii was then processed using MATLAB's *watershed* function, which identifies the watershed regions in the image. Fig. iv shows the result of this segmentation, where each segmented region is represented by a different color.

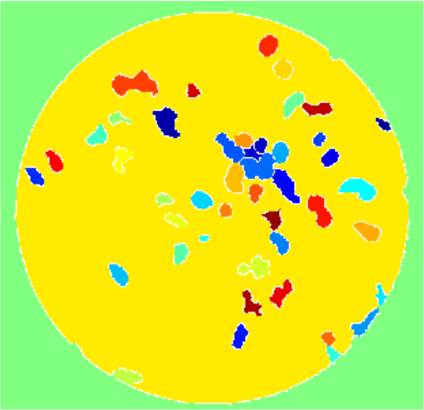

**Figure IV:** Watershed segmentation of image in fig. iii. Each segmented region represents a cell and is identified by a unique color. The bundle of cells previously mentioned as been successfully segmented into 7 cells, instead of a single object.

### 2.1.3 Cell Segmentation Overview for Ki67 and Calretinin Images

This section is an overview for the methods covered above and their application to both Ki67 and calretinin images. Every step performed to achieve segmentation and corresponding outputs are shown.

Ki67 and calretinin images were marked with a white circular ROI encompassing regions related to this study. In addition, a white reference line was placed onto the calretinin images for calculation of deviation angles. The reference line was isolated via Hough transform and its orientation was determined using the endpoints of the line. As cells pertaining to this study were stained red, the red channel was extracted from these images to isolate only the stained cells.

**Ki67** **Calretinin**

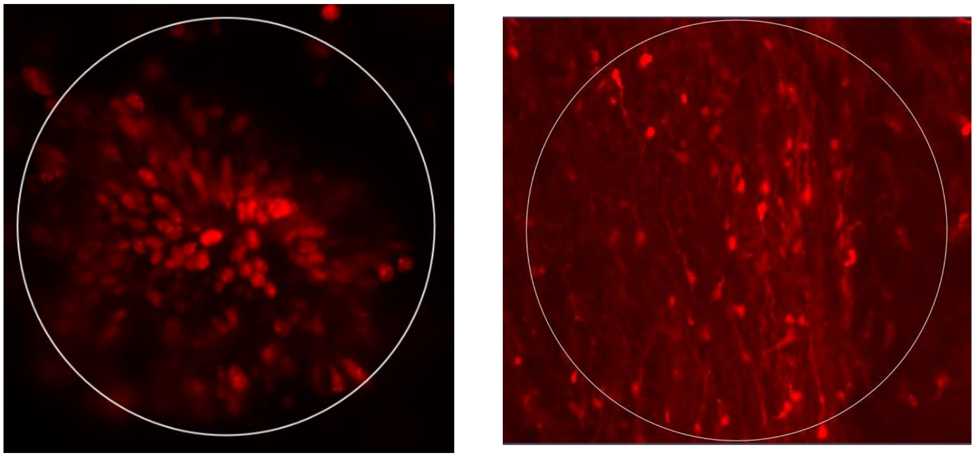

Red channel extracted and pre-processed with a top-hat filter to correct uneven illumination

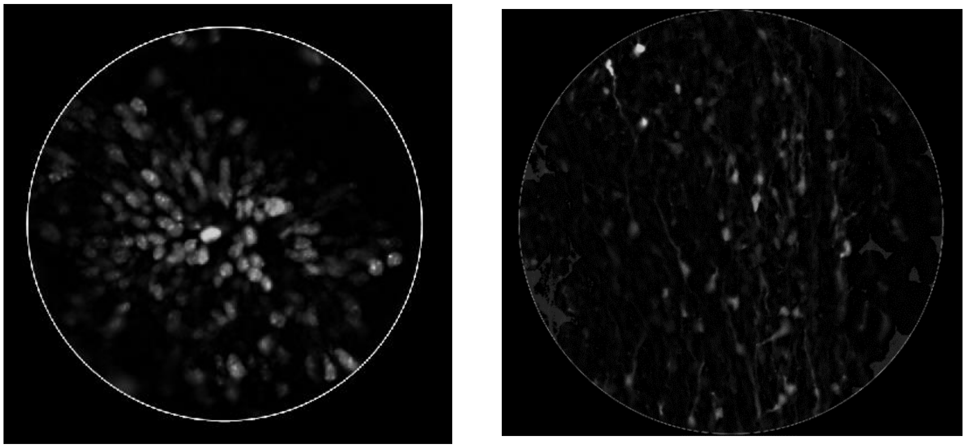

Contrast limited adaptive histogram equalization (CLAHE) to enhance regions in image and to evenly distribute pixel intensities across image to bring out details in image for determination of extended-maxima.

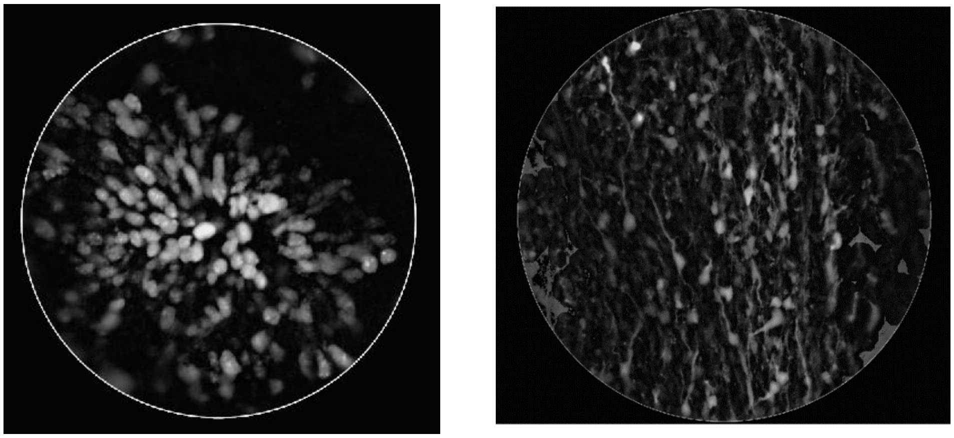

Determination of extended-maxima to locate catchment basin for watershed segmentation

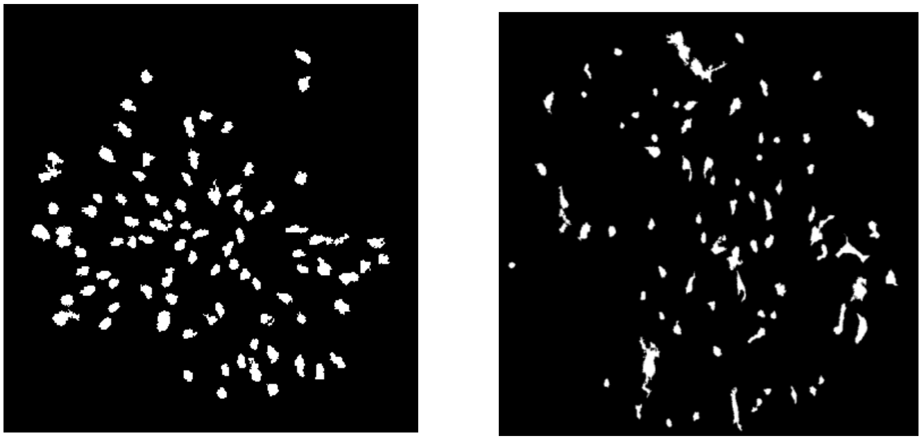

Extended-maxima image morphologically cleaned and noise (tiny pixels) removed using *bwareaopen*

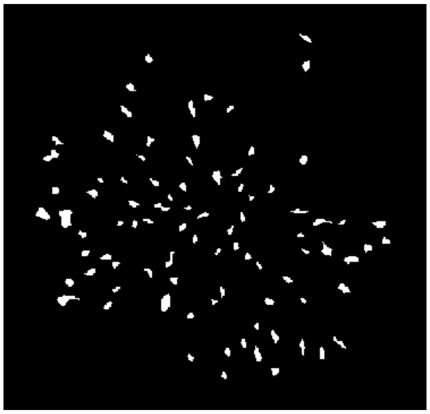 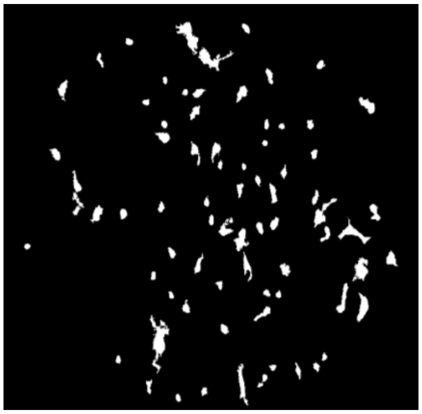

Complement of equalized red channel was obtained to prepare for imposing catchment basins

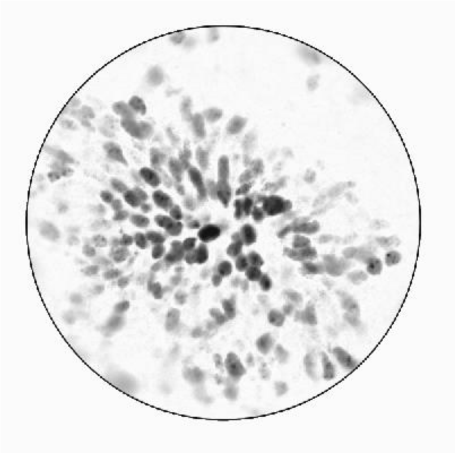 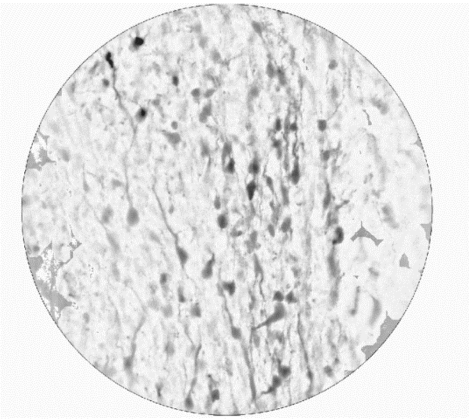

Catchment basin imposed on complement of previously equalized image to prepare for watershed segmentation. The method for imposing differ between Ki67 and claretinin images, resulting in the difference between the two images.

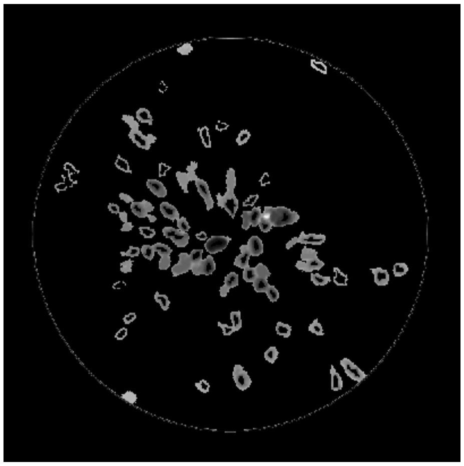

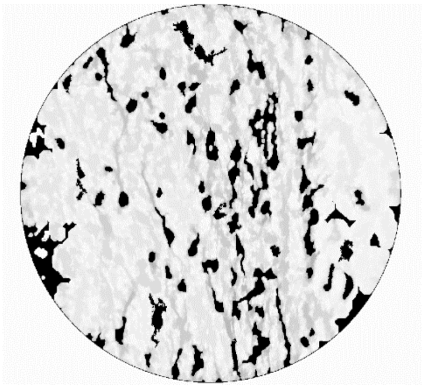

Image underwent watershed segmentation, where adjacent catchment basins were separated by a watershed.

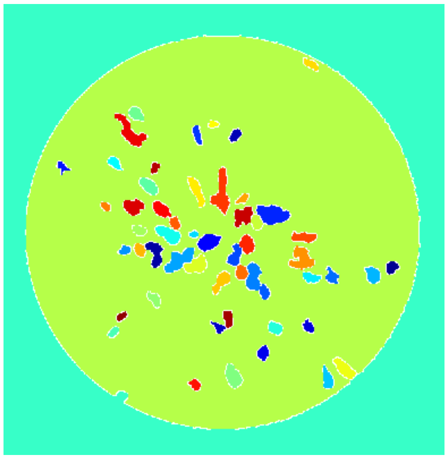

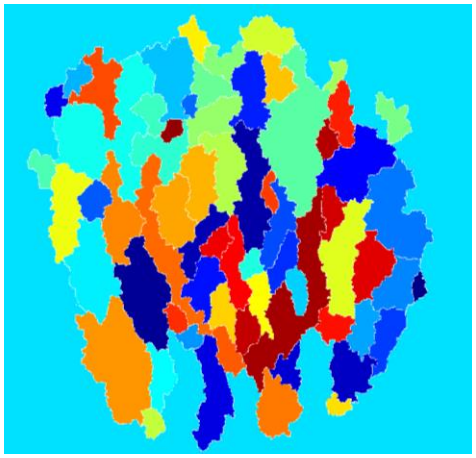

Watershed transform results in the formation of a watershed label matrix, where each segmented cell or cell containing region is numbered. With cell segmentation out of the way and each cell identified, various features (addressed in the following sections) were extracted.

## 2.2 Euclidean Minimum Spanning Tree (Ki67)

The watershed segmented image was parsed through MATLAB's *regionprops* function, which measures various properties of objects in an image. In Ki67 images used for this study, *'centroid'* was the property to be measured. The centroid returns the coordinates of center-of-mass of objects specified in the watershed label matrix, in the Cartesian coordinate system. With the coordinates defined, each cell is established as a node with spatial position for calculating Euclidean distances.

The Euclidean minimum spanning tree (EMST) connects these nodes with lines (also called edges) such that the sum of length of these lines is minimized. An ROI with *N* number of nodes will have (*N-1)* lines and the lines are laid out such that every node can be reached by following the line. Delaunay triangulation is a precursor to determining EMST as EMST is essentially an edge reduction of Delaunay triangulation of *N* nodes.

The EMST is important for the purpose of this study as it shows the least distance between cells in an ROI and their connectivity. We hypothesized a change in spatial cell distribution between control and schizophrenia, and the best representation of these changes would be distances between cells.

### 2.2.1 Delaunay Triangulation

In a Euclidean plane with finite points, triangulation is defined as a planar graph that meets the criteria: (1) every face is a triangle representing the convex hull of set of points, (2) no two edges ever intersect, and (3) no point lies on an edge[25-27] .

Delaunay triangulation is a is a method used to achieve triangulation of data set through empty circumcircle criterion[26] . In other words, a circle intersecting the three vertices of a triangle will contain no other point(s)[26]. . Fig. v visualizes the effect of a triangulation of vertices V1 – V4, where one is a legal triangulation and the other is not.

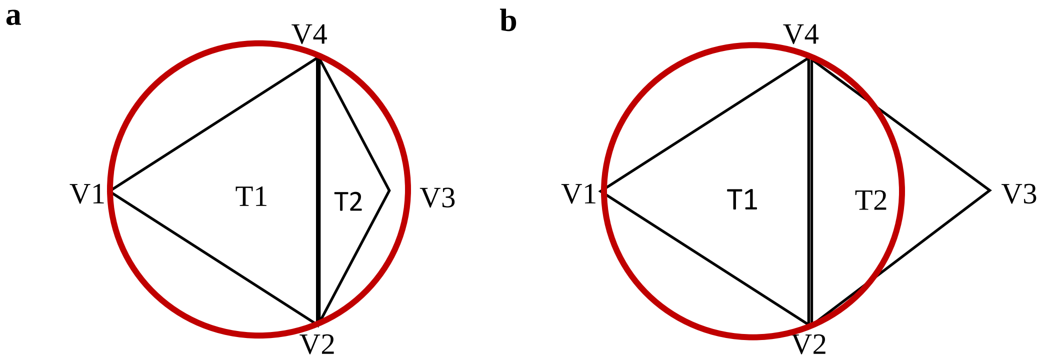


**Figure V:** Example of Delaunay triangulation by visualizing circumcircle in red, where **a** (left) shows an illegal Delaunay triangle as a circumcircle intersecting vertices of T1 encompasses vertex V3. **b** (right) on the other hand shows a legal Delaunay triangle since the vertex V3 belonging to T2 is not part of the circumcircle.

Naturally, an extension to this criterion is that larger internal angles be present in the formation of a Delaunay triangle[26] . Smaller base angles at vertices V2 and V4 correspond to V3 being closer to the base of the triangle and thus being within the circle. Larger base angles on the other hand leads to V3 being outside the circumcircle, thereby fulfilling the Delaunay triangulation criterion.

EMST, as mentioned above requires the calculation of *(N-1)* edges for *N* points such that the sum of all edges is minimized. Typically, all possible pairwise Euclidean distances between points is computed, resulting in *N(N-1)/2* edges[27] . Computing these edges takes a considerably long time, and produces immense amount of edges that can be very hard to manage. In an ROI containing 100 cells, this translates to 4,950 edges to be computed, and then minimizing the sum of these edges. Utilizing Delaunay triangulation reduces this workload as the number of edges reduces to less than *3*N* when vertices are legally triangulated[25]. Effectively, the computation time is reduced in *3N → (N-1)* than *N(N-1)/2 → (N-1)*, or in the case of 100 cells, 300 edges need to minimized instead 4,950 edges.

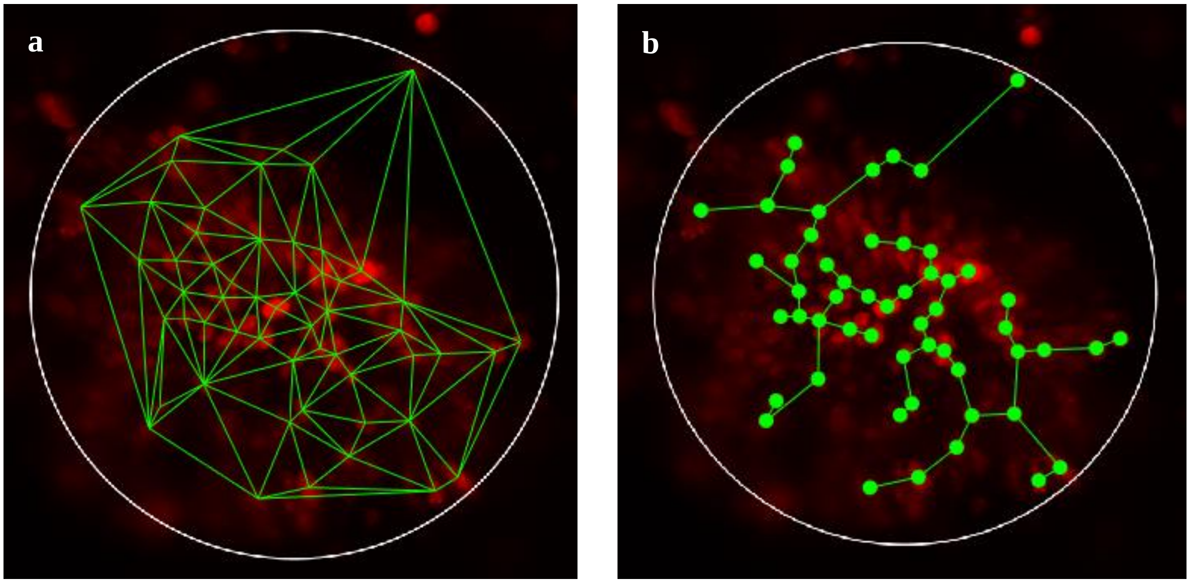


**Figure VI: a** (left) shows Delaunay triangulation of an ROI, with cells acting as nodes and the green lines representing edges. **b** (right) shows the MST obtained from triangulation edges.

Fig. vi.a is a representation of the application of Delaunay triangulation to this study. It can be observed that all triangulation rules and Delaunay criterion are being met. The next step performed is reduction of triangulation to EMST.

### 2.2.2 Prim's Algorithm for EMST

When forming any minimum spanning tree, two important criteria need to be met: (1) sum of edges is minimized and (2) no closed loops can be formed. Prim's algorithm is a minimum cost

spanning tree that utilizes a greedy approach in determining the minimum spanning tree. Cost in this context refers to the Euclidean distances between nodes, previously referred to as edges.

A brief approach of the algorithm is as follows[28] :

1. Choose an arbitrary node as the starting node
2. Check for all edges protruding from that node. In this case, the edges are formed using Delaunay triangulation.
3. Compare the edges, pick the one with the lowest cost and assign it to the starting node.
4. The edge chosen in the previous step leads to another node, which is then grouped with the starting node to reduce the pair of nodes to a single node.
5. Repeat steps 3-4 until all nodes are connected.

Successful completion of the algorithm produces an EMST depicted in fig. vi.b, where there are no closed loops and every node can be accessed by following the edges. The reduction of a Delaunay triangulation from *~3*N* edges to *(N-1)* edges can also be seen fig vi, where the shortest edges remain in EMST, while the larger edges are discarded.

## 2.3 Deviation Angle and Major Axis Length (calretinin)

Neurons located close to the surface of the cerebral organoid were found to be oriented tangential to the surface in control images, and more random in schizophrenia images. To quantify this phenomenon, the deviation angle of these neurons from a reference line tangent to the cerebral organoid's surface was used.

We also hypothesized that there is a difference in length of processes on neurons between control and schizophrenia images, so a length parameter was also calculated. The methods employed for these calculations are described in the section below.

MATLAB's *regionprops* function was used to return orientation and major axis length of ellipses that have the same second-moments as the watershed segmented regions. These ellipses are visualized in fig. vii, where each detected neuron is bounded by a green ellipse. The ellipses represent an elliptical estimation of the region bounding a neuron.

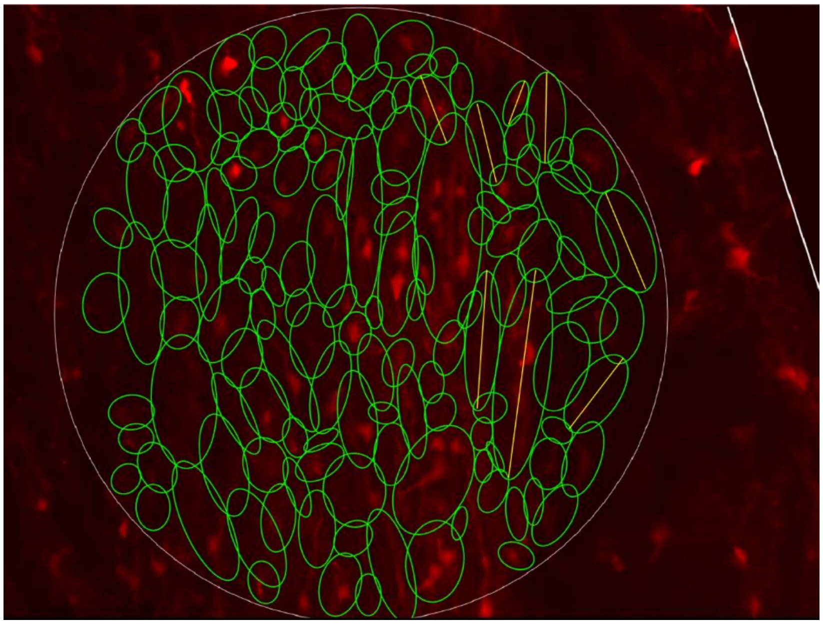

**Figure VII:** A calretinin ROI showing elliptical estimation of regions bounding a neuron.

In addition, an eccentricity parameter was also obtained through the *regionprops* function, which reflects closeness of an ellipse to a circle or line segment. Elliptical representation of neurons with larger process length tends to produce a larger major axis length and thus a larger eccentricity value. This allowed us to group neurons according to those with and without processes.

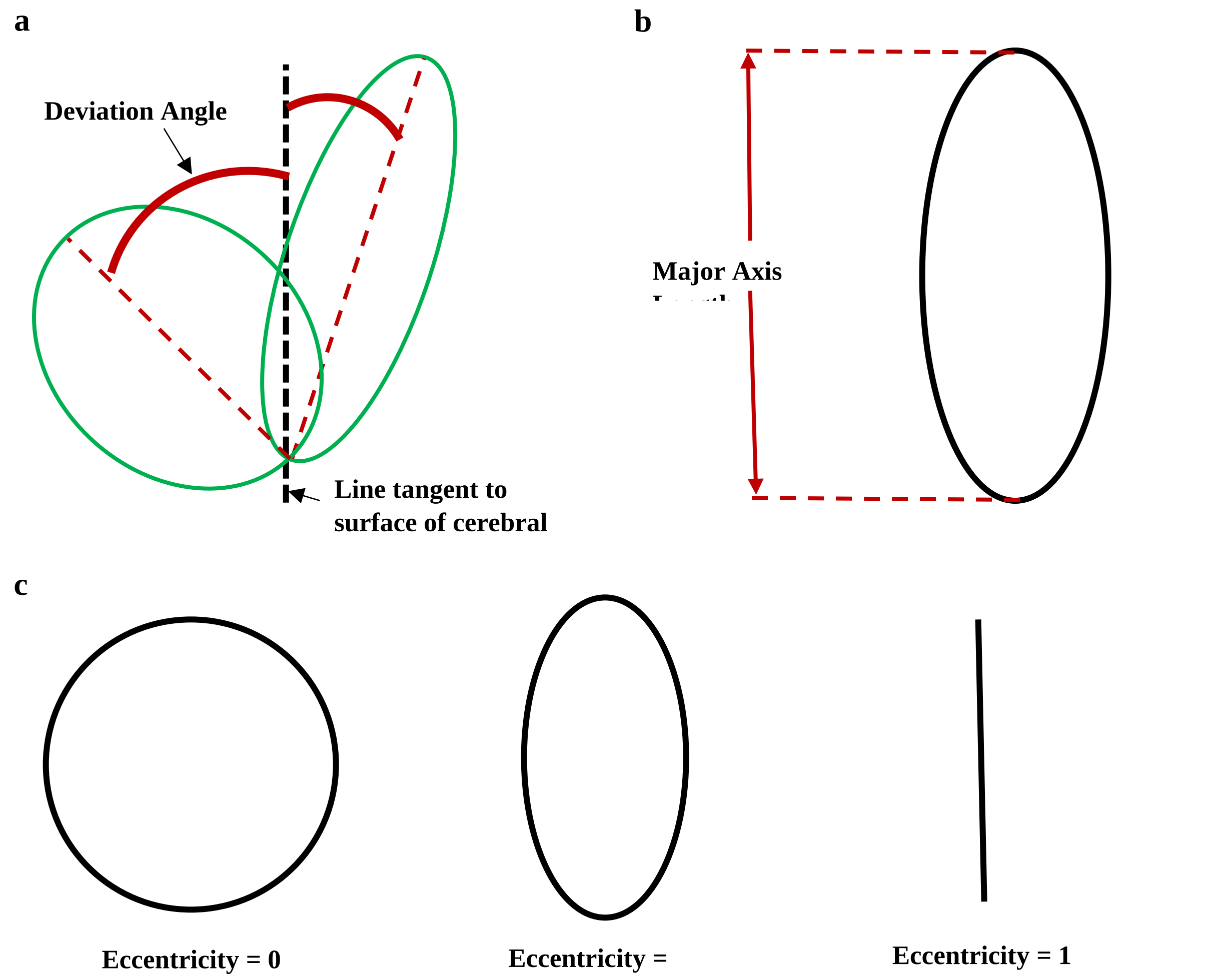


**Figure VIII:** Illustration of parameters extracted per cell in calretinin images where **a** (top-left) shows deviation angle, **b** (top-right) shows major axis length, and **c** (bottom) shows eccentricity.

The three parameters were extracted for each neuron detected in calretinin images and they are illustrated in fig. viii.

## 2.4 Data Matrices

All extracted parameters were stored in individual data matrices for control and schizophrenia, per image set (Ki67 and calretinin). The data matrices list the file number, ROI index, and parameters. All statistics and figures generated in this paper fetched information from the data matrices, ensuring easy access to the raw data.

## References


1. Narla, S.T. *et al.* Common developmental genome deprogramming in schizophrenia - Role of Integrative Nuclear FGFR1 Signaling (INFS). *Schizophrenia research* **185**, 17-32 (2017).
2. Stachowiak, M.K., Maher, P.A., Joy, A., Mordechai, E. & Stachowiak, E.K. Nuclear accumulation of fibroblast growth factor receptors is regulated by multiple signals in adrenal medullary cells. *Molecular biology of the cell* **7**, 1299-1317 (1996).
3. Somanathan, S., Stachowiak, E.K., Siegel, A.J., Stachowiak, M.K. & Berezney, R. Nuclear matrix bound fibroblast growth factor receptor is associated with splicing factor rich and transcriptionally active nuclear speckles. *Journal of cellular biochemistry* **90**, 856-869 (2003).
4. Maher, P.A. Nuclear Translocation of fibroblast growth factor (FGF) receptors in response to FGF-2. *The Journal of cell biology* **134**, 529-536 (1996).
5. Reilly, J.F. & Maher, P.A. Importin beta-mediated nuclear import of fibroblast growth factor receptor: role in cell proliferation. *The Journal of cell biology* **152**, 1307-1312 (2001).
6. Stachowiak, M.K., Maher, P.A., Joy, A., Mordechai, E. & Stachowiak, E.K. Nuclear localization of functional FGF receptor 1 in human astrocytes suggests a novel mechanism for growth factor action. *Brain research. Molecular brain research* **38**, 161-165 (1996).
7. Peng, H. *et al.* Novel nuclear signaling pathway mediates activation of fibroblast growth factor-2 gene by type 1 and type 2 angiotensin II receptors. *Molecular biology of the cell* **12**, 449-462 (2001).
8. Stachowiak, E.K., Fang, X., Myers, J., Dunham, S. & Stachowiak, M.K. cAMP-induced differentiation of human neuronal progenitor cells is mediated by nuclear fibroblast growth factor receptor-1 (FGFR1). *J Neurochem* **84**, 1296-1312 (2003).
9. Bryant, D.M. & Stow, J.L. Nuclear translocation of cell-surface receptors: lessons from fibroblast growth factor. *Traffic* **6**, 947-954 (2005).
10. Coleman, S.J. *et al.* Nuclear translocation of FGFR1 and FGF2 in pancreatic stellate cells facilitates pancreatic cancer cell invasion. *EMBO molecular medicine* **6**, 467-481 (2014).
11. Clarke, W.E., Berry, M., Smith, C., Kent, A. & Logan, A. Coordination of fibroblast growth factor receptor 1 (FGFR1) and fibroblast growth factor-2 (FGF-2) trafficking to nuclei of reactive astrocytes around cerebral lesions in adult rats. *Molecular and cellular neurosciences* **17**, 17-30 (2001).
12. Gonzalez, A.M., Berry, M., Maher, P.A., Logan, A. & Baird, A. A comprehensive analysis of the distribution of FGF-2 and FGFR1 in the rat brain. *Brain Res* **701**, 201-226 (1995).
13. Fang, X., Stachowiak, E.K., Dunham-Ems, S.M., Klejbor, I. & Stachowiak, M.K. Control of CREB-binding protein signaling by nuclear fibroblast growth factor receptor-1: a novel mechanism of gene regulation. *J Biol Chem* **280**, 28451-28462 (2005).
14. Leadbeater, W.E. *et al.* Intracellular trafficking in neurones and glia of fibroblast growth factor-2, fibroblast growth factor receptor 1 and heparan sulphate proteoglycans in the injured adult rat cerebral cortex. *J Neurochem* **96**, 1189-1200 (2006).
15. Lee, Y.W. *et al.* NGF-induced cell differentiation and gene activation is mediated by integrative nuclear FGFR1 signaling (INFS). *PloS one* **8**, e68931 (2013).
16. Lee, Y.W. *et al.* A novel nuclear FGF Receptor-1 partnership with retinoid and Nur receptors during developmental gene programming of embryonic stem cells. *Journal of cellular biochemistry* **113**, 2920-2936 (2012).
17. Narla, S.T. *et al.* Activation of developmental nuclear fibroblast growth factor receptor 1 signaling and neurogenesis in adult brain by alpha7 nicotinic receptor agonist. *Stem cells translational medicine* **2**, 776-788 (2013).
18. Narla, S.T. *et al.* Common developmental genome deprogramming in schizophrenia - Role of Integrative Nuclear FGFR1 Signaling (INFS). *Schizophrenia research* (2017).

19. Peng, H. *et al.* Integrative nuclear FGFR1 signaling (INFS) pathway mediates activation of the tyrosine hydroxylase gene by angiotensin II, depolarization and protein kinase C. *J Neurochem* **81**, 506-524 (2002).
20. Terranova, C. *et al.* Global Developmental Gene Programing Involves a Nuclear Form of Fibroblast Growth Factor Receptor-1 (FGFR1). *PloS one* **10**, e0123380 (2015).
21. Trapnell, C. *et al.* Differential gene and transcript expression analysis of RNA-seq experiments with TopHat and Cufflinks. *Nat Protoc* **7**, 562-578 (2012).
22. Eddins, S. (Matworks, 2002).
23. L. Belaid , W.M. IMAGE SEGMENTATION: A WATERSHED TRANSFORMATION ALGORITHM. *Image Analysis & Stereology* **28**, 93 (2009).
24. Soille, P. *Morphological image analysis*. (Springer, Berlin; 2010).
25. G. Hong-mei, X.C., Y. Ben-cheng Design and Analysis of Minimum Spanning Tree in Euclidean Plane. *International Conference on Computational and Information Sciences* (2013).
26. (Mathworks.
27. Berg, M. *Computational geometry*. (Springer, Berlin; 2010).
28. Prim, R.C. Shortest Connection Networks and Some Generalizations. *Bell System Technical Journal* **36**, 1389-1401 (1957).

# 3.0 SUPPLEMENTARY FIGURES:

**Supplementary Figure 1 – (a)** Protocol for generation of cerebral organoids using human iPSCs (shown) and hESCs**.**

iPSCs and HUES8 – Cells were maintained on gelatin coated plates with MEFs in hES medium. The protocol for neural induction in 3D cultures used in the present study is a combination of two protocols. First, we generated EBs using the NPC protocol developed in K. Brennand's laboratory [24], grown for 4-5 days. Afterwards, EBs were manually picked and placed in neural induction media according to the protocol of Lancaster et al.[20] and grew for 4 days. Within four days, they formed bright marginal zones. Neuroepithelia were transferred into matrigel droplets and grown in cerebral organoid media without vitamin A and stationary for 4 days. Then, day 1 organoids were transferred to a shaker and grown in cerebral organoid media with vitamin A. Organoids were harvested and fixed in 4% paraformaldehyde, incubated in a sucrose gradient: 7.5%, 15%, 30%, and then frozen on the 4$^{th}$ day in sucrose/gelatin in liquid nitrogen. Organoids were stored at -80°C. Gelatin blocks were sectioned at 40 µm using a Microtome Cryostat HM 500.

H9 hESCs - cells were maintained on matrigel (Corning) according to WiCell's feeder-free protocol. hESCs were incubated with collagenase (1mg/ ml in DMEM-F12) for 1-2 hours. Lifted colonies were washed with DMEM-F12, resuspended in N2/B27 media, and transferred to 6-well low attachment plates (Corning) overnight. The embryoid bodies (EBs) were formed after 24 hours and they were fed every day with N2/B27 media supplemented with dual SMAD inhibitors for 3-4 days [24]. After 4-5 days, the EBs were transferred one by one to a 24-well low attachment plate using a cut-pipette tip and incubated with neural induction medium. The neuroepithelial tissues were fed every other day for 4 days. Afterward, the neuroepithelial tissues were transferred to matrigel droplets on dimples of parafilm. The droplets were solidified at 37°C and the parafilm was removed subsequently. Neuroepithelia were grown in a stationary state for 4 days in cerebral organoid media without vitamin A. Day 1 cerebral organoids were transferred to an orbital shaker and were fed every 3-4 days with cerebral organoid media with vitamin A. Organoids were harvested and processed as described for iPSCs.

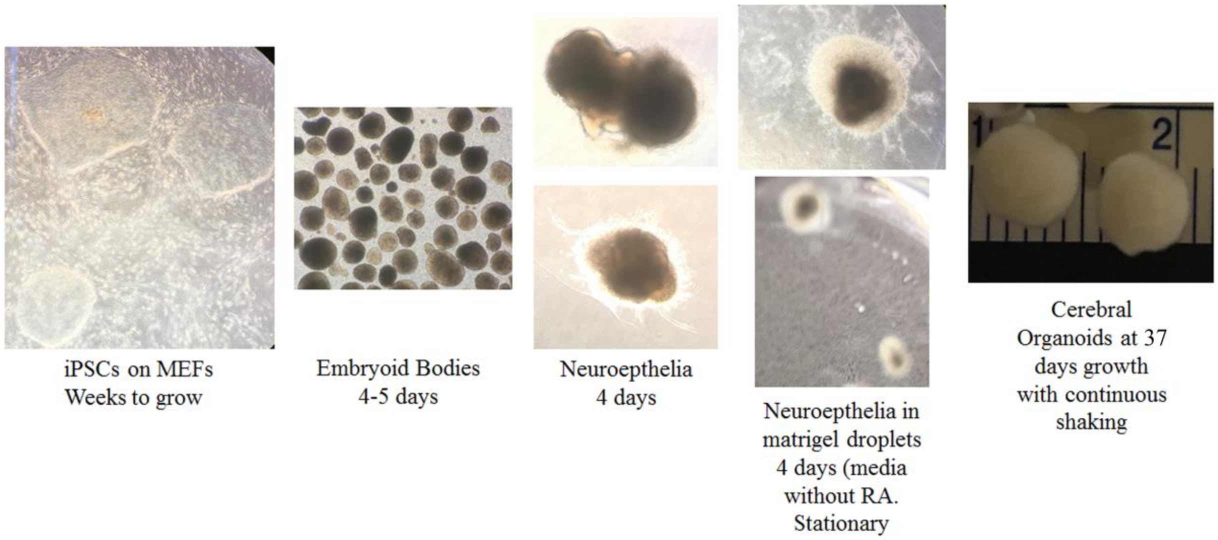


iPSCs on MEFs Weeks to grow

Embryoid Bodies 4-5 days

Neuroepthelia 4 days

Neuroepthelia in matrigel droplets 4 days (media without RA. Stationary

Cerebral Organoids at 37 days growth with continuous shaking

**Supplementary Figure 1** (**b**) Representative hESC (HUES8) cerebral organoids at 5 weeks of development. $b_1$ - tile scanning of DAPI; note forming rosettes with central ventricular zone (VZ), intermediate zone (IZ) and peripheral cortical zone (CZ). $b_2$ - immunostaining with anti-GFAP antibody reveals GFAP expressing radial glia concentrated in the VZ. $b_3$ - Ki67+ proliferating cell concentrate in VZ and some are found in IZ. Note absence of proliferating cells in CZ.

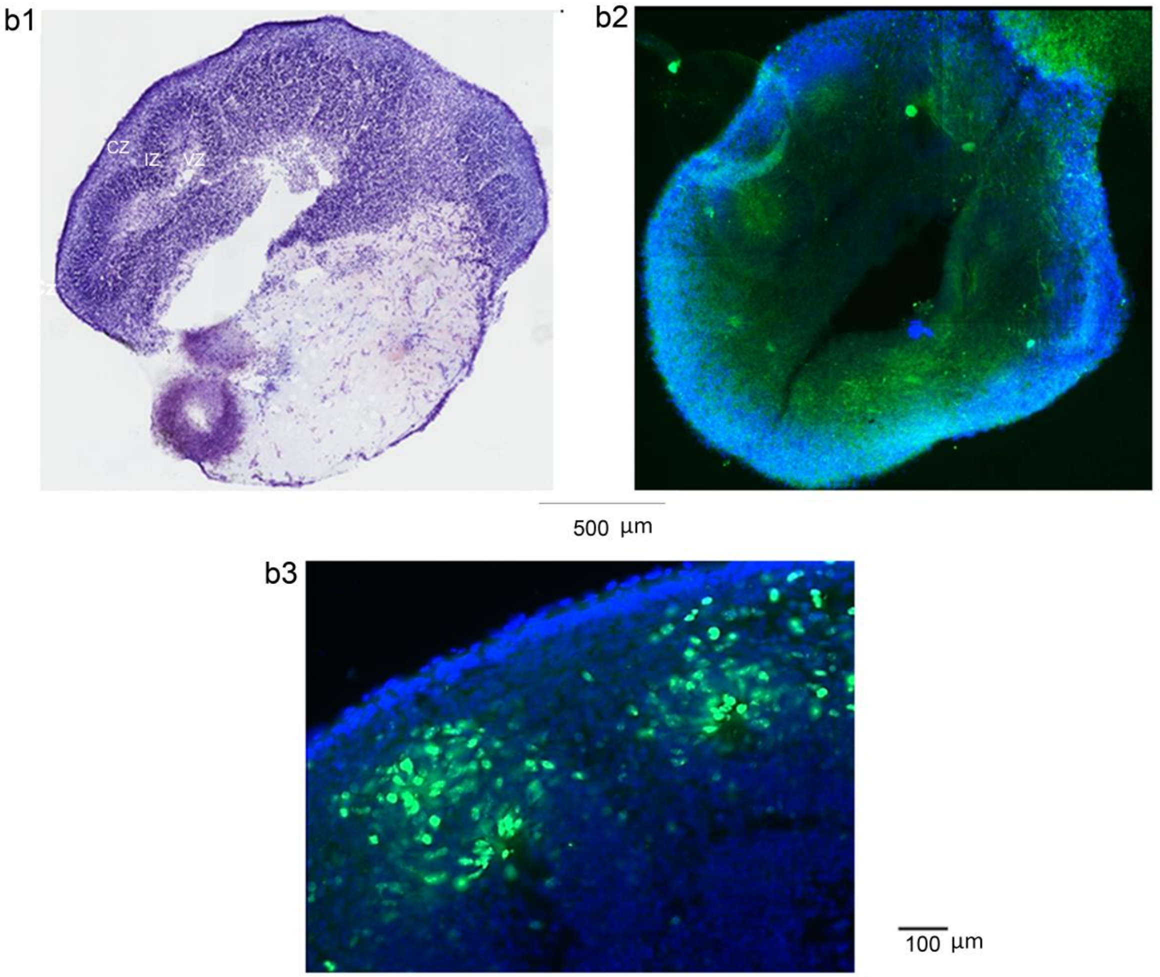

**Supplementary Figure 2 (**a) Examples of iPSC cerebral organoids at 5 weeks of development, DAPI stain, tile scanning. Control iPSC line BJ1, schizophrenia iPSC line 1835.

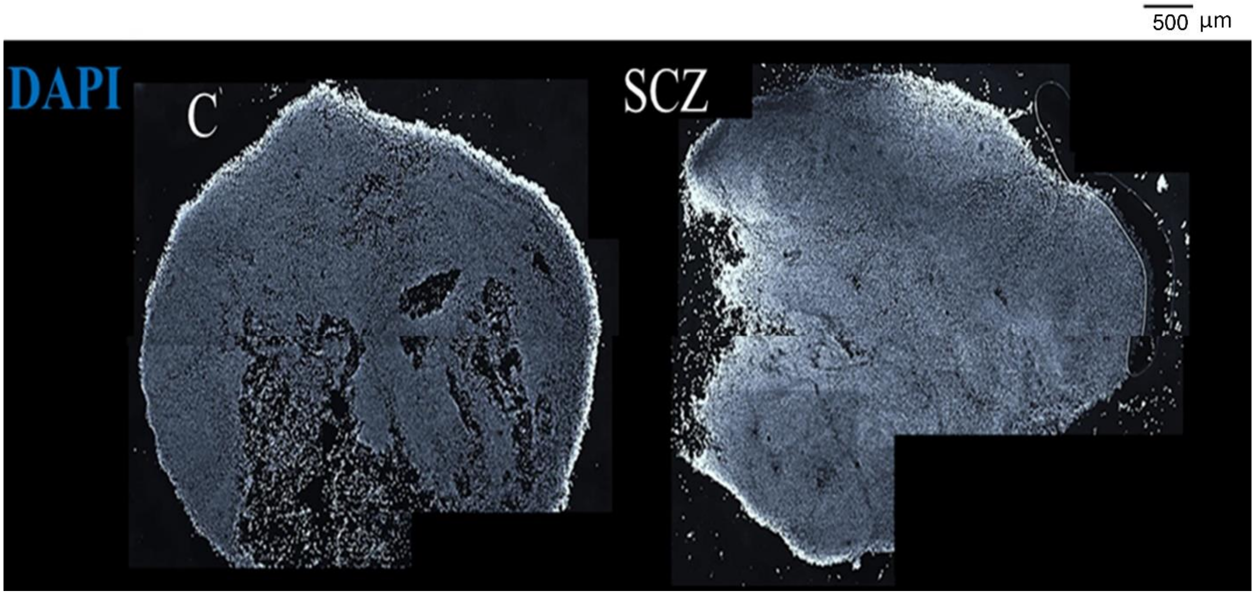


(b**)** Disorganized proliferation, cortical neuronal migration and poverty of cortical neurons in 2 week old schizophrenia iPSC cerebral organoids. Coimmunostaining for Ki67 (red), Pan-Neu (green), and DAPI (blue). Sections show representative images of control (b1; iPSC line 3651) and schizophrenia (b2; iPSC line 1835) organoids.

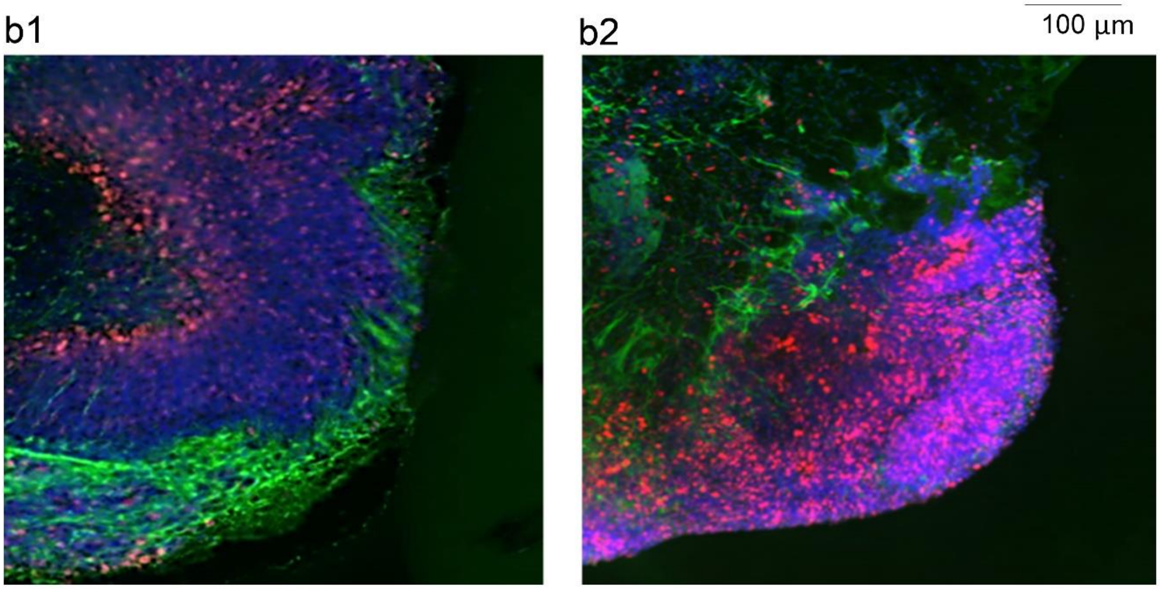

**Supplementary Figure 3** Counting calretinin expressing cortical interneurons in 5 week organoids. Total of 770 control and 547 schizophrenia calretinin interneurons were counted in 20 and 16 ROIs, respectively, in the organoids from three control and three schizophrenia patients. **(a)** The average cell densities per ROI were not significantly different in control and schizophrenia organoids. **(b)** Individual ROI value plots.

a.

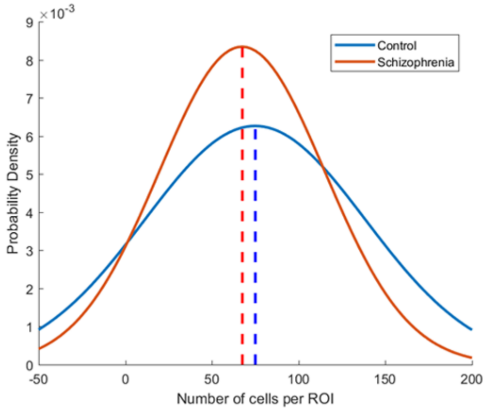


b.

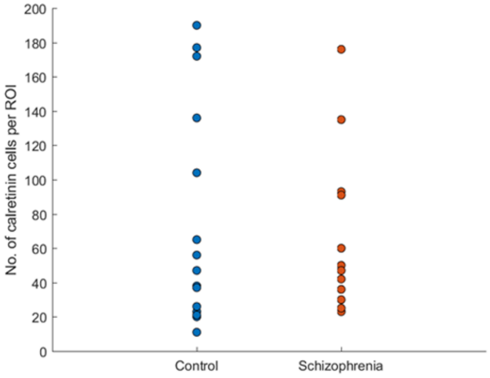


**Control**
Mean: 74.8667
SEM: 16.4461

**Schizophrenia**
Mean: 67.3333
SEM: 13.8029

**Supplementary Figure 4** (**a**) $a_1$-$a_3$ - Dual cytoplasmic and nuclear FGFR1 localization in the IZ and CZ cells of control (iPSC line BJ1) 2 week iPSC organoids. Nuclear speckles represent nFGFR1 associated with euchromatin sites of RNA co-transcriptional processing. a4 control (BJ1) and a5 schizophrenia (1835) images of organoid sections. FGFR1 (red), DAPI (blue).

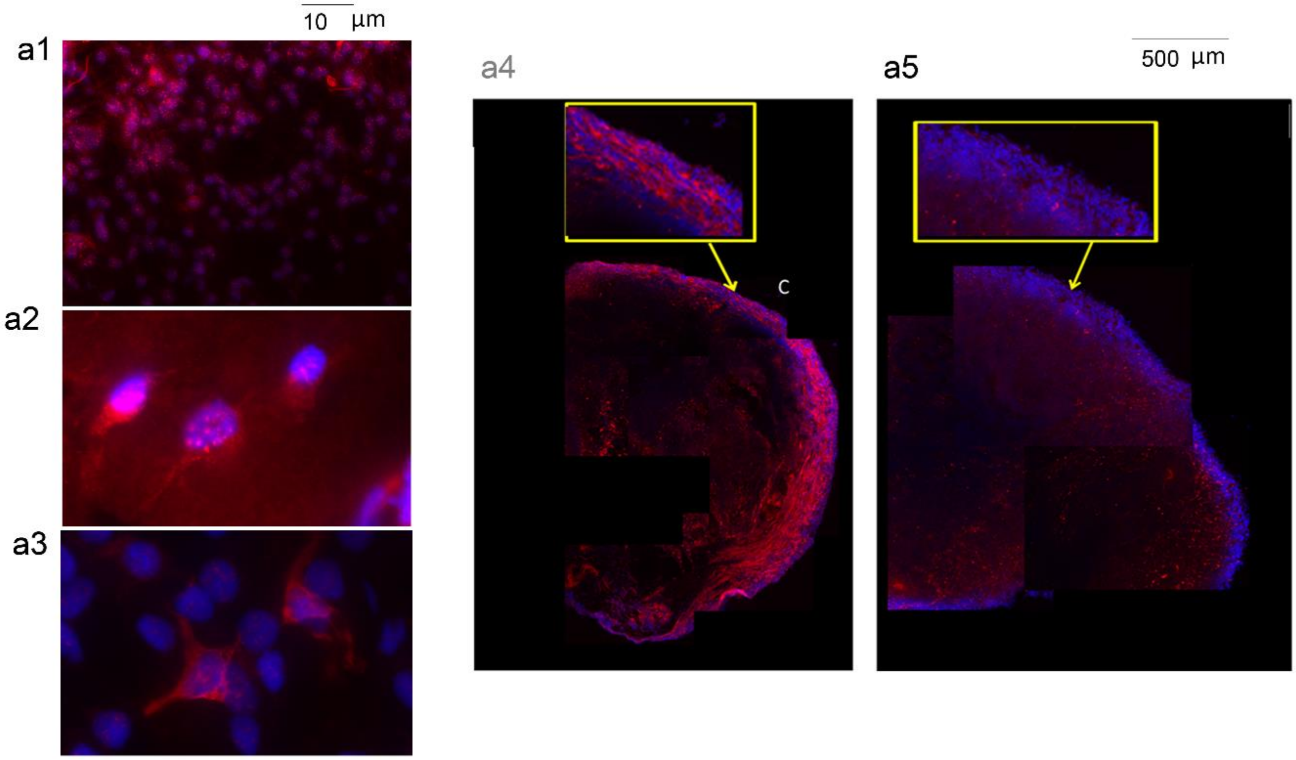

**Supplementary Figure 4 (b)** 5 week organoids: b1,b2 – control (iPSC line BJ1) organoids express FGFR1 in CZ and IZ, b1 inset shows negative control - omitted primary FGFR1 antibody. b3,b4 - schizophrenia (iPSC line 1835) organoids – depletion of FGFR1 immunostaining in CZ. Arrow points to nuclei with FGFR1.speckles.

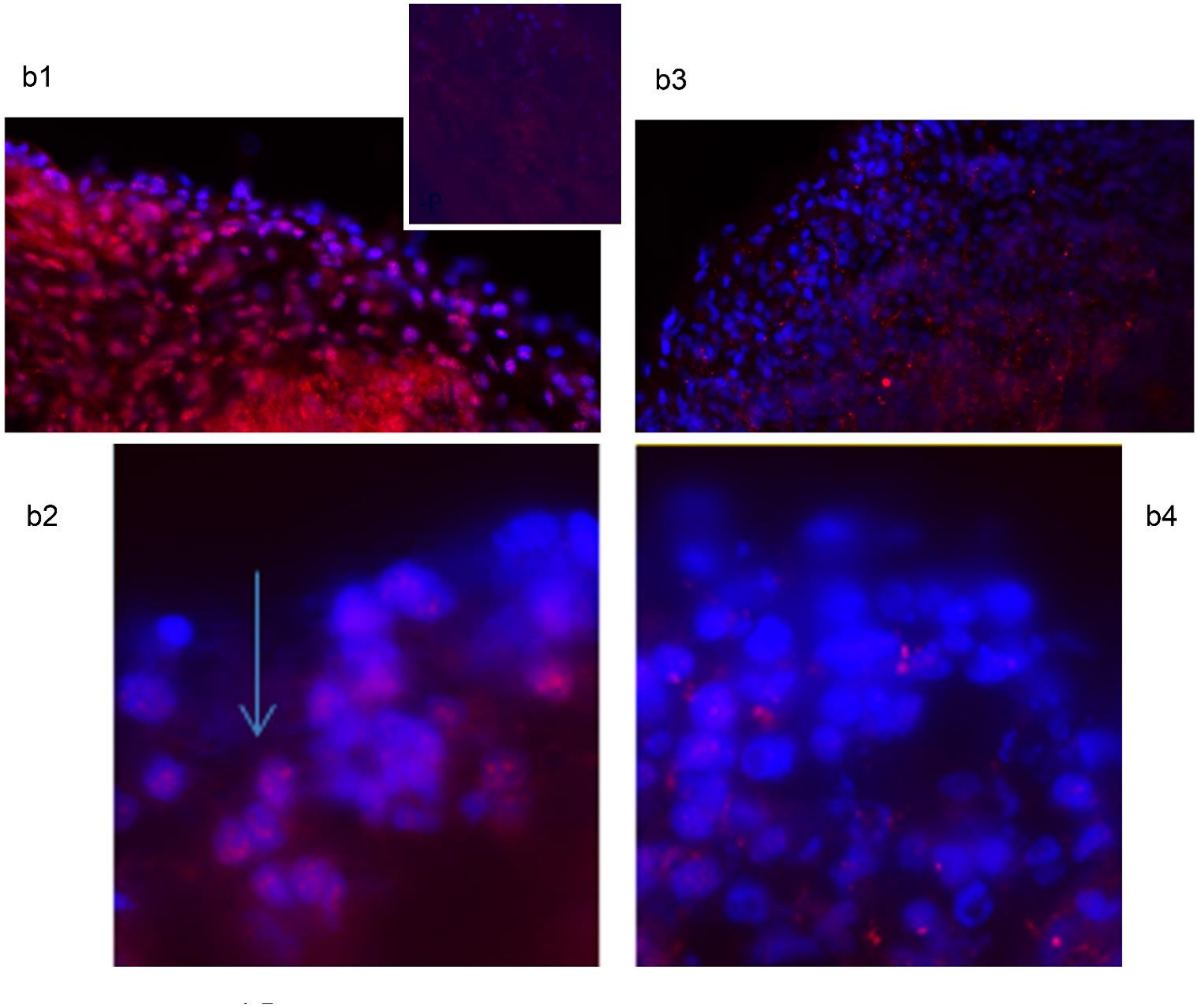

**Supplementary Figure 4 (c)** Staining for FGFR1 in 5 week organoids: c1 and c2 – 5 week control (iPSC line 3440) and c3, c4 – 5 week schizophrenia (line 2947) organoids**,** images show CZ and IZ (c1, c3) and IZ and VZ (c2, c4); c5 enlarged cortical cells of control (iPSC line 2937) and c6 schizophrenia (iPSC line 2038) show loss of nFGFR1 in schizophrenia cortex (in some cells FGFR1 is perinuclear). 3D rotational confocal images of control (line 3651) and schizophrenia (line 1835) organoids are shown in Video 2a and 2b.

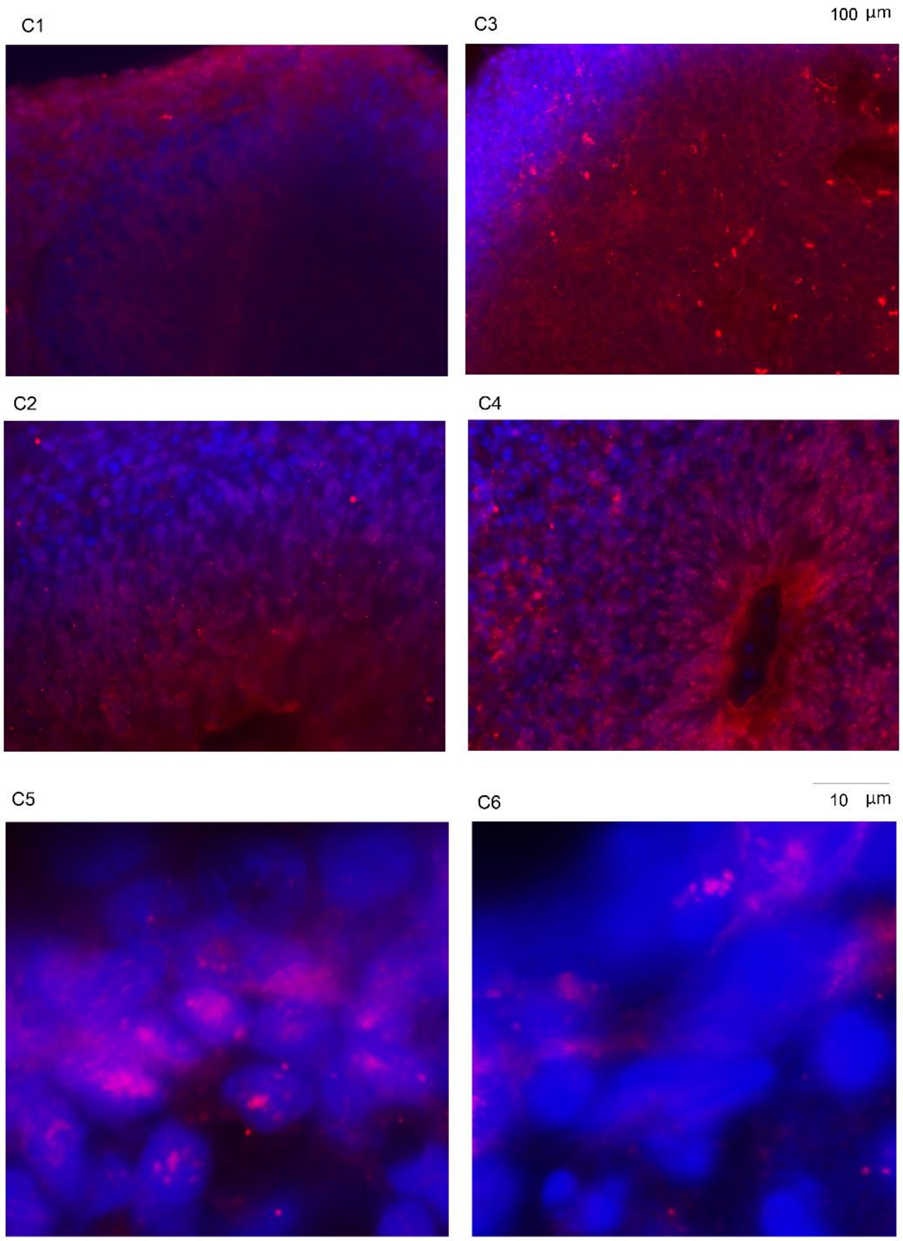

**Supplementary Figure 5** – Individual value plots for schizophrenia and control 5 week organoids: **(a-b)** The intensity of Pan-Neu (a) and Reelin (b) immunofluorescence intensities were quantified in randomly selected ROIs using Zen 2.0 Blue Imaging software in upper cortical and IZ regions (examples and statistical analyses of all data are shown on Figures 3d-f (Pan-Neu) and Figures 4b2, b3 (Reelin). The analyses were performed with 3 control individuals and 3 patients; number of superficial cortex ROIs. Circles represent intensities of the individual ROIs. **(c-d)** Percent of DAPI stained nuclei that colocalized with nuclear TBR1 (c) or nFGFR1 (d) immunofluorescence in upper cortical zone was quantified in randomly selected ROIs as described in Figures 4a, b (TBR1) and Figures 5b, c (nFGFR1). Analyses were performed on cerebral organoids of 3 schizophrenia patients and 3 control individuals. Circles represent individual ROIs.

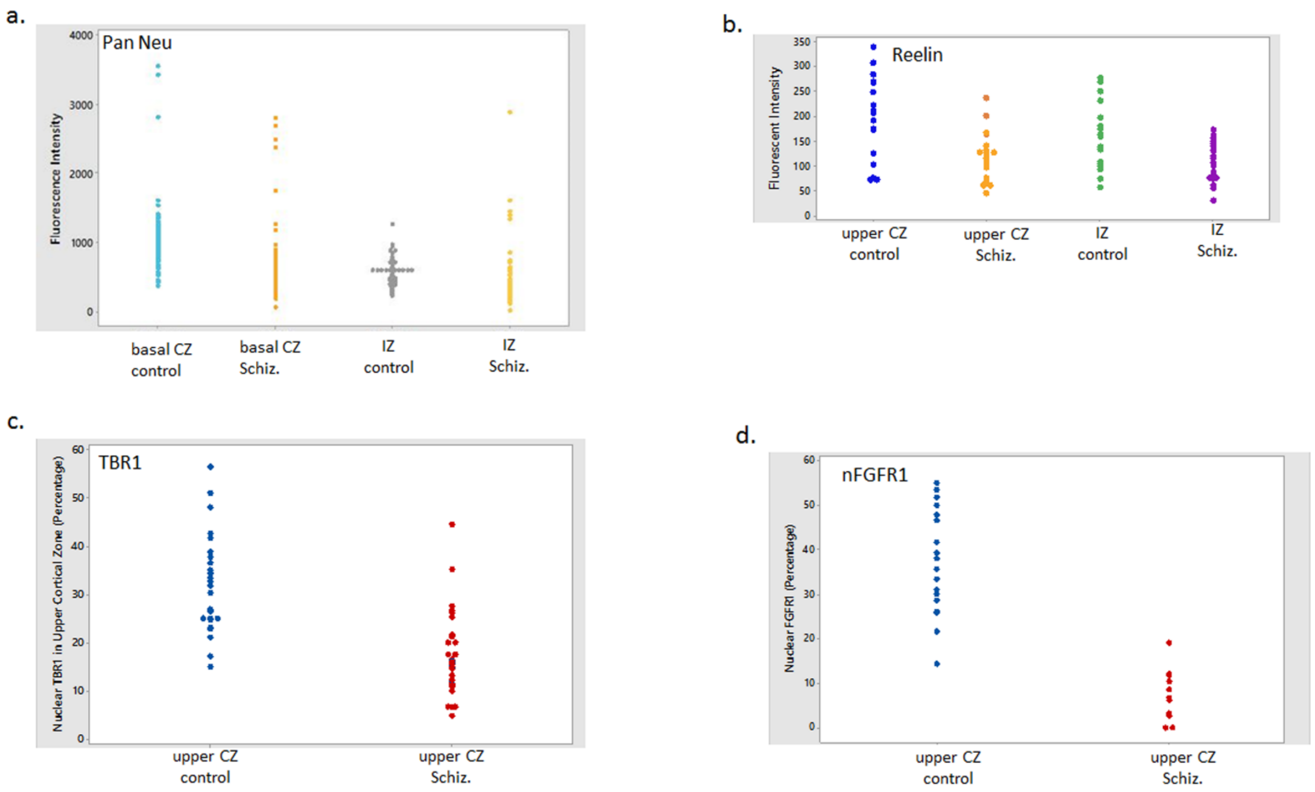

**Supplementary Figure 6 –** Pathways affected by dominant negative FGFR1(SP-/NLS)(TK- ) or constitutively active FGFR1(SP-/NLS) in neuronal committed cells (NCCs) induced with cAMP/BDNF/GDNF.

**a. Growth Hormone Receptor signaling**

cAMP/BDNF/GDNF + FGFR1(SP-/NLS)(TK-)

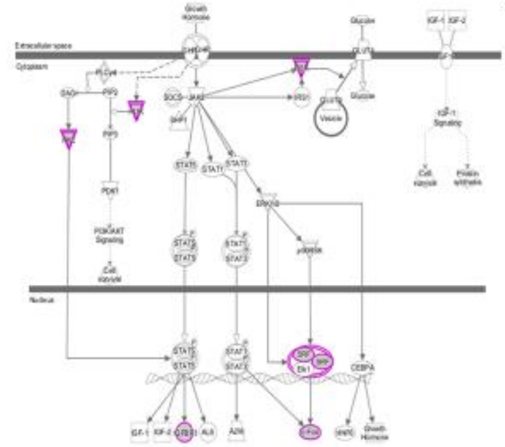

cAMP/BDNF/GDNF + FGFR1(SP-/NLS)

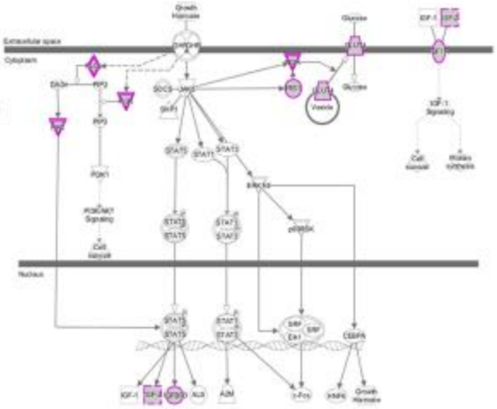

**b. RAR activation**

cAMP/BDNF/GDNF + FGFR1(SP-/NLS)(TK-)

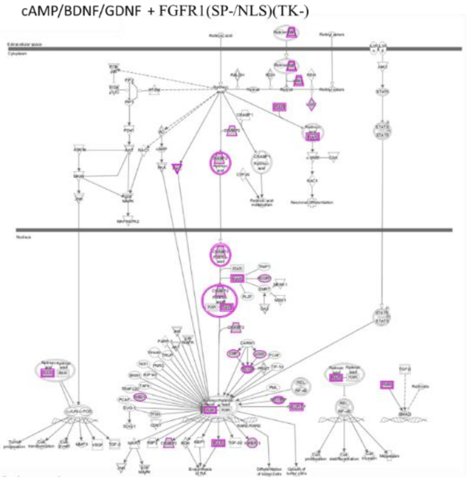

cAMP/BDNF/GDNF + FGFR1(SP-/NLS)

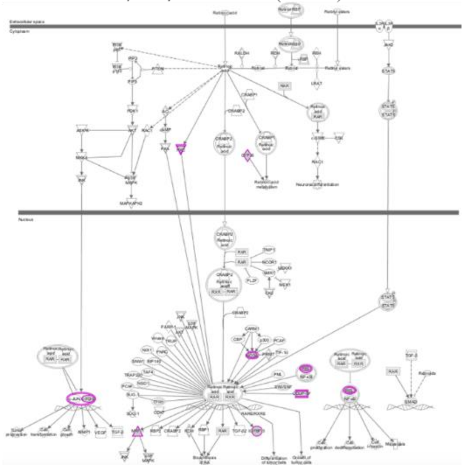

**c. STAT3 Pathway**

cAMP/BDNF/GDNF + FGFR1(SP-/NLS) (TK-)

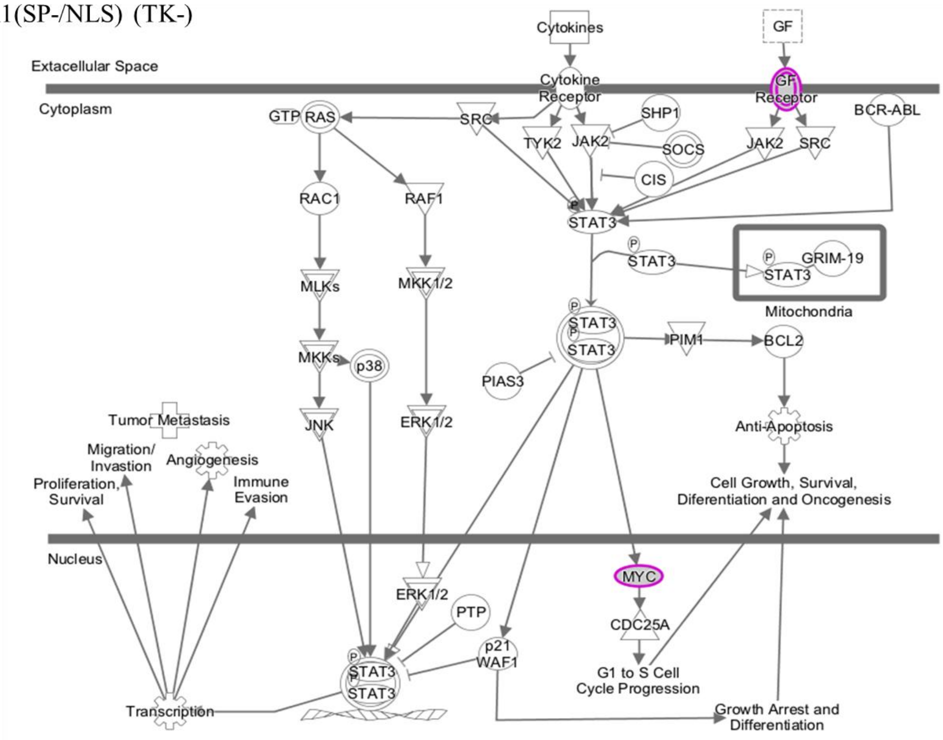


**d. Axonal guidance signaling**

cAMP/BDNF/GDNF + FGFR1(SP-/NLS) (TK-)

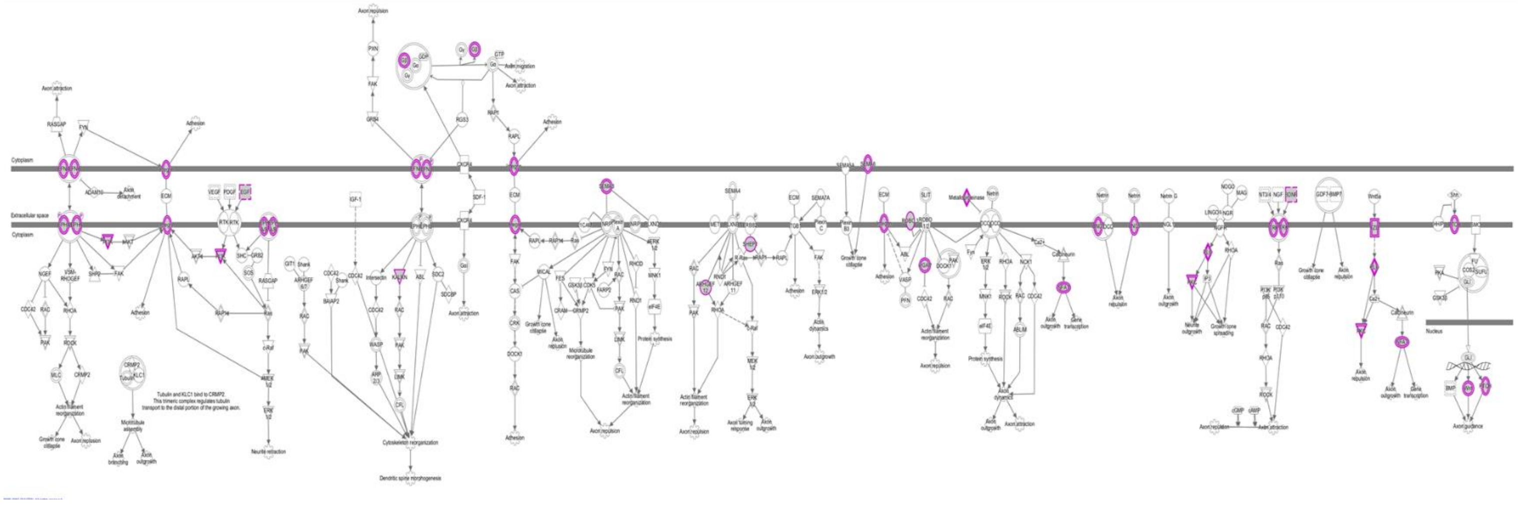

**e. Wnt/β-catenin signaling**

cAMP/BDNF/GDNF + FGFR1(SP-/NLS) (TK-)

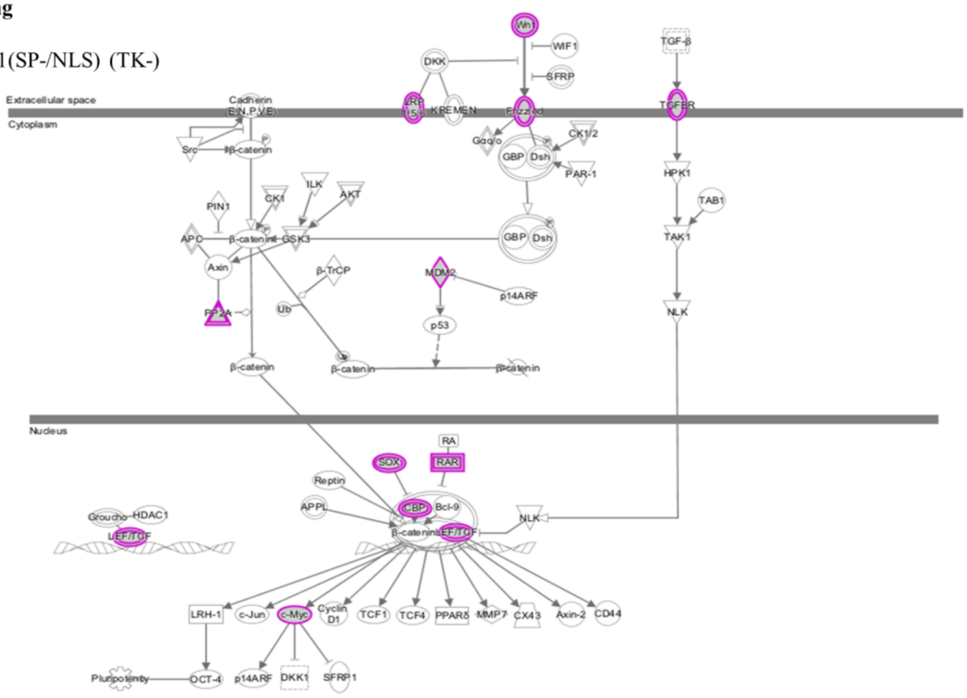


**f. Reelin signaling in neurons**

cAMP/BDNF/GDNF + FGFR1(SP-/NLS) (TK-)

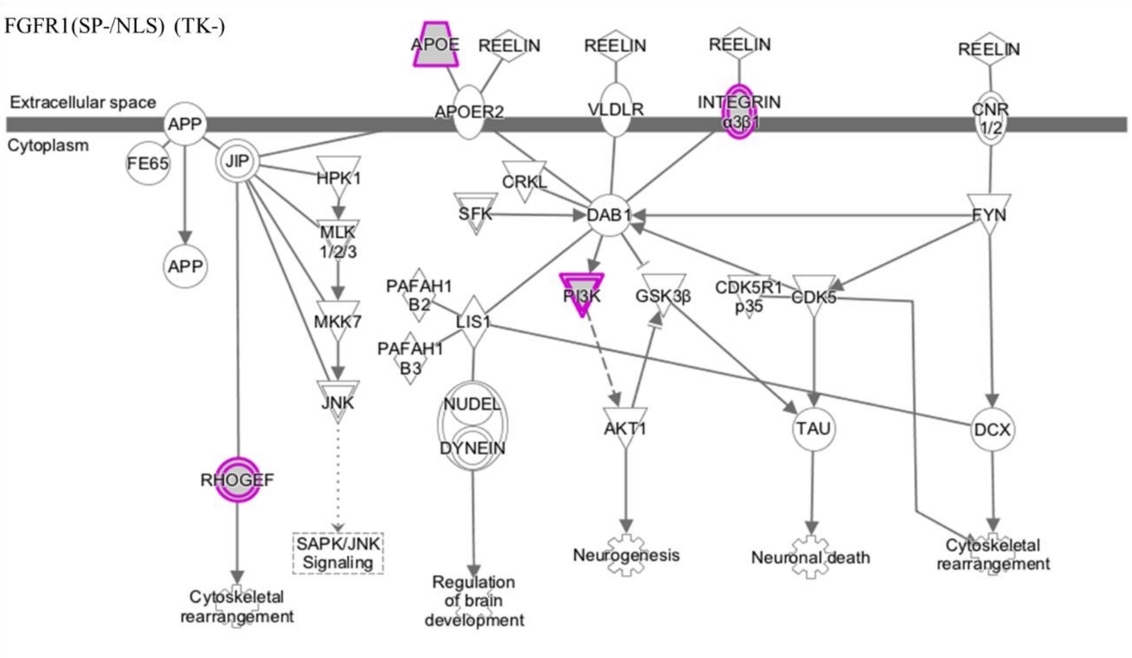

**g. CREB signaling in neurons**

cAMP/BDNF/GDNF + FGFR1(SP-/NLS) (TK-)

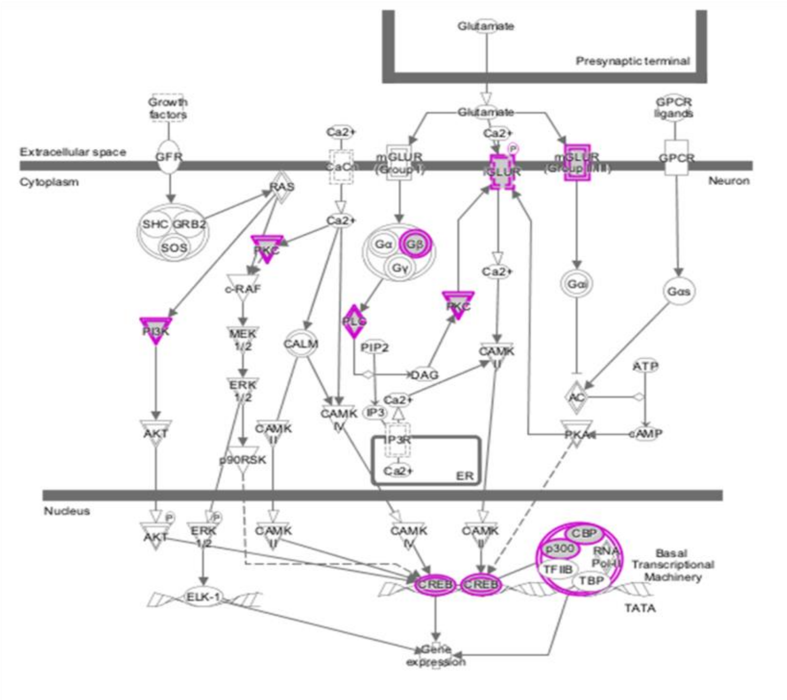


**h. Notch Signaling**

cAMP/BDNF/GDNF + FGFR1(SP-/NLS)

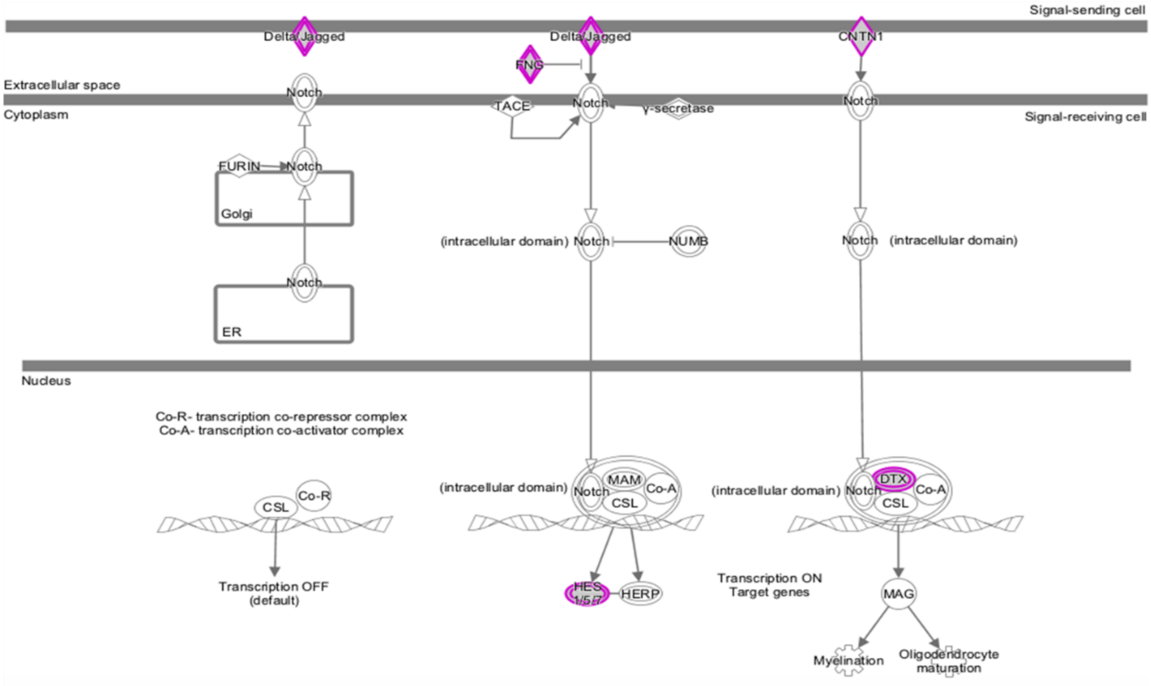

**Supplementary Figure 7 – (a)** Histogram of pairwise correlations. Correlation was performed using NPC and 3 NCC 4,704 genes differentially expressed between NPCs and NCCs. NPCs represent nondifferentiated neural progenitor cells and NCCs represent neuronal committed cells generated by NPC treatment with cAMP/BDNF/GDNF. Genes that showed the highest positive (+0.9 to +1.0) correlations (changes in the same direction) are marked by a gray bar. Genes that showed the highest negative (-0.9 to -1.0) correlations (changes in the opposite directions) are marked as a black bar.

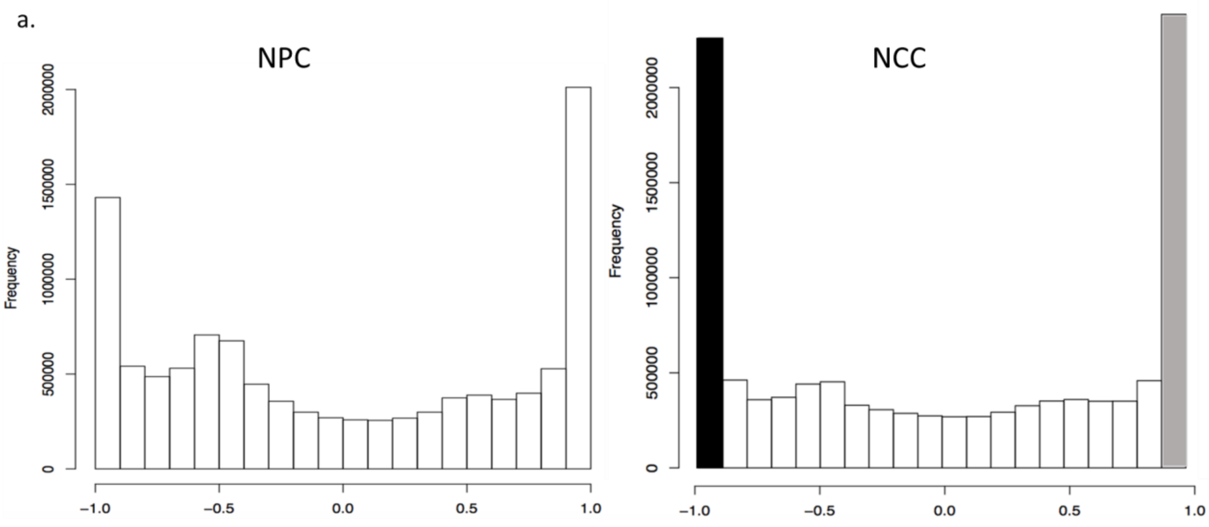

**Supplementary Figure 7 – (b-c**) Among genes that were differentially expressed between NPCs and NCCs, the top 200 nodes representing genes, which were positively correlated (b) or negatively correlated (c) in NCCs were analyzed. Grey lines link pairs of genes whose correlation is greater than 0.9. In the NCCs, separate positively (a) and negatively (b) correlated networks formed, which were absent in nondifferentiated NPCs.

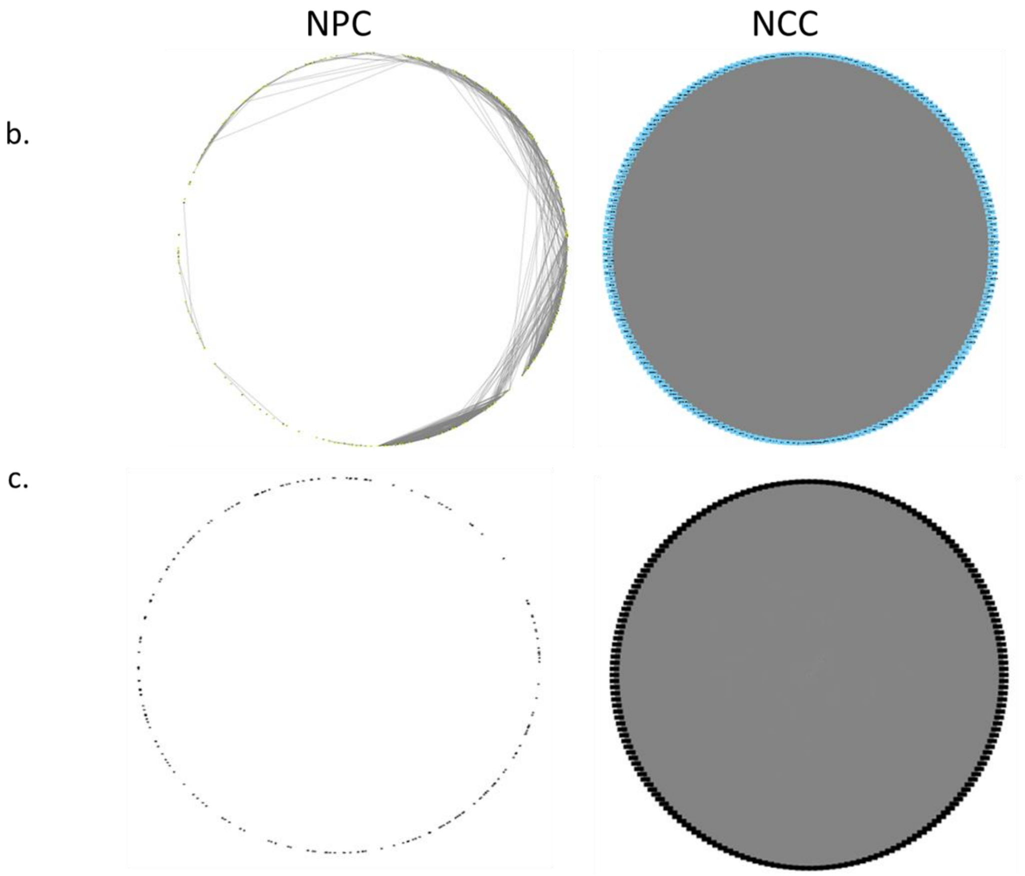

**Supplementary Figure 8 –** TBR1 expression is inhibited in hESC organoids by PD173074 treatment during days 8 to 18. **(a)** – control, **(b)** - PD173074.

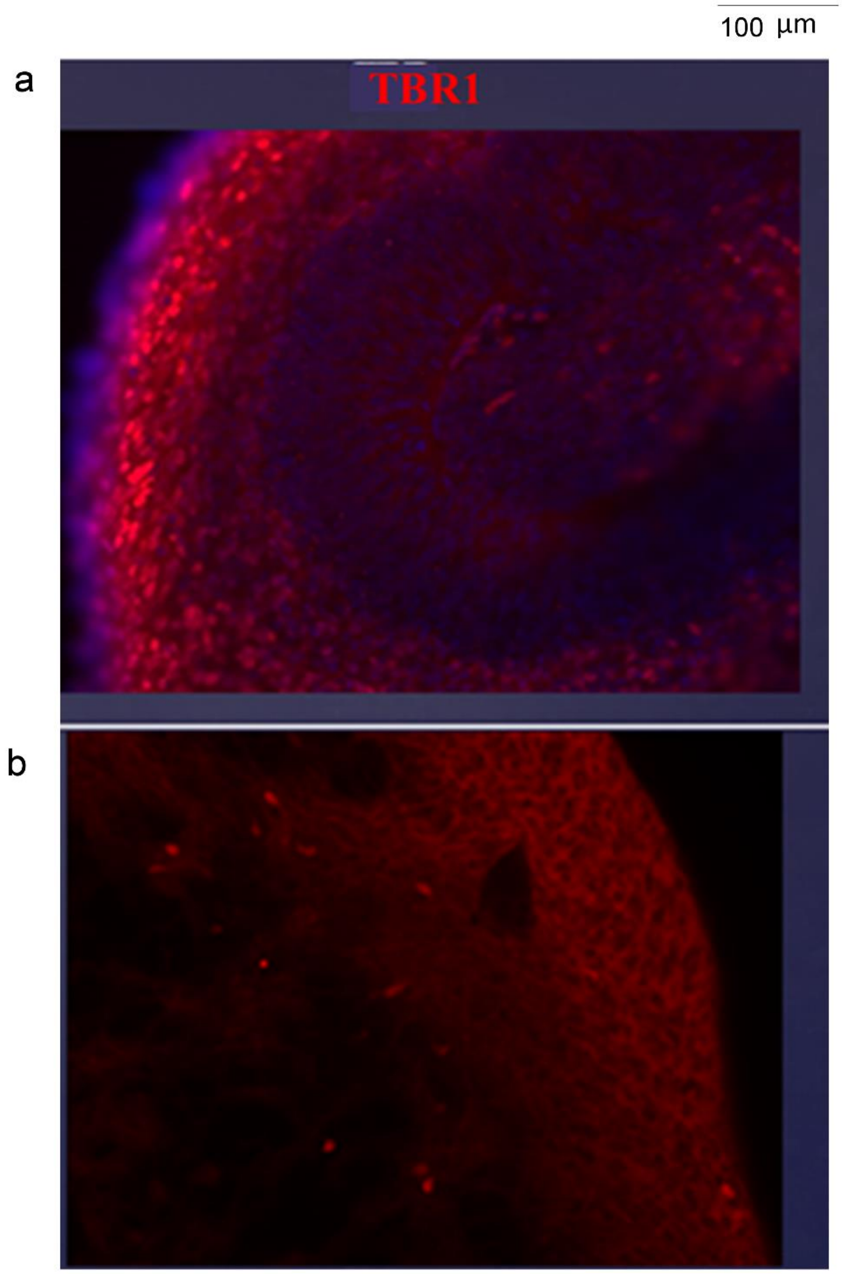

**Supplementary Figure 9 –** BrdU pulse-chase experiment. hESC organoids were treated with 100 nM PD173074 (days 8 to 18) and pulse labeled with BrdU at day 14. At day 18, control (**a**) and PD173074 treated organoids (**b**) were harvested and then coimmunostained for BrdU (red) and Pan-Neu (green). Arrows point to double labeled (BrdU+/Pan-Neu+) neurons in the marginal zone of the CZ. In PD173074 treated hESC organoids, note the subcortical and cortical BrdU labeled cells lacking Pan-Neu.

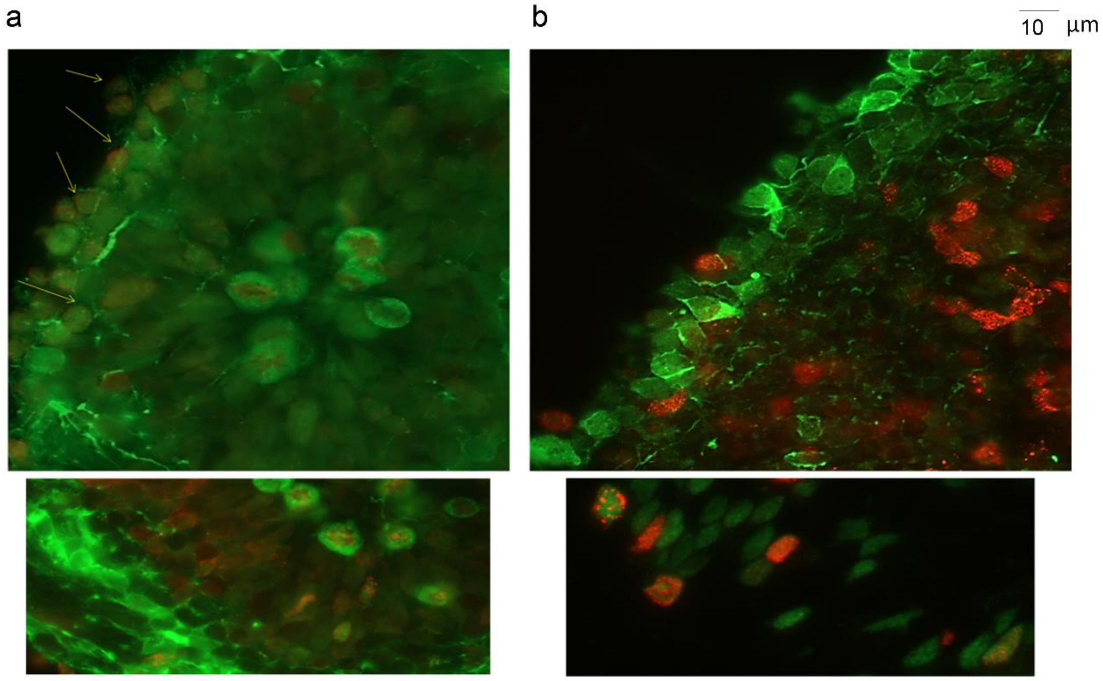

# 4.0 SUPPLEMENTARY VIDEOS LEGEND

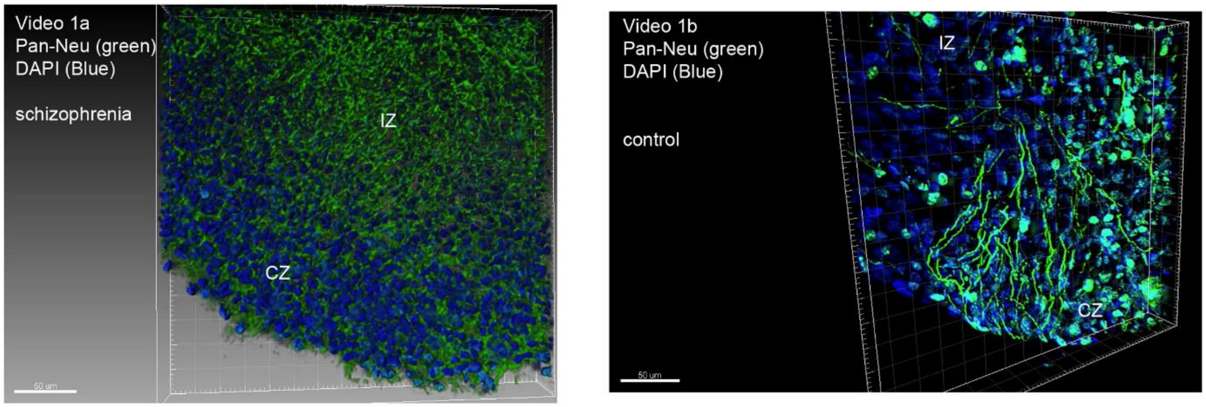


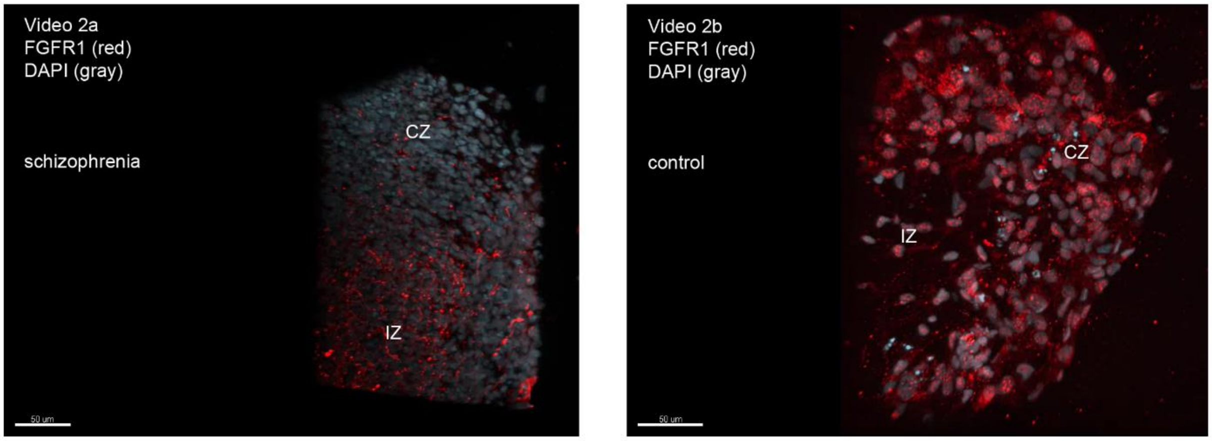


**Supplementary Video 1 legend:** (3D confocal images). The single frame image illustrates (**a**) underdevelopment of the cortical Pan-Neu neurons with long process and the increased density of subcortical Pan-Neu neurons in schizophrenia organoids (iPSC line 3651) compared to (**b**) control (iSPC line 1835). CZ – cortical zone, IZ – intermediate zone

**Supplementary Video 2 legend:** (3D confocal images). The single frame image illustrates (**a**) loss of FGFR1 immunostaining in the cortical cells and the presence in subcortical neurons in schizophrenia organoids (iPSC line 3651) compared to (**b**) control (iSPC line 1835). CZ – cortical zone, IZ – intermediate zone.

## 5.0 SUPPLEMENTARY TABLES:

| **Supplementary Table 1** - media composition and antibodies used | | |
|---|---|---|
| **Media** | **Reagents** | **Volume** |
| mTeSR Media (Use with Matrigel) | mTeSR1 basal medium (Stem Cell Technologies #5850) | 40 ml |
| | 5X mTeSR1 Supplement (Stem Cell Technologies #5850) | 10 ml |
| | 1X Penicillin/Streptomycin Solution (Corning #30-002) | 500 µl of 100X stock |
| hES Media (Use with MEFs) | DMEM/F12 + Glutamax (ThermoFisher Scientific #10565-042) | 38 ml |
| | 20% Knockout Serum Replacement (KOSR; ThermoFisher Scientific #10828028) | 10 ml |
| | 10 or 20 ng/ml FGF2 (ThermoFisher Scientific #PHG0261) | 1 ml of 1 µg/ml stock |
| | 1X MEM NEAA (ThermoFisher Scientific #11140050) | 500 µl of 100X stock |
| | 2-Mercaptoethanol (ThermoFisher Scientific #21985023) | 50 µl of 55 mM stock |
| | 1X Penicillin/Streptomycin Solution (Corning #30-002) | 500 µl of 100X stock |
| N2/B27 Media | DMEM/F12 + Glutamax (ThermoFisher Scientific #10565-042) | 48 ml |
| | 1x N2 Supplement (ThermoFisher Scientific #17502-048) | 500 µl of 100X stock |
| | 1x B27 Supplement Without Vitamin A (ThermoFisher Scientific #12587-010) | 1 ml of 50X stock |
| | 1X Penicillin/Streptomycin Solution (Corning #30-002) | 500 µl of 100X stock |
| N2/B27 media supplemented with dual SMAD inhibitors | DMEM/F12 + Glutamax (ThermoFisher Scientific #10565-042) | 48 ml |
| | 1x N2 Supplement (ThermoFisher Scientific #17502-048) | 500 µl of 100X stock |
| | 1x B27 Supplement Without Vitamin A (ThermoFisher Scientific #12587-010) | 1 ml of 50X stock |
| | 0.1 µM LDN193189 (Stemgent #04-0074) | 0.5 µL of 10mM stock |
| | 10 µM SB431542 (Tocris #1614/10) | 5 µL of 100mM stock |
| | 1X Penicillin/Streptomycin Solution (Corning #30-002) | 500 µl of 100X stock |
| Neural Induction Media | DMEM/F12 + Glutamax (ThermoFisher Scientific #10565-042) | 50 ml |
| | 1x N2 Supplement (ThermoFisher Scientific #17502-048) | 500 µl of 100X stock |
| | 1X MEM NEAA (ThermoFisher Scientific #11140050) | 500 µl of 100X stock |
| | 1 µg/ml Heparin (Stem Cell Technologies #07980) | 25 µL from 2 mg/ml stock |
| Cerebral Organoid Differentiation Media | DMEM/F12 + Glutamax (ThermoFisher Scientific #10565-042) | 25.5 ml |
| | CTS Neurobasal-A Media (ThermoFisher Scientific #A13710-01) | 25 ml |
| | 1X N2 Supplement (ThermoFisher Scientific #17502-048) | 250 µL of 100X stock |
| | 1X MEM NEAA (ThermoFisher Scientific #11140050) | 250 µL of 100X stock |

| | | |
|---|---|---|
| | Insulin Solution Human (Sigma #I9278-5ML) | 12.5 µL |
| | 2-Mercaptoethanol (ThermoFisher Scientific #21985023) | 17.5 µL of 55 mM stock Diluted 1:1000 |
| | 1x B27 Without Vitamin A (ThermoFisher Scientific #12587-010) or | 500 µl of 50X stock |
| | 1x B27 With Vitamin A (ThermoFisher Scientific #17504044) | 500 µl of 50X stock |
| | 1X Penicillin/Streptomycin Solution (Corning #30-002) | 500 µl of 100X stock |
| PD173074 | PD 173074 (Abcam #ab141117) Diluted in sterile DMSO to 100 µM | |
| | Diluted in Cerebral Organoid Differentiation Media to 100 nM | |

| **Primary Antibodies** | **Species** | **Dilution** |
|---|---|---|
| Anti-FGFR1 antibody (Abcam #ab823 -ab10646) | Ms, Rb | 1:1000 |
| Anti-Ki67 antibody (Abcam #ab15580) | Rb | 1:1000 |
| Anti-Reelin antibody (Abcam 78540) | Ms | 1:1000 |
| Anti-β III Tubulin antibody (Abcam #ab78078) | Ms | 1:300 |
| Anti-Doublecortin antibody (EMD Millipore #AB2253) | G.Pig | 1:1000 |
| Anti-Calretinin antibody (Abcam #ab702) | Rb | 1:100 |
| Anti-BrdU antibody (Serotec #MCA2060) | Rat | 1:200 |
| Anti-Neuro-Chrom Pan Neuronal Marker (Millipore #MAB2300) | Ms | 1:250 |

| **Secondary Antibodies** |
|---|
| Goat Anti-Mouse IgG H&L (Alexa Fluor® 568) (ab175473) |
| Goat Anti-Mouse IgG H&L (Alexa Fluor® 488) (ab150117) |
| Goat Anti-Rabbit IgG H&L (Alexa Fluor® 488) (ab150077) |
| Goat Anti-Rabbit IgG H&L (Alexa Fluor® 568) (ab175471) |
| Goat Anti-Guinea pig IgG H&L (Cy5.5 ®) (ab6967) |

*Excel files:*

**Supplementary Table 2**: List of all genes expressed in analyzed triplicate samples. RNAseq was done in three biological replicates of: (i) NPC, (ii) NCC; (iii) NPC + FGFR1(SP-/NLS)(TK-), (iv) NCC + FGFR1(SP-/NLS)(TK-) and (v) NCC + FGFR1(SP-/NLS).

**Supplementary Table 3**: Lists of genes significantly affected between NPC and NCC.

**Supplementary Table 4**: List of NPC genes significantly affected by FGFR1(SP-/NLS)(TK-).

**Supplementary Table 5**: List of NCC genes significantly affected by FGFR1(SP-/NLS)(TK-).

**Supplementary Table 6**: List of NCC genes significantly affected by FGFR1(SP-/NLS).

**Supplementary Table 7**: List of all FGFR1(SP-/NLS)(TK-) or FGFR1(SP-/NLS) affected genes that were previously shown [18] to bind nFGFR1 in developing NCCs differentiated from iPSCs of control and schizophrenia patients.
